# Rapid Histone Post-Translational Modification Analysis Using Alternative Proteases and Tandem Mass Tags

**DOI:** 10.64898/2026.02.13.705817

**Published:** 2026-02-15

**Authors:** Natalie P. Turner, Sabyasachi Baboo, Patrick Garrett, Jolene K. Diedrich, Michael Bajo, Marisa Roberto, John R. Yates

**Affiliations:** 1 The Scripps Research Institute, Department of Integrative and Structural Computational Biology, 10550 North Torrey Pines Rd, La Jolla, CA 92037, United States of America; 2 The Scripps Research Institute Multi-Omics Core Facility, 10550 North Torrey Pines Rd, La Jolla, CA 92037, United States of America; 3 The Scripps Research Institute, Department of Translational Medicine, 10550 North Torrey Pines Rd, La Jolla, CA 92037, United States of America

## Abstract

Histone post-translational modifications (PTMs) alter chromatin dynamics and contribute to the regulation of gene expression in health and disease. Mass spectrometry-based analysis is the gold-standard for histone PTM analysis, but it remains constrained by inefficient sample preparation workflows requiring multiple days. Here, we develop RIPUP (*R*apid *I*dentification of histone *P*TMs in *U*nderivatized *P*eptides), a streamlined multi-protease workflow that reduces sample preparation from days to hours while improving PTM coverage and quantitative accuracy. Through systematic evaluation of the Arg-C Ultra protease and a prototype recombinant (r)-Chymotrypsin protease under varied conditions, such as chemical derivatization using propionic anhydride and tandem mass tags (TMT), we demonstrated that Arg-C Ultra with TMT labeling achieves a detection of total PTM comparable to conventional Trypsin-based approaches. Using the HiP-Frag computational framework for unrestrictive PTM identification, we discovered that TMT’s tertiary amine provides charge compensation that rescues the ionization of negatively charged acylations revealing 50 succinylation and 27 glutarylation sites – a ‘dark epigenome’ largely undetected by propionylation-based methods. We demonstrated that complementary digestion with Arg-C Ultra and r-Chymotrypsin provides orthogonal sequence coverage, enabling detection of PTMs in H2A variants, linker histones, and regions poorly represented by arginine-specific cleavage alone. Application of RIPUP to frozen-thawed rat hippocampal sections within a 3-hour workflow identifies >200 PTMs including biologically critical PTM sites H3 K27/K36/K37 methylation, H4 N-terminal acetylation patterns, and H2A ubiquitination at K118/K119. This rapid, high-efficiency platform enables timely discovery of epigenetic mechanisms and accelerates the path from PTM identification to therapeutic target validation.

## Background

Mass spectrometry (MS)-based proteomics is a popular and powerful tool for analyzing histone post-translational modifications (PTMs), known as the ‘histone code’ ^1,2^. Histone proteins assemble as octamers comprised of two copies of histone H2A, H2B, H3 and H4 to form the nucleosome core, which is sequentially wrapped in ~146–147 bp DNA during nucleosome assembly ^3^. Tightly wound nucleosomes form chromatin, the major structural unit of chromosomes. Fundamental aspects of epigenetic regulation are under the control of ‘writers’ and ‘erasers’ that add and remove chemical groups (PTMs) to histones. These changes control gene transcription by altering chromatin structure and thus the accessibility of transcriptional machinery to chromatin. Histone PTM research has focused on understanding nuanced epigenetic mechanisms implicated in health and disease paradigms, including cancer ^3^, neurobiology ^4^, addiction ^5^, and the gut microbiome ^6^.

Garcia et al. first described a workflow that significantly advanced the field of histone PTM analysis by MS in 2007 ^2^. As histones are lysine (K)- and arginine (R)-rich, traditional bottom-up proteomics with Trypsin cleavage produces mostly short peptides that are not within the appropriate mass or retention time range for detection by liquid chromatography-tandem mass spectrometry (LC-MS/MS). Hence, Garcia et al. (2007) ^2^ designed a workflow that includes a chemical derivatization step to propionylate ε-amino groups of lysine (K). This modification prevents Trypsin cleavage after K, generating longer, more hydrophobic peptides which improves solid-phase retention and chromatographic separation ^2,7^. These tryptic peptides contain R as the C-terminal amino acid and produce stable, singly charged *y*-ion series fragments. While this workflow has undergone multiple iterations since its initial publication ^7,8^, alternative proteases and the optimization of parameters that may improve workflow efficiency, peptide sequence coverage, identify novel PTMs, improve reproducibility, or lower peptide coefficients of variation (CVs) is still relatively under-explored ^9^.

Two new-to-market enzymes – a prototype recombinant (r)-Chymotrypsin and Arg-C Ultra (Promega^™^) – offer significant advantages to the standard Trypsin and propionylation approach for histone PTM analysis by MS-based proteomics. For example, Trypsin digestion of histone proteins labeled with propionic anhydride should produce an Arg-C Ultra-like cleavage pattern (cleavage at the C-terminus of arginine), but labeling efficiency can be user-dependent and is prone to variability ^10,11^, which reduces the overall number of peptides that can be reliably used for quantitation. Additionally, r-Chymotrypsin and Arg-C Ultra require shorter incubation times (2 h compared to >6 h with Trypsin). Importantly, if derivatization is preferred for its chromatographic benefits, propionylation is required after digestion only to label unmodified lysines and peptide N-termini, as neither Arg-C Ultra nor r-Chymotrypsin cleaves at the lysine C-terminus, so that the cleavage specificity of the proteases is unaffected by lysine modification status. In contrast to standard Chymotrypsin, the prototype r-Chymotrypsin does not cleave after tryptophan (W) and shows increased specificity for leucine (L), tyrosine (Y), and phenylalanine (F), generating peptides not seen with Trypsin and Arg-C Ultra digestion, thus capturing different segments of histone protein sequences. These changes to the established protocol ^1,2^ can significantly reduce the overall sample preparation time for development of high-throughput workflows and reveal insights into histone PTMs not seen with Trypsin digestion ^9^.

While elimination of the propionylation step reduces overall sample preparation time, addition of propionyl groups to tryptic peptides increases their overall hydrophobicity, which is advantageous for retention of short, hydrophilic peptides. However, separating peptidoforms with isobaric PTMs, similar retention times or small mass differences remains a challenge regardless of whether or not they are propionylated. Other approaches for labeling histone peptides have been reported, including recent work by Ryzhaya et al., which demonstrated that the Arg-C Ultra protease, which has substantially better arginine-cleavage specificity and efficiency than conventional Arg-C, can be combined with peptide-level derivatization using trimethylacetic anhydride (TMA) to reduce histone sample preparation time to ~3–4 hours ^9^. Interestingly, tandem mass tags (TMT) ^12^ is a well-established amine-reactive label that has not been systematically evaluated for histone PTM analysis. TMT-labeling adds isobaric tags to primary amines, such as those of post-digestion peptide N-termini and unmodified K residues. Like propionyl labeling, TMT labeling creates peptides that are more hydrophobic, but with potentially higher labeling efficiency in a single one-hour incubation step. Importantly, the unique chemical structure of TMT, which includes a tertiary amine within the reporter region, may confer previously unrecognized advantages for detecting specific PTM classes that reduce the overall charge state of the peptide, such as negatively charged acylations, succinylation and glutarylation ^13,14^. Additionally, TMT is compatible with multiplexed data acquisition and may offer benefits for quantitative analysis.

Recent advances in computational mass spectrometry have revealed that the histone PTM landscape is substantially more complex than previously appreciated. Vai et al. (2025) ^15^ recently developed HiP-Frag, an unrestrictive search workflow that identified 60 previously unreported modifications on core histones and 13 on linker histones across multiple cell lines and tissue samples. Their work demonstrated that rigorous computational filtering at the peptide-to-spectrum match (PSM) and peptide levels coupled with detailed mass offset search—rather than exhaustive validation using synthetic peptides—can enable confident discovery of novel PTMs at scale, expanding our understanding of the histone code beyond classical acetylation and methylation. However, while these computational advances have revealed the existence of numerous uncommon modifications, the key question of whether different sample preparation and chemical labeling strategies preferentially detect certain PTM classes remain unaddressed and has implications for potentially biasing our view of the epigenetic landscape. This consideration is particularly important given that propionylation neutralizes positive charges on lysine residues, which may affect the ionization efficiency of peptides bearing negatively charged modifications.

**Figure 1.**
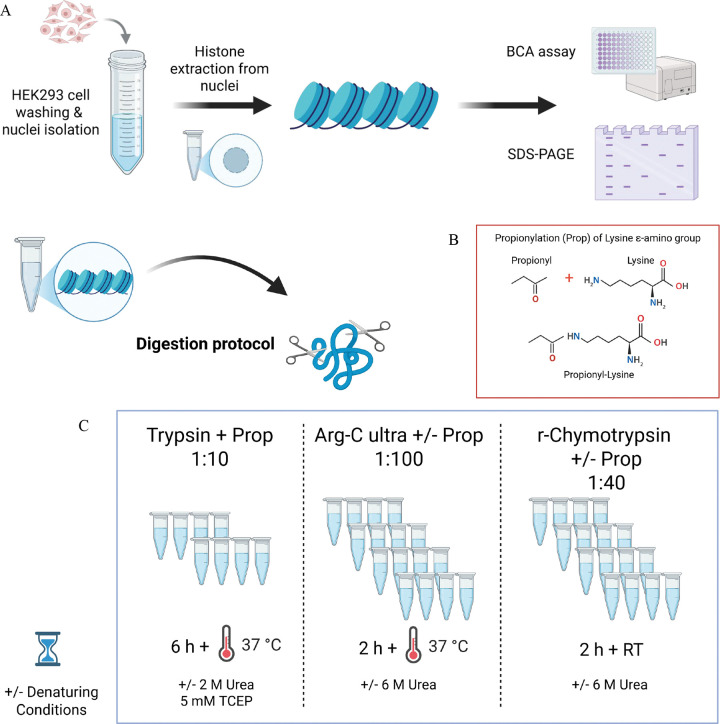
Experimental workflow for histone extraction and quality control from cell culture samples. HEK293T cells (A) were washed, homogenized, and processed for nuclei isolation, followed by acid extraction of histones from purified nuclei. Extracted histones from both sample types were quantified using bicinchoninic acid (BCA) protein assay and assessed for purity by SDS-PAGE to confirm successful extraction and appropriate histone band patterns before downstream proteomic analysis. Extracted histones were subjected to enzymatic digestion using three different protease conditions with or without chemical propionylation (Prop) and denaturing agent (2 or 6 M urea). (B) Chemical reaction showing propionylation of lysine ε-amino groups, which neutralizes positive charges but prevents Trypsin cleavage at lysine residues, enabling retention of shorter peptides for higher sampling of histone PTMs. (C) Digestion conditions for systematic evaluation of Trypsin, Arg-C Ultra, and r-Chymotrypsin. Conventional Trypsin-based workflow with propionylation at 1:10 enzyme-to-substrate ratio, requiring 6 hours incubation at 37 °C. Samples were prepared with or without denaturing conditions. Arg-C Ultra digestion at 1:100 enzyme-to-substrate ratio with or without propionylation, requiring only 2 hours at 37 °C. Prototype recombinant chymotrypsin (r-Chymotrypsin) digestion at 1:40 enzyme-to-substrate ratio with or without propionylation, performed at room temperature (RT) for 2 hours. All conditions were systematically evaluated for digestion efficiency, labeling completeness, PTM coverage, and generation of informative peptides suitable for quantitative PTM analysis by mass spectrometry.

To address these challenges, we performed a comprehensive systematic evaluation of both the standard Trypsin protocol ^1^ and two alternative proteases, Arg-C Ultra and a commercially available prototype recombinant (r)-Chymotrypsin (Promega^™^), under multiple experimental conditions using histones extracted from HEK293T cells. We tested each protease with and without propionylation, under denaturing and non-denaturing conditions, generating 10 distinct conditions (Figure 1). We also assessed TMT labeling as an alternative to derivatization with propionic anhydride. This systematic approach allowed us to assess digestion efficiency, protein sequence coverage, labeling efficiency, and identification of common histone PTMs under rigorously controlled conditions. In addition to confirming recent reports of Arg-C Ultra’s superior specificity, our analysis revealed that TMT labeling provides unique advantages for detecting negatively charged acylations through charge compensation at the peptide N-terminus. We further demonstrated that r-Chymotrypsin provides complementary sequence coverage for H2A variants and linker histones that are poorly represented by arginine-specific cleavage. As a proof-of-concept, we performed our streamlined dual-protease protocol, RIPUP (Rapid Identification of histone PTMs in Underivatized Peptides), on histones extracted from rat hippocampal sections to identify peptidoforms and PTM sites of biological interest in under 3 hours of sample preparation time.

## Methods

Adult male Sprague-Dawley rats used in this study (Charles River Laboratories, Raleigh, NC) were kept in accordance with the ARRIVE guidelines and were approved by the Scripps Research Institute (TSRI) Animal Care and Use Committee (IACUC #09–0006), consistent with the National Institutes of Health Guide for the Care and Use of Laboratory Animals. The animals were housed in a temperature- and humidity-controlled room (12 h reverse light cycle) and provided with food and water *ad libitum*. The rats (n = 5, 446 ± 17.8 g) were anesthetized with isoflurane (3%), decapitated, and the brains were rapidly removed and placed into ice-cold high-sucrose cutting solution (206.0 mM sucrose, 2.5 mM KCl, 0.5 mM CaCl2, 7.0 mM MgCl2, 1.2 mM NaH2PO4, 26 mM NaHCO3, 5.0 mM glucose, and 5 mM HEPES) gassed with 95% O2 and 5% CO2 ^16,17^. A Vibrotome VS1000 (Leica Microsystems) was used to cut 300 μm coronal slices containing hippocampus (AP: −2.00 to −3.25 from bregma). The slices were transferred to cold, oxygenated (95% O2 and 5% CO2) artificial cerebrospinal fluid (130 mM NaCl, 3.5 mM KCl, 1.25 mM NaH2PO4, 1.5 mM MgSO4, 2 mM CaCl2, 24 mM NaHCO3, and 10 mM glucose) and hippocampi were isolated and collected in 1.5 mL microcentrifuge tubes. Following isolation, hippocampi were immediately snap-frozen by placing the sample tubes into dry ice and transferred to cold storage at −80 °C until sample processing.

### Histone extraction

Histones were extracted from HEK293T cells using an established protocol ^7^ (Figure 1). Briefly, frozen aliquots of HEK293T cells stored in Dulbecco’s Complete Media supplemented with 5% DMSO were thawed at 37 °C in a water bath. Cells were pooled by transferring frozen-thawed aliquots to a 50 mL falcon tube and topped with DPBS warmed to 37 °C. Cells were washed twice by centrifugation at 300 ×*g* for 5 min at RT and the cell pellet was resuspended in chilled nuclear isolation buffer (NIB) ^7^ with DTT and protease inhibitors (Pierce EDTA-free protease inhibitor cocktail, ThermoScientific), then washed by centrifugation at 700 ×*g* for 5 min, 4 °C. The supernatant was aspirated, and the remaining cell pellet was lysed with NIB containing 0.2% NP-40 alternative and homogenized by gently pipetting up and down. The mixture was incubated on ice for 10 min and centrifuged at 1000 ×*g* for 10 min at 4 °C. The supernatant (cytoplasmic fraction) was aspirated and stored at −80 °C. The nuclei pellet was washed three times with NIB by centrifugation at 1000 ×*g* for 5 min at 4 °C to remove traces of detergent, and the supernatant was discarded after the final wash. For rat hippocampal sections, frozen samples were thawed on ice, washed once with chilled NIB, transferred to a 2 mL dounce homogenizer, and homogenized/lysed with 10 – 15 strokes of the fine pestle. All other nuclei isolation steps were the same as described for nuclei extraction from HEK293T cells.

Acid extraction of histones from the nuclei pellets was performed in 0.2 M H_2_SO_4_ and the mixture was incubated at 4 °C for 2–3 h with gentle rotation. The mixture was centrifuged at 3,400 ×*g* for 5 min at 4 °C and the supernatant containing extracted histones was transferred to a new 1.5 mL microcentrifuge tube. Centrifugation was repeated to remove any traces of insoluble material. Trichloroacetic acid (TCA; 100%) was added to the supernatant to a final concentration of 33%, vortexed to mix, and incubated on ice overnight to precipitate histones. The next day, the suspension was centrifuged at 3,400 ×*g* for 5 min at 4 °C. The supernatant was aspirated and the pellet washed with ice-cold acetone/0.1% HCl. Centrifugation was repeated at 3,400 ×*g* for 2 min, supernatant was aspirated, and the pellet was washed once more with 100% ice-cold acetone. The acetone supernatant was aspirated and the pellet left to dry briefly. The histone pellets were resuspended in de-ionized H_2_O, vortexed to mixed, and centrifuged at 3,400 ×*g* for 2 min at 4 °C to pellet insoluble material. The supernatant was transferred to a new tube and assessed for quality by SDS-PAGE and protein concentration by BCA assay (Pierce^™^ BCA Protein Assay, cat number 23227, Thermo Scientific^™^). For HEK293T samples, the sample was divided into aliquots of ~5 μg protein according to Tables 1 and 2, with four technical replicates in each condition, resulting in a total of 40 samples.

### SDS-PAGE

Aliquots of 5 μL extracted histones were mixed with 4x LDS sample buffer (NuPAGE^™^, Catalog number NP0007, Invitrogen^™^) and 10x sample reducing agent (NuPAGE^™^, Catalog number NP0009, Invitrogen^™^), to achieve a 1x final concentration in the sample. Samples were reduced for 10 min at 70 °C and 450 rpm in a Thermomixer (ThermoScientific). A volume equivalent to 2 μg total protein of purified histone H2A standard (Sigma-Aldrich, Cat number H9250, Sigma-Aldrich) was prepared in the same way. A 5 μL aliquot of protein ladder (BLUEstain^™^ 2 Protein ladder, 5–245 kDa, Cat Number: P008–500, Goldbio), samples, and H2A standard were separated by gel electrophoresis on a 4–12% Bis-Tris mini protein gel, 1.0–1.5 mm (NuPAGE^™^, Cat number: NP0321BOX, Invitrogen^™^) for 50 min at 150 V. The protein gel was placed into a clean gel tray and incubated with Acquastain gel stain (Bulldog Bio) to visualize protein bands. Gels were imaged on a gel imager with 5 s exposure (Azure biosystems c600).

### MS sample preparation

**Figure 2.**
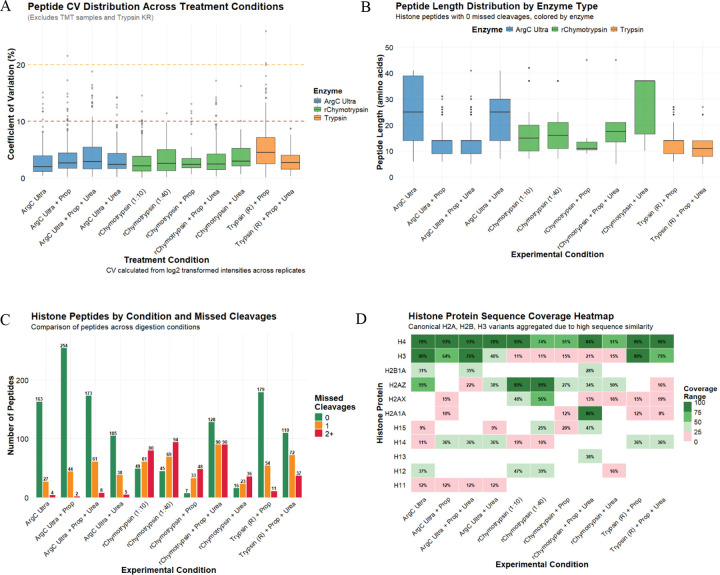
Comparative analysis of enzymatic digestion and propionylation strategies for histone post-translational modification detection in HEK293T cells (*n* = 4/condition). (A) Peptide coefficients of variation (CV) by enzyme type. All enzymes yield CVs < 10 %. (B) Peptide length distributions for peptides with 0 missed cleavages. Propionylation facilitates identification of shorter peptides. (C) Number of histone peptides identified across experimental conditions, stratified by missed cleavages (0, 1, or 2+). Arg-C Ultra consistently yields the highest peptide counts with minimal missed cleavages. (D) Sequence coverage heatmap for canonical histone proteins across experimental conditions. H3 variants (H3.1, H3.2, H3.3) are aggregated due to high sequence similarity. Coverage values represent percentage of protein sequence covered by identified peptides.

We evaluated two different workflows against the established Trypsin with peptide N-terminal and K derivatization protocol: denaturing vs. non-denaturing and derivatized vs. non-derivatized for Arg-C Ultra and r-Chymotrypsin (Table 2 and Figure 2). We also introduced TMT-labeling of Arg-C Ultra and r-Chymotrypsin digested peptides as an alternative to derivatization with propionic anhydride.

#### Protease digestion

Extracted histone samples were subjected to proteolytic digest with Arg-C Ultra or r-Chymotrypsin in 100 mM ammonium bicarbonate (AMBIC) under the conditions shown in Tables 1 and 2, according to the manufacturer’s recommendations. The pH of all buffers were tested using pH strips (Cat Number: 13640521, Fisher Scientific) to ensure the pH was maintained at 8–8.5. Digestions with Arg-C Ultra (1:100) and r-Chymotrypsin (1:40 or 1:10) were performed in 10 μL reactions at 37 °C and RT, respectively, for 2 h in a thermal cycler (Biorad, MJ Mini). For histones extracted from rat hippocampi, we used Arg-C Ultra (1:10) and r-Chymotrypsin (1:10) in 20 μL reactions.

When labeling with TMT, HEK293T histones (5 μg) were digested in 100 mM TEAB pH 8.5, with Arg-C Ultra (1:100) or r-Chymotrypsin (1:10).

#### Derivatization (Propionylation)

Samples belonging to the Trypsin digestion groups were propionylated prior to digestion to prevent cleavage at the C-terminal of unmodified K residues. Samples were suspended in 50 mM AMBIC pH 8.0 to a final volume of 20 μL. The propionylation reagent was prepared as previously described ^1^ and all propionylation steps were performed in a fume hood. In brief, propionylation reagent was prepared by combining propionic anhydride with 100% ACN at a 1:3 (*v/v*) ratio. One batch of proprionylation reagent was used for up to 4 samples. Following the addition of propionylation reagent (1:4 *v/v* reagent to sample ratio), the pH was restored to pH 8–8.5 with the addition of ammonium hydroxide (30% *w/v*). Samples were vortexed to mix and incubated at RT for 15 min. A second round of propionylation was performed, and after the final incubation step, samples were dried in a vacuum centrifuge and stored at −80 °C until proteolytic digestion. Dried samples were resuspended in 10 μL digestion buffer containing Trypsin (Trypsin Gold, Mass Spectrometry Grade, Promega, V5280) in 100 mM AMBIC pH 8.5, at an enzyme:substrate ratio of 1:10. For the ‘Trypsin + Urea’ group, the digestion buffer also contained 2 M Urea and 5 mM TCEP (Table 2). Digestion reactions were incubated at 37 °C in a Thermomixer (Eppendorf) for 6 h. The reaction was stopped by freezing at −80 °C. The next day samples were thawed and dried in a vacuum concentrator. Two more rounds of propionylation were performed on Trypsin-digested samples and samples digested with Arg-C Ultra and r-Chymotrypsin (volumes adjusted with ddH_2_O to final concentration 50 mM AMBIC) to label peptide N-termini and remaining K residues (Table 1). Finally, propionylated peptides were dried and resuspended in 10 μL 0.1% formic acid (FA) in de-ionized water.

#### Tandem Mass Tag (TMT) labeling

Histone peptides suspended in ddH_2_O were mixed with TEAB to a final concentration of 100 mM, pH 8.0. Arg-C Ultra-generated peptides were labeled with TMT^10^-126, and r-Chymotrypsin generated peptides were labeled with TMT^10^-131 (cat no 90309, Thermo Scientific; monoisotopic mass = 229.162932) as per the manufacturer’s instructions (peptide:TMT ratio 1:8, final concentration of anhydrous acetonitrile = 44%) for 1 h at RT in a 9 μL reaction. Free TMT was quenched by adding 1 μL of 5% hydroxylamine to the reaction and incubating for 15 min at RT. Labeled peptides were dried in a vacuum concentrator and resuspended in 0.1% FA in de-ionized water.

Peptide concentration was determined in samples digested with Trypsin (non-denaturing conditions) by colorimetric peptide assay (Pierce Colorimetric Peptide Assay Kit, Thermo Scientific). Peptide samples were diluted 1:100 with 0.1% FA, and 20 μL (~ 50 ng) aliquots were loaded onto Evotips (Evosep) following the manufacturer’s instructions.

### LC-MS/MS analysis

Mass spectrometry analysis was performed using a Thermofisher Scientific Fusion Lumos Tribrid Mass Spectrometer configured with an electrospray ionization (ESI) source and operated in positive ion mode. The instrument was interfaced with an Evosep One nanoLC system (Evosep). The mobile phase comprised Solvent A (H_2_O with 0.1% FA) and Solvent B (ACN with 0.1% FA) (LC-MS grade, Fisher Scientific).

Reversed-phase HPLC separation was achieved using a custom-packed analytical capillary column (25 cm length, 150 nm internal diameter) containing Waters BEH C18 resin (1.7 μm particle size). Eluted peptides were introduced into the mass spectrometer via nanoelectrospray with a 2 kV spray voltage applied to the column inlet. Peptide fragmentation was performed using High-Energy Collisional Dissociation (HCD) in the Orbitrap. For non-TMT peptides, a fixed collision energy of 30% was applied, while TMT-labeled peptides were fragmented using a stepped normalized collision energy (NCE) of 30%, 40%, and 50% ^18^. The analytical method employed a 15 spd LC gradient (88 minutes) at 220 nL/min.

#### DDA

Full MS scans were collected at 120K resolution in the Orbitrap over a scan range of 375 – 1500 *m*/*z* in profile mode. Default charge state was set to +2, cycle time was 3 s, and maximum injection time was 50 ms. Included charge states were +2 to +7, dynamic exclusion was set to 5 s, and precursor mass tolerance was set to 10 ppm. All precursors above the minimum intensity of 5e^4^ during the 3 s cycle time, or up to the AGC target of 4e^5^ ions, were selected for HCD MS/MS scans in the Orbitrap at 7.5K resolution and collected as centroided data. Maximum injection time for MS/MS scans was set to 100 ms, with an AGC target of 5e^4^. Isolation was performed in the quadrupole with an isolation window of 1.6 *m*/*z*.

### Data analysis

#### Protein and PTM identification

MS raw files were processed in FragPipe (v24.0) following the recommended guidelines for the HiP-Frag workflow, with some modifications ^15^. Data were searched against a restricted database containing extracted human or rat histone sequences, contaminants and decoys (*Homo sapiens*: 342 sequences, 171 decoys; *Rattus norvegicus*: 292 entries, 146 decoys; contaminants lists were derived from and curated by Cambridge Centre for Proteomics (CCP) cRAP). The enzyme cleavage parameters were adjusted for Arg-C Ultra and r-Chymotrypsin to cleave after R or FLYM, respectively. Up to 2 missed cleavages were allowed for Arg-C Ultra and Trypsin (set to cleave after R only) and up to 3 missed cleavages were allowed for r-Chymotrypsin. An additional Trypsin search was conducted using the default cleavage specificity (KR) and missed cleavages (5) as an additional assessment of the effects on peptide diversity resulting from incomplete propionylation of internal K residues. N-terminal propionylation was enabled as a static modification for all propionylated samples. For propionylated and non-propionylated samples, propionylation was set as a variable modification on K to account for endogenous propionylation in the case of the latter ^19^. All default variable modifications and mass offsets were enabled as per default pipeline settings, except methylation (+14.01565 Da) was added as a variable modification to non-propionylated samples (default setting +70.0419 Da, the combined mass of a propionyl group and methyl group), and the +70.0419 Da mass was retained as endogenous butyrylation. Propionylation on serine, threonine, and tyrosine was removed from the detailed mass offset list for non-propionylated samples. For TMT-labeled samples, the monoisotopic mass of the intact label (+229.162932 Da) was set as a static modification on peptide N-termini and as a variable modification on K, and all other PTM declarations were consistent with unlabeled samples. Finally, there were specific mass shifts considered for Gly-Gly (GG; +114.0429 Da) or Arg-Gly-Gly (RGG; +270.1441 Da) remnants corresponding to cleavage of ubiquitinylated K (Arg-C Ultra and r-Chymotrypsin cleaved ubiquitin, respectively. These fragments can also be labeled with propionyl or TMT at the free amine at the N-terminus, so +170.0691 Da (GG + Prop) and +343.2058 Da (GG + TMT) for Trypsin or Arg-C Ultra-digested samples, and +326.1703 Da (RGG + Prop) and +499.307 Da (RGG + TMT) for r-Chymotrypsin-digested samples were included. Label-free quantification (LFQ) and match-between-runs (MBR) were enabled for quantification. The propionyl and TMT masses were removed from peptidoforms during data analysis to enable direct qualitative comparison of identified peptidoforms between conditions.

#### Data processing and statistical methods

Data resulting from HiP-Frag output were imported into RStudio (RStudio 2025.09.2+, Build 418) and analyzed using custom R scripts. Reproducibility was assessed by calculating coefficients of variation (CV) from log2-transformed peptide intensities across technical replicates (*n* = 4 per condition for HEK293T samples; *n* = 5 for rat hippocampal samples). Digestion efficiency was evaluated by quantifying the proportion of peptides with 0, 1, or ≥2 missed cleavages. Labeling efficiency was calculated for both propionylation and TMT derivatization using two complementary metrics. For each peptide, lysine residues were classified based on modification status: labeled (bearing the expected derivatization mass: +56.026 Da for propionyl; +229.163 Da for TMT), free (unmodified), or biologically modified (e.g., acetylation, methylation). Biologically modified sites were excluded from calculations as they are not substrates for chemical derivatization. Site-based efficiency was calculated as labeled sites divided by the sum of labeled and free sites, expressed as a percentage (Efficiency (by site) = [labeled sites / (labeled sites + free sites)] × 100). Intensity-weighted efficiency was calculated by weighting each site count by peptide MS1 intensity, accounting for relative peptide abundance (Efficiency (by intensity) = [Σ(labeled sites × intensity) / (Σ(labeled sites × intensity) + Σ(free sites × intensity))] × 100). For TMT samples, both lysine residues and peptide N-termini were evaluated; for propionylation, only lysine residues were considered. Informative peptides were defined as those that were fully labeled, contained ≤1 missed cleavage, and were detected in ≥3 replicates. Histone protein sequence coverage was calculated as the percentage of theoretical amino acid sequence represented by identified peptides. PTM diversity was quantified by counting unique modification types at specific residue positions (e.g., H4 K8ac K12ac = 2 acetylations). For histones extracted from frozen-thawed rat hippocampal sections processed using the RIPUP protocol, peptidoforms were retained if detected in ≥2 replicates. Data are presented as means ± standard deviation or medians where appropriate.

## Results

### Protease and digestion conditions comparison

To assess potential improvements over the conventional Trypsin digestion with propionylation derivatization protocol, we systematically evaluated two alternative proteases, Arg-C Ultra and r-Chymotrypsin, under various experimental conditions including propionylation (Prop), urea denaturation, and TMT as an alternative to propionylation labeling. Our analysis focused on key performance metrics including total peptide identifications, digestion efficiency, sequence coverage, and the generation of informative peptides suitable for PTM identification and quantification. The reproducibility of protease activity and subsequent analyses via the developed pipeline were found to be robust, with median peptide CVs < 5% for all conditions (Figure 2A).

The three proteases tested generated peptides with distinct length distributions when considering complete cleavage (Figure 2B). Short/hydrophilic peptides generated by protease action are often lost during chromatographic separation resulting from poor solid-phase retention, explaining over-representation of longer peptides (median peptide length >15 amino acids) when treating non-propionylated substrates with proteases. Further denaturation of substrates by urea increases the length range of identified peptides generated by r-Chymotrypsin, whose protease activity is known to be sensitive to any remnant secondary structures ^20^. In contrast, propionylation improves the hydrophobicity of short peptides, which reduces the median length of identified peptides in propionylated samples considerably, irrespective of urea denaturation. Trypsin and Arg-C Ultra showed similar cleavage specificity on propionylated histones, with a similar median peptide length of ~15 amino acid in both conditions that was unaffected by the presence of urea.

Arg-C Ultra digestion yielded substantially higher peptide identifications than the conventional ‘Trypsin + Prop’ approach (Figure 2C). Considering only the peptides with 0 missed cleavages, digestion with Arg-C Ultra resulted in the identification of 163 distinct histone peptides. Additional propionylation dramatically improved peptide identification to 254 distinct histone peptides, likely due to increased hydrophobicity, peptide retention and separation during LC. Notably, Arg-C Ultra demonstrated superior digestion efficiency with most identified peptides showing no missed cleavage (~84%, with and without propionylation, in absence of urea), whereas ‘Trypsin + Prop’ produced a more heterogeneous mixture containing peptides with one or more missed cleavages. The addition of urea generally increased the number of missed cleavages across all enzyme types, though this effect was minimal with Arg-C Ultra (~70% peptides without missed cleavage), and urea decreased the total number of peptides generated when the substrate was not propionylated. This could be attributed to the denaturing effect of urea being more pronounced on the protease activity than on the substrate that is already denatured by acid extraction.

r-Chymotrypsin followed similar trends, though fewer peptides were generated than Arg-C Ultra and higher missed cleavage rates were observed. r-Chymotrypsin performed similarly at the 1:10 and 1:40 enzyme-to-substrate ratios (49 and 45 peptides with no missed cleavages, respectively i.e., ~20–25% peptides). In the presence of urea, fewer peptides were generated, though fully cleaved peptides still formed ~21% of the total. However, in contrast to Arg-C Ultra, propionylation decreased the number of peptides generated with r-Chymotrypsin: only 7 fully cleaved peptides (~8% peptides) were identified. In contrast to Arg-C Ultra, urea boosted overall digestion and fully cleaved peptide numbers of propionylated histone peptides to 128 (~42% peptides), though substantially increasing missed cleavages.

### Histone protein sequence coverage

We assessed the impact of different enzymatic digestion strategies on histone protein sequence coverage, with histone sub-variants aggregated due to high sequence similarity (Figure 2D). Core histone H4 exhibited the highest sequence coverage across all conditions, with ~75–95% coverage consistently achieved across most conditions. H3 coverage was highest (80%) with Arg-C Ultra and ‘Trypsin + Prop’, however sequence coverage provided by r-Chymotrypsin was low (~10–20%). r-Chymotrypsin provided high sequence coverage for H2A variants, H2A.Z (95%, when used at 1:40) and H2A1A (86% with ‘r-Chymotrypsin + Prop’) compared to minimal or no sequence coverage with Arg-C Ultra and Trypsin. Linker histone H1 variants (H1.1, H1.2, H1.3, H1.4, H1.5) exhibited variable coverage across enzyme types, with r-Chymotrypsin outperforming Arg-C Ultra and Trypsin in most cases except H1.4 where Arg-C Ultra and Trypsin perform better. H1.2 and H1.5 were covered up to 47% with r-Chymotrypsin. The complementary coverage patterns observed across the three enzymatic approaches underscores the value of employing orthogonal digestion strategies to maximize histone proteome characterization and PTM site accessibility.

### Chemical Rationale for TMT Labeling

**Figure 3:**
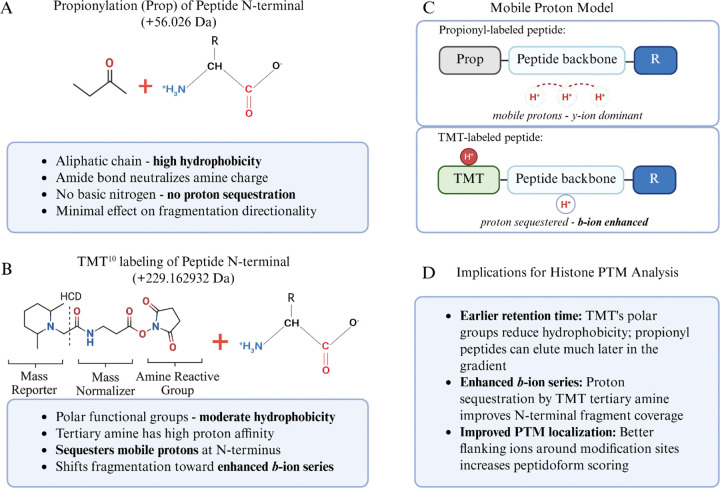
Chemical and structural comparison of propionylation and TMT derivatization strategies for histone peptide analysis. A: Propionylation of peptide N-terminal amines using propionic anhydride (+56.026 Da). The propionyl group is a small aliphatic chain that increases hydrophobicity and neutralizes the amine charge but contains no basic nitrogen capable of proton sequestration. B: TMT^10^ labeling of peptide N-terminal amines (+229.1629 Da). The TMT label comprises three functional regions: a mass reporter containing a tertiary amine with high proton affinity, a mass normalizer incorporating heavy isotopes (e.g., ^13^C, ^15^N), and an amine-reactive NHS ester group. The tertiary amine within the reporter region can sequester mobile protons during collision-induced dissociation. C: Mobile proton model illustrating differential fragmentation behavior. In propionyl-labeled peptides, protons remain mobile along the peptide backbone, favoring *y*-ion formation. In TMT-labeled peptides, the tertiary amine sequesters a proton at the N-terminus, shifting fragmentation dynamics toward enhanced *b*-ion series generation. D: Summary of implications for histone PTM analysis. Image of TMT structure in (B) was adapted from Thermo Scientific Pub. No. MAN0016969, Rev B.0, Pub. Part No. 2162457.5 (Figure 2). Created in https://BioRender.com.

In this study, we used TMT labels as derivatization agents because of their hydrophobicity and high labeling efficiency, independent of their conventional use as multiplexed isobaric labels for quantitating conditional changes in protein abundance ^12^. TMT and propionyl derivatization both target primary amines (N-termini and K ε-amines) via NHS ester and anhydride chemistry, respectively, forming stable amide bonds ^2^. However, the structural differences between these modifications impact peptide behavior during LC-MS/MS. Propionylation introduces a small aliphatic acyl group (+56.026 Da) that increases peptide hydrophobicity due to its purely hydrocarbon character (Figure 3A and 3B). This results in extended retention times on reversed-phase columns and can still result in co-elution of multiply modified histone peptides, particularly those derived from the K-rich N-terminal tails of histones H3 and H4. In contrast, TMT labels (~229.1629 Da for TMT^10^) have multiple polar functional groups, including carbonyl oxygens and a tertiary amine within the reporter region. This structural composition confers moderate rather than high hydrophobicity, resulting in earlier retention times than propionylated counterparts. Propionylated histone peptides, particularly those with multiple modified K residues, can elute late in the reversed-phase gradient where chromatographic resolution may be suboptimal.

Importantly, the tertiary amine within the TMT reporter region shows high proton affinity. According to the mobile proton model of peptide fragmentation ^21^, protons migrate along the peptide backbone during collision-induced dissociation and direct bond cleavage. In conventional tryptic and Arg-C-like peptides, mobile protons preferentially localize toward the C-terminus, favoring *y*-ion formation (Figure 3C) ^22^. The TMT tertiary amine sequesters a mobile proton at the N-terminal region of the peptide, shifting fragmentation dynamics to enhance *b*-ion series generation. This effect was particularly pronounced when using stepped collision energies of 30, 40, and 50 (normalized), which provided sufficient energy to fragment the peptide backbone while the TMT moiety retained the sequestered proton.

The enhanced *b*-ion coverage has direct implications for histone PTM analysis, where confident localization of PTMs requires the presence of flanking fragment ions on both sides of the modification site (Figure 3D). For histones, where multiple modifications often occur in close proximity on the same peptide, the enhancement of *b*-ions provided by TMT improves site localization confidence and peptidoform discrimination (Supplementary Table S3).

### Initial TMT assessment

Current methods of histone PTM analysis rely on efficient labeling/chemical propionylation of K and peptide N-termini to ensure robust PTM identification, quantification, and reliable comparison between two or more groups ^8,10,23^. However, it is well-documented that propionylation can be highly variable, with both under- and over-propionylation affecting downstream quantification ^10^. This was apparent when we included C-terminal of K as a potential site of Trypsin cleavage in the ‘Trypsin + Prop’ condition, where it is assumed that all lysines are propionylated and hence unavailable for Trypsin cleavage. Inclusion of K in cleavage specificity increased the number of identified peptides by ~2.2 fold (~2.4 fold considering peptides with 0 missed cleavages) compared to when only C-terminal of R Trypsin cleavage is included, suggesting that many lysines are not propionylated and therefore available for cleavage (Figure S1A). For this reason and those described in the previous section, we performed an assessment of TMT as an alternative labeling strategy to propionylation. Although the use of TMT for histone PTM analysis has been suggested elsewhere ^24^ and demonstrated for histone H3 middle-down proteomics analysis ^25^ , as well as for quantification of DNA damage-associated changes to histones following chromatin cross-linking in yeast ^26^, there has been no direct comparison of TMT to propionylation or other chemical derivatizing agents such as TMA ^9^, phenyl isocyanate (PIC) ^24^, or d^6^-acetic anhydride + PIC ^23^ for labeling histone-peptides. Here, we compared the Arg-C Ultra and r-Chymotrypsin (1:10) generated peptides labeled with TMT to the established method of ‘Trypsin + Prop’, as these alternatives facilitate rapid sample processing with ≤ 2 h digestion times and only one round of labeling post-digestion. We first assessed the key metrics described in Figure 2 against ‘Trypsin + Prop’, labeling efficiency by site count and intensity, and then determined the number of informative peptides that could be used for quantification i.e., fully labeled peptides with ≤ 1 missed cleavage and no artifacts (e.g., Methionine oxidation).

The median peptide CVs of TMT-labeled Arg-C Ultra and r-Chymotrypsin peptides were lower than ‘Trypsin + Prop’ peptides (Figure S1A). Median lengths of fully cleaved and TMT-labeled peptides generated by Arg-C Ultra and r-Chymotrypsin were similar to peptides from “Trypsin + Prop” (~10–15 amino acids; Figure S1B). TMT-labeling yielded ~1.2-fold more fully cleaved peptides with Arg-C Ultra than the established ‘Trypsin + Prop’ approach (217 vs 179; Figure S1C); ‘Arg-C Ultra + Prop’ yielded 1.17-fold more completely-cleaved peptides than TMT-labeling (254 vs 217; Figure 2C and Supplementary Figure S2C); TMT-labeling yielded >15-fold more completely-cleaved peptides than propionylation when treated with r-Chymotrypsin (121 vs 7). Except for H4, histone sequence coverage deteriorated with Arg-C Ultra upon TMT-labeling (e.g., H3 decreased from 80% to 59%), while histone sequence coverage remained similar or improved when r-Chymotrypsin peptides were TMT-labeled, except H2A.X (48% to 0%; Figure S1D). Artifact rates such as methionine oxidation and dehydration on S/E/T/Q were 2% and 4.5%, respectively, for ‘Trypsin + Prop’. This dehydration decreased to 0.6% and oxidation increased to 7.5% when Arg-C Ultra peptides were labeled with TMT, while both artifacts increased to 15.4% and 12.9%, respectively, in TMT-labeled peptides from r-Chymotrypsin digest (Figure S1E).

### Labeling efficiency of propionic anhydride vs TMT

**Figure 4.**
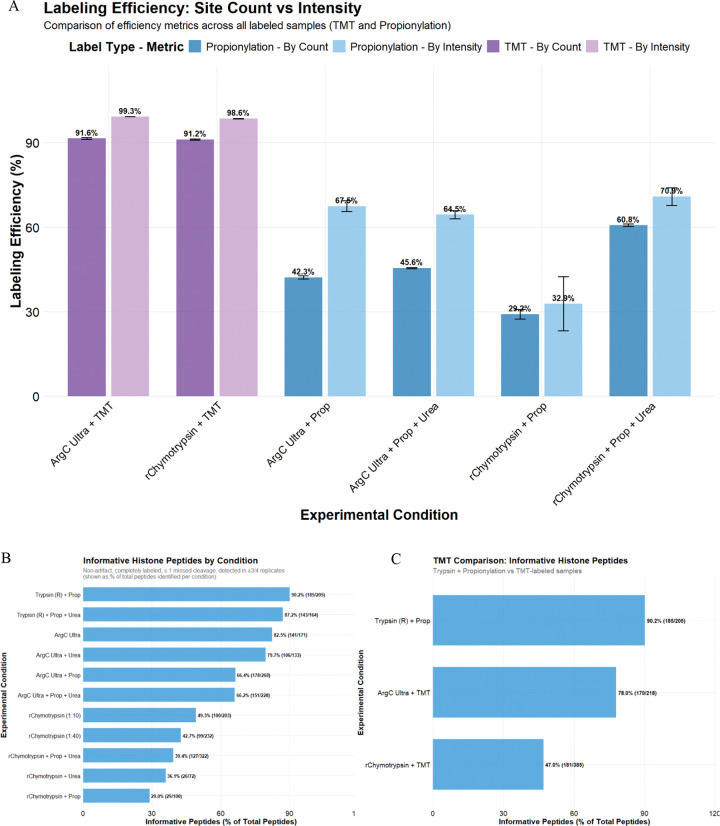
Comparison of internal lysine (K) labeling efficiency and informative peptide coverage across enzyme digestion strategies for histone PTM analysis. (A) Labeling efficiency metrics comparing propionylation and TMT-labeling approaches across all experimental conditions. TMT-labeling efficiency by intensity was >98% in Arg-C Ultra and r-Chymotrypsin (1:10) digested samples, compared to propionylation efficiency by intensity of ~68% in Arg-C Ultra and ~33% in r-Chymotrypsin. Efficiency was calculated using two methods: by site count (darker bars) showing the percentage of sites that were successfully labeled out of all theoretically available sites for labeling, and by intensity (lighter bars) representing the sum of labeled peptide intensities divided by total peptide intensity. Purple bars indicate TMT labeling efficiency, while blue bars show propionylation efficiency. Error bars represent standard deviation across *n* = 4 digestion replicates. (B) Percentage of IHP identified across different enzyme digestion conditions where IHP are defined as peptides containing completely labeled N-termini and lysine residues, allowing for precise PTM quantitation. Searching the Trypsin-digested samples with R cleavage specificity overestimates labeling efficiency, as any unlabeled K residues would result in cleavage at the lysine C-terminus. These peptides are not included in the search results, except in the case of a missed cleavage event. This is evident in Figure 4B, where “Trypsin + Prop” resulted in the highest IHP (~90%), with unlabeled Arg-C Ultra following close behind (~83%). Arg-C Ultra digestion of propionylated histones decreased this IHP proportion to ~66%, but the number of IHP (178) was comparable to “Trypsin + Prop”. (C) TMT-labeling improved the number of IHP generated by Arg-C Ultra (170 vs 141) and r-Chymotrypsin (181 vs 100), which was comparable to “Trypsin + Prop” (185). IHP values represent the percentage of total peptides that are informative for histone PTM analysis. Numbers in parentheses indicate IHP out of total peptides identified.

Trypsin cleavage at non-propionylated K resulted in the loss of ~58% of peptides (considering fully cleaved peptides) with ‘Trypsin + Prop’, which also prohibits the direct assessment of propionylation efficiency for Trypsin-digested samples (Figure S1F). However, we could directly assess propionylation efficiency for Arg-C Ultra and r-Chymotrypsin digests, where only one round of propionylation was performed post-digestion (Figure 4A). Considering only internal K as potential sites of propionylation (all peptide N-termini were considered propionylated, hence a static/fixed modification), propionylation efficiency calculated by site count (29–61%) was lower overall compared to estimation by intensity (33–71%). Efficiency of TMT-labeling was ~92% by site count and ~99% by intensity for peptides from Arg-C Ultra digest, and ~91% by site count and ~99% by intensity for peptides from r-Chymotrypsin digest.

Histone peptides that are reproducibly generated and identified with minimal variability are the best candidates for precise quantification and highly confident comparative analysis, which we termed informative histone peptides (IHP). We defined these as peptides with ≤ 1 missed cleavage, identified in 3 out of 4 replicates, and lacking common artifacts like methionine oxidation or dehydration (Figure 4B). The effect of labeling and its efficiency was significant on IHP, with unlabeled samples outperforming propionylated samples overall when considering the proportion of IHP among total identified peptides. Although labeling (TMT and propionylation) and the presence of urea generally increased the total number of identified peptides, they slightly decreased the proportion of IHP. As mentioned above, since we substantially underestimate the total peptides in ‘Trypsin + Prop’ samples due to partial propionylation of K resulting in Trypsin-cleavage at these unlabeled K residues, we substantially overestimate the proportion of IHP (87–90%) in ‘Trypsin + Prop’ conditions. Arg-C Ultra digests provide a high proportion of IHP (~80%) and r-Chymotrypsin digests yield ~40–50% IHP, which decreases to ~30–35% in the presence of urea or propionylation. TMT-labeling improved the number of IHP for both Arg-C Ultra and r-Chymotrypsin digestions (141 vs 170 and 100 vs 181, respectively), which was similar to IHP from ‘Trypsin + Prop’ condition (185) and ‘Arg-C Ultra + Prop’ (178).

To further explore the impact of suboptimal labeling on the number of IHP, we compared our search results from Trypsin with cleavage specificity set to R (for fully labeled K residues) to a separate search with cleavage specificity set to KR (for partially/unlabeled K residues; Supplementary Figure S1F). The number of fully labeled and properly cleaved peptides in ‘Trypsin + Prop’ (R) (179) constitutes ~33% of the total number of peptides obtained from this method (sum of all peptides from ‘Trypsin + Prop’ (KR); Supplementary Figure S1F).

### Histone PTM identification

**Figure 5.**
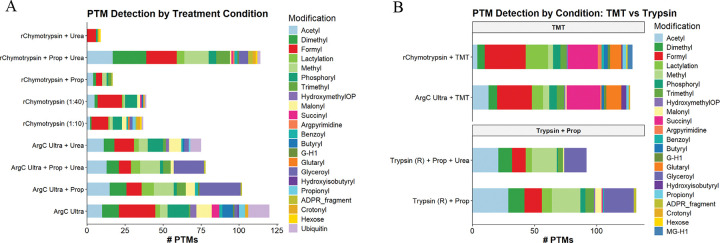
Diversity and abundance of histone post-translational modifications detected across enzyme digestion and chemical labeling strategies. (A) Total number and distribution of PTM types identified across different experimental conditions. Conditions include Arg-C Ultra, Trypsin (considering cleavage only after R), and r-Chymotrypsin digestion combined with various chemical treatments: propionylation alone (Prop), urea denaturation alone (Urea), or both treatments combined (Prop + Urea). For r-Chymotrypsin, enzyme-to-substrate ratios of 1:10 and 1:40 were also tested without chemical derivatization. Each stacked bar represents the cumulative count of unique PTM sites detected, with colors corresponding to specific modification types as indicated in the legend. (B) Comparison of identified PTMs between TMT-labeled peptides from Arg-C Ultra and r-Chymotrypsin digestions and propionylated Tryptic peptides. PTMs detected include common histone modifications (acetylation, methylation states, dimethylation, trimethylation, formylation, phosphorylation, and ubiquitinylation) as well as less abundant modifications (lactylation, succinylation, malonylation, benzoylation, butyrylation, propionylation, glyceroylation, glutarylation, hydroxyisobutyrylation, hydroxymethyl-OP, argpyrimidine, advanced glycation end products including G-H1 and MG-H1, crotonylation, hexose modifications, and ADPR fragments). Ubiquitin remnants are identified in unlabeled histone peptides, specifically those digested with Arg-C Ultra, whereas propionylation identifies glyceroylation sites not detected in labeled samples. TMT labeling improves identification of succinylated and glutarylated peptides. Endogenous propionylation is also discernible in non-propionylated peptides generated by Arg-C Ultra and r-Chymotrypsin.

Systematic evaluation of PTM detection across the different experimental conditions revealed substantial differences in both the total number and diversity of modifications identified (Figure 5). Arg-C Ultra and r-Chymotrypsin digestion achieved high PTM coverage, detecting up to ~120 unique modifications and over 15 distinct PTM types, including acetylation, methylation (mono-, di-, and tri-methyl states), ubiquitination (GG and RGG depending on Arg-C Ultra or r-Chymotrypsin digestion, respectively), and acylations such as crotonylation, lactylation, succinylation, and malonylation. Arg-C Ultra digestion resulted in identification of high numbers of PTMs under most conditions, while ‘r-Chymotrypsin + Prop + Urea’ outperformed all other r-Chymotrypsin conditions substantially. However, coverage of the most prevalent modifications (including acetylation, methylation, and phosphorylation). was achieved in all conditions. Endogenous propionylation and butyrylation were identified in unpropionylated Arg-C Ultra and r-Chymotrypsin digested peptides, which cannot be distinguished from peptides labeled with propionyl +/− mono-methylation when propionic anhydride is used as a derivatizing agent. TMT-labeled peptides from Arg-C Ultra and r-Chymotrypsin digestion achieved comparable number of PTMs as the conventional ‘Trypsin + Prop’ methods (~120 PTMs), and enhanced identification of specific modification-types, including a pronounced enrichment of negatively charged acylation marks such as succinylation and glutarylation (Figure 5B). The TMT-labeling approach with Arg-C Ultra and r-Chymotrypsin detected a greater diversity of PTM classes than propionylation methods while offering superior labeling efficiency. Labeling with propionic anhydride enabled the identification of glyceroyl sites in samples digested with all three enzymes under study, which were minimally detected in TMT-labeled or unlabeled samples, whereas Arg-C Ultra +/− urea increased the identification of ubiquitination remnants that were not readily detected in labeled samples. As the N-terminal amino acid of the GG remnant normally undergoes modification with amine-reactive labels, the charge state is neutralized with propionylation; in combination with peptide N-terminal modification, the overall charge state of a propionylated vs unpropionylated peptide containing a GG remnant would be ~ +1 compared to ~ +3. Samples digested with Arg-C Ultra without propionylation are more likely to be selected for fragmentation (≥ +2 charge) and to exhibit increased ionization efficiency for a subset of PTMs that normally suffer from charge neutralization during propionylation. These findings indicate that the choice of protease and labeling strategy profoundly influences both the depth and composition of the identified histone-PTM landscape.

### Enhanced identification of histone succinylation and glutarylation with TMT

Lysine succinylation and glutarylation are acidic acyl modifications that reverse the charge of modified residues from +1 to −1, destabilizing nucleosome structure and promoting chromatin accessibility ^27,28^. Both modifications are enriched at promoters of active genes, regulated by acetyltransferases (KAT2A, p300/CBP) and sirtuins (SIRT5, SIRT7), and linked to metabolic state through their acyl-CoA donors ^29–31^. Dysregulation of these modifications has been implicated in cancer, control of protein-protein and DNA-protein interactions, and defective DNA repair ^14^, yet their comprehensive profiling has been limited by suppressed ionization of acidic peptides in mass spectrometry-based workflows. A striking and serendipitous finding from our systematic evaluation was the dramatically enhanced identification of succinyl-lysine and glutaryl-lysine sites when peptides were TMT-labeled (Figure 5B). While our current work and the recent study by Ryzhaya et al.^9^ demonstrate that chemical derivatization at the peptide level improves histone PTM analysis, our data reveal that TMT provides unique advantages for identifying acidic acylations that extend beyond the chromatographic improvements conferred by increased hydrophobicity alone. The enhanced detection of these negatively charged PTMs represents a fundamentally different capability rather than simply an incremental improvement, suggesting that TMT and TMA-based approaches may be complementary depending on the PTM-classes of interest.

Succinylation as a histone PTM is thought to occur less frequently than acetylation and methylation ^13,27^. While the biological significance of histone succinylation is still being explored, a recent study found that an increase in global histone succinylation is associated with longevity ^32^, thus there is a growing interest in this PTM. In the current study, we found that succinylation was often identified on a first or second position K, adjacent K residues in the middle of a peptide, or near the peptide-C-terminus (See Supplementary Table S1), which can be challenging regions of the peptide for confident PTM localization. For histones digested with r-Chymotrypsin and peptides labeled with TMT, most succinylation sites were identified in H1.4 (7 sites), H2B (4 sites), and H4 (3 sites), and in some cases these sites were identified in regions of the protein not covered when histones were digested with Arg-C Ultra or ‘Trypsin + Prop’ approach (Supplementary Figures S3 and S4).

To explain this phenomenon of increased succinyl-K identification in TMT-labeled peptides, we explored the chemical and structural properties of TMT vs propionyl-group and investigated how it relates to MS analysis (as shown in Figure 3). The proton sequestration that occurs at the tertiary amine of TMT adds a positive charge at the peptide N-terminus and generally increases *b-*ion series (Figure 3). In our dataset, this led to an increase in fragment ions flanking a PTM site compared to derivatization with propionic anhydride (Supplementary Figure S3 and S4). Another important alteration that occurs with the addition of succinylation is the change in charge state from +1 (unmodified K) to −1 (succinyl-K). Succinylation introduces a carboxylic acid moiety that normally impairs positive-mode electrospray ionization via charge state reduction. Although propionylation increases the hydrophobicity of peptides, it neutralizes the charge of K residues and peptide N-termini, whereas the tertiary amine within the TMT reporter region provides a compensating protonation site, rescuing ionization of succinylated peptides. This charge state compensation was also found to increase the identification of another negatively charged acylation, glutarylation (114.031694 Da) ^33,34^. TMT’s superior labeling efficiency compared to propionylation (Figure 4) further enhances this effect.

Others have used more complicated techniques to study these negatively charged modifications in recombinant histone proteins using synthesized succinyl-K and glutaryl-K thioester derivatives ^14^. Thus, TMT-labeling offers a practical alternative that can be performed with relative ease to study negatively charged PTMs in biological samples that would normally pose a significant technical challenge. In summary, the dramatic improvement in identification of succinylation (50 sites) and glutarylation (27 sites) on histones with TMT-derivatization is likely attributable to improved ionization/fragmentation efficiency and subsequently enhanced PTM-site scoring. Representative annotated MS/MS spectra demonstrating unambiguous localization of succinyl-lysine sites are provided in Supplementary Figures S3 and S4, showing diagnostic *b*- and *y*-ion series flanking the modified residues. Our data suggest that succinylation of histone K residues may be more abundant than previously thought, revealing a PTM signature in HEK293T cells that was otherwise largely undetected by other methods investigated in the current study and well above what has been reported by others using MS-based methods ^13,27,32^.

### Systematic Evaluation of Protease and Labeling Strategies: Advantages of a Multi-Protease Workflow

Our systematic comparison of proteases and labeling conditions across 40 samples provides several key insights that extend beyond recent reports of individual protease performance. While Ryzhaya et al. ^9^ recently demonstrated that the use of Arg-C Ultra simplifies histone preparation compared to conventional Arg-C and that peptide-level derivatization with TMA enhances chromatographic separation of positional isomers, our comprehensive evaluation reveals additional layers of complexity in method selection and highlights the value of complementary multi-protease strategies.

First, our data demonstrates that different proteases and labeling strategies are necessary to optimally sequence various histones and map different PTM classes. Arg-C Ultra excels at generating properly cleaved peptides with minimal missed cleavages (>99% specificity for properly cleaved peptides in TMT-labeled samples), confirming recent reports of its improved performance over conventional Arg-C. However, our systematic evaluation revealed that r-Chymotrypsin provides critical orthogonal coverage for regions poorly represented by arginine-specific cleavage. Specifically, r-Chymotrypsin achieved 95% sequence coverage of H4, 80% coverage of H2A.Z, and 36% coverage of H2A1A, compared to minimal or no detection of these regions with Arg-C Ultra and ‘Trypsin + Prop’ (Figure S1D). This complementary coverage is essential for comprehensive histone variant characterization and detection of PTMs in linker histone H1 variants, which showed 28–47% coverage with r-Chymotrypsin compared to near-zero coverage with other protease strategies.

Second, our comparison of TMT and propionylation-based derivatization revealed that these approaches have distinct strengths beyond simple differences in labeling efficiency. While both TMT-labeling and propionylation increase peptide hydrophobicity and improve chromatographic retention, TMT’s single-step labeling efficiency substantially reduces workflow complexity and improves reproducibility compared to multi-round propionylation. More importantly, we discovered that TMT’s unique chemical structure – specifically, the tertiary amine within the reporter region – provides charge compensation that rescues ionization of negatively charged acylations. This resulted in a cumulative detection of 50 succinylation sites and 27 glutarylation sites using Arg-C Ultra and r-Chymotrypsin digestion, representing a dramatic increase over the propionylation-based approaches tested in this study. This finding suggests that the prevalence of acidic histone acylations may have been systematically underestimated using conventional derivatization methods, revealing what we term the ‘dark epigenome’ of negatively charged PTMs.

Third, our data supports a decision framework for method selection based on research objectives. For rapid screening of histone PTMs where acidic acylations are of interest, RIPUP using Arg-C Ultra and TMT-labeling provides high labeling-efficiency, excellent PTM coverage, and enhanced detection of succinylation and glutarylation within a ~3-hour workflow. When complementary coverage of H2A variants and linker histones is required, addition of r-Chymotrypsin digestion provides access to regions not covered by arginine-specific cleavage. For studies focused on positional isomer quantification where TMA’s higher hydrophobicity may provide advantages, the TMA-based workflow described by Ryzhaya et al. represents a viable alternative. Importantly, both approaches substantially reduce the sample preparation needed for conventional ‘Trypsin + Prop’ workflows (~72 hours), with RIPUP offering the additional advantage of TMT’s multiplexing capabilities for future comparative studies.

Finally, our systematic evaluation highlights the importance of matching analytical conditions to specific peptide properties. Short, hydrophilic peptides (< 11 amino acids, ≥ 2 lysines) benefited substantially from chemical derivatization regardless of reagent choice, with 170–177 IHP identified in TMT-labeled and propionylated conditions and only 101–120 IHP in unlabeled samples. In contrast, longer peptides (> 15 amino acids) were better detected without derivatization or with minimal labeling, suggesting that no single approach captures the complete histone PTM landscape. This underscores the value of our multi-protease, multi-condition systematic evaluation in defining optimal strategies for different analytical scenarios.

### RIPUP of hippocampal sections

**Figure 6.**
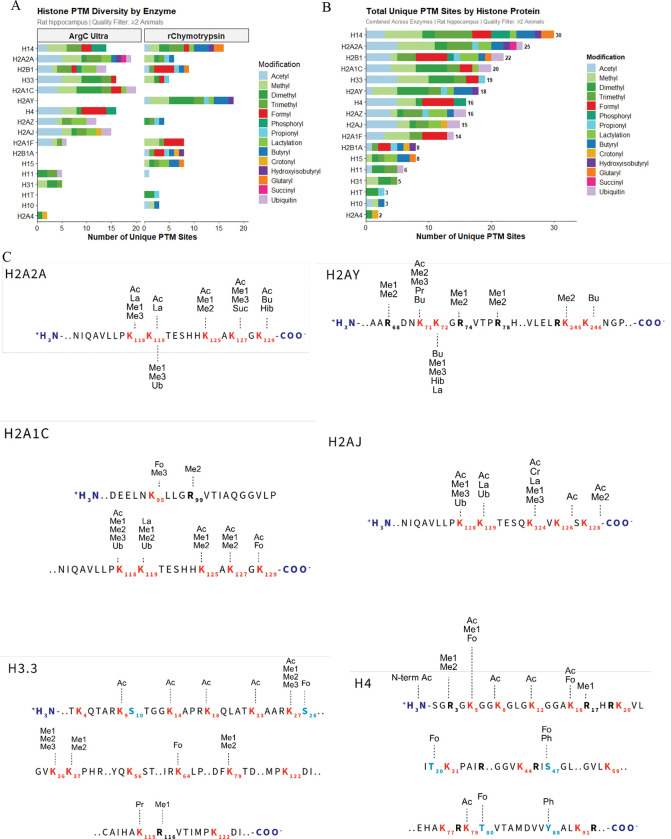
Comprehensive histone post-translational modification landscape in rat hippocampus. **(A)** Histone PTM diversity detected by each enzyme digestion strategy. Stacked bar plots show the number of unique PTM sites identified per histone protein using Arg-C Ultra (left) or r-Chymotrypsin (right). Colors indicate modification type. Quality filter: PTMs detected in ≥2 animals. **(B)** Total unique PTM sites per histone protein when combining both enzyme strategies, demonstrating the complementary coverage achieved through multi-enzyme analysis. Numbers indicate total unique sites per protein. H14 (linker histone H1.4) and H2A2A showed the greatest PTM diversity with 30 and 25 unique sites, respectively. **(C)** Site-specific PTM maps for selected histone variants. Peptide sequences show detected modification sites with lysine (K), arginine (R), serine (S), and threonine (T) residues highlighted in red, black, and cyan (S and T), respectively. Subscript numbers indicate canonical residue positions. Modifications detected at each site are listed above or below the sequence. Abbreviations: Ac, acetylation; Me1/Me2/Me3, mono-/di-/trimethylation; Fo, formylation; Ph, phosphorylation; Ub, ubiquitination; La, lactylation; Bu, butyrylation; Cr, crotonylation; Hib, 2-hydroxyisobutyrylation; Pr, propionylation; Suc, succinylation. N-term, N-terminal modification. Data represent combined analysis from male rat hippocampi (*n* = 5). Figure 6C created in https://BioRender.com.

As a proof-of-principle experiment to determine whether our rapid protocol could detect desirable histone peptides and their PTMs *in vivo*, we extracted histones from rat hippocampal sections and performed RIPUP, using separate digestions with Arg-C Ultra and r-Chymotrypsin. The total sample preparation time (digestion and sample loading) was ~ 3 h, and we identified 212 and 189 peptides with ≤ 1 missed cleavage with Arg-C Ultra (1:10) and r-Chymotrypsin (1:10), respectively (Supplementary Figure S5A). Median CVs were ~7% for both Arg-C Ultra and r-Chymotrypsin digests (Supplementary Figure S5B). The total number of PTMs detected using both enzymes for digestion totaled 231, and RIPUP detected all major classes of PTMs including acetylation, mono-, di-, and tri-methylation, phosphorylation, and ubiquitination (Figure 6A and B). Less common classes of histone PTMs were also detected, including endogenous propionylation and butyrylation, which normally escape detection when derivatized with propionic anhydride (refer to methods section for details on isobaric mass shifts).

Analysis of selected core and variant histone sequences confirmed that desirable regions of sequence coverage such as the highly modified histone N-terminal tails of H3 and H4, and ubiquitination sites K118/K119 on H2A were captured by our RIPUP method (Figure 6C). We also identified N-terminal acetylation and R3 mono- and di-methylation on H4, which are implicated in the regulation of gene transcription and chromatin dynamics via PTM crosstalk between R3 methylation and K acetylation states at the N-terminal tail ^35–37^. Multiple peptidoforms corresponding to the H3 peptide aa 9–17 and the H4 peptide aa 4–17 were identified, enabling detection of various combinatorial acetylation patterns that are known to alter chromatin structure ^38,39^ and have biological implications for various diseases ^6,40,41^. (See Supplementary file 3 for the complete list of modified peptides identified by Arg-C Ultra and r-Chymotrypsin.)

The critical region of interest on histone H3 (aa 27–40) as well as mono- and di-methylation on K79, were also detected in Arg-C Ultra-digested samples. The methylation patterns on H3 K27, K36, and K37 are known to function as transcriptional activators or repressors via Polycomb silencing ^42–44^, thus the rapid screening of methylation at these sites is crucial for interpreting gene expression anomalies in diseases such as cancer ^45^ and could facilitate streamlined development of targeted therapeutics.

RIPUP detected extensive PTM diversity on linker histone H1 variants. H1.4 contained 16 unique modification sites spanning residues 75–102, including succinylation at K75 and K90, crotonylation at K81, lactylation at K97, and arginine mono- and di-methylation at R79. Similarly, we identified succinylation (K74) and crotonylation (K74, K80) in H1.5, while H1.9 displayed a complex modification profile at K96 (me3, pr, cr, bu) and K106 (me2, me3, cr, la, bu), as well as ubiquitination (RGG remnant identified by r-Chymotrypsin) at K97. These findings underscore the utility of underivatized peptide analysis for detecting linker histone PTMs that may be obscured by propionylation.

Core histone macro-H2A.1 exhibited arginine methylation at R69, R75, and R79 (mono- and di-methyl states), along with extensive lysine modifications at K72 (ac, me2, me3, pr, bu) and K73 (me1, me3, cr, la, bu). H2B K59 emerged as a highly modified site, with seven distinct modification types detected at this single residue: acetylation, butyrylation, propionylation, lactylation, trimethylation, 2-hydroxyisobutyrylation, and ubiquitination (RGG remnant). Glutarylation was also detected on H2A K119, which demonstrates the benefit of the retained positive charge on the peptide N-terminus in underivatized peptides for detecting negatively charged PTMs.

Interestingly, we found that formylation of K, S, T, and Y residues were a prominent feature in histones extracted from HEK293T and rat hippocampal sections; an observation also made by the authors of the HiP-Frag workflow across multiple cell lines ^15^. Although formylation can arise as an artifact of sample preparation when incubating in high concentrations of formic acid ^46^, this was not a feature of our workflow, and others have shown that formylation is an abundant and biologically important histone PTM on K ^47–49^. This points to formylation as an epigenetic mark that should garner attention in future studies to fully establish its role in chromatin structural dynamics and epigenetic regulation of gene expression.

## Discussion

### Computational Stringency Enables Large-Scale PTM Discovery: Positioning RIPUP Within Recent Advances

The identification and validation of novel histone PTMs represents a significant analytical challenge, particularly when PTMs occur at low abundance or are systematically under-detected by conventional methods. Historically, the gold standard for PTM confirmation has been validation with synthetic peptides; however, this approach becomes impractical when investigating dozens of potential novel marks across multiple histone proteins and variants. Recent work by Vai et al. (2025) addressed this challenge by developing HiP-Frag, a computational workflow that integrates detailed mass offset search, open search, and closed search strategies to enable confident identification of novel histone PTMs through rigorous computational filtering rather than comprehensive synthetic validation ^15^. Using this approach, they identified 60 previously unreported marks on core histones and 13 on linker histones across nine cancer cell lines and breast cancer tissue samples.

Our study builds upon and extends this computational framework by applying HiP-Frag principles to a systematic comparison of protease and labeling strategies. While Vai et al.^15^ demonstrated the power of unrestrictive search for PTM discovery, our work reveals that specific labeling strategies – in this case, TMT vs propionylation – can dramatically influence which PTM classes are detected. The dramatic enhancement of succinylation and glutarylation detection with TMT compared to all propionylation-based approaches in our study provides a mechanistic explanation for why certain acidic acylations may have been underreported in the literature; conventional propionylation neutralizes positive charges on K residues without providing charge compensation for negatively charged modifications, resulting in poor ionization efficiency in positive-mode electrospray ionization.

Importantly, our findings suggest that the TMT and propionylation workflows may reveal complementary PTM landscapes rather than competing approaches. While both Vai et al.’s work and our systematic evaluation demonstrate that computational rigor can enable confident PTM identification at scale, TMT’s charge compensation effect explains why certain modification classes may be preferentially detected depending on the labeling strategy employed. This has important implications for the design of future histone PTM profiling studies. Researchers interested in acidic acylations (succinylation, glutarylation, malonylation) should consider TMT-based workflows, while those focused on positional isomer separation may benefit from TMA-based approaches as demonstrated by Ryzhaya et al. (2025). The orthogonal nature of these methods underscores the value of multi-strategy approaches for comprehensive PTM characterization.

Furthermore, the application of our workflow to rat hippocampal tissue demonstrated that computationally driven PTM discovery is not limited to cultured cells but extends to complex biological samples, consistent with Vai et al.’s analysis of breast cancer tissues. The detection of 231 unique PTM sites within a 3-hour workflow, including biologically critical PTMs such as H4 K5/K8/K12/K16 acetylation, H3 K27/K36/K37 methylation, and extensive linker histone modifications, validates that RIPUP can deliver rapid, comprehensive PTM profiling in scenarios where time-to-result is critical.

In our analysis, we applied 1% FDR at PSM and peptide levels, minimum detection in ≥3 digestion replicates (HEK293T) or ≥2 biological replicates (rat hippocampi), and CV filtering to ensure high-confidence assignments. While we acknowledge that synthetic peptide validation remains the gold-standard for PTM confirmation, our adoption of HiP-Frag’s stringent computational filters provides high confidence in our PTM assignments. The one synthetic peptide example provided by Vai et al. (H4K77 malonylation) to resolve isobaric PTM combinations demonstrates a pragmatic approach – targeted validation for ambiguous cases rather than comprehensive validation of all discoveries. Future studies could similarly prioritize synthetic validation of the most abundant or biologically relevant succinylation and glutarylation sites identified here, particularly those occurring at known regulatory residues.

### RIPUP: A Rapid Multi-Protease Workflow for Comprehensive Histone PTM Analysis

The RIPUP workflow represents more than simply a faster alternative to conventional histone PTM analysis; it introduces a strategic multi-protease approach that maximizes sequence coverage while minimizing sample preparation time. By combining Arg-C Ultra and r-Chymotrypsin digestions, RIPUP provides access to distinct regions of the histone proteome: Arg-C Ultra efficiently covers N-terminal tails and generates quantitative information for abundant PTM sites, while r-Chymotrypsin captures H2A variants (H2A.Z, H2A1A), linker histone H1 variants (H1.1-H1.5), and regions that are poorly represented by arginine-specific cleavage.

Using the HiP-Frag computational framework, we identified 50 succinylation sites and 27 glutarylation sites in HEK293T cells, representing a substantial expansion of the known histone acylation landscape. This finding parallels the recent discovery of 60 previously unreported core histone modifications using unrestrictive search strategies ^15^, and suggests that acidic acylations may have been under-detected using conventional propionylation-based workflows. While Vai et al. demonstrated that computational stringency enables confident novel PTM identification without synthetic validation, our observation that TMT specifically enhances acidic PTM detection through charge compensation provides a mechanistic explanation for this expanded coverage and suggests that TMT and propionylation-based approaches may reveal complementary PTM landscapes.

Our application of RIPUP to rat hippocampal tissue demonstrates the practical utility of this approach for biological discovery. Within a 3-hour sample preparation window, we identified 231 unique PTM sites across both enzymes, including all major PTM classes and numerous sites of known biological importance. Critical regions such as H3 K27/K36/K37 (Polycomb silencing and transcriptional regulation), H4 N-terminal tail acetylation patterns (chromatin structure and gene activation), and H2A K118/K119 ubiquitination (DNA damage response) were readily detected. Importantly, we also identified extensive PTM diversity on linker histone H1.4 (16 unique modification sites spanning residues 75–102), which would have been largely missed using Arg-C Ultra or Trypsin alone.

The rapid turnaround time of RIPUP is particularly advantageous for studies requiring quick screening of histone PTM landscapes prior to more detailed quantitative analysis. For example, in the context of drug discovery targeting epigenetic modifiers, RIPUP could enable same-day assessment of compound effects on histone PTMs to accelerate the path to translation. Similarly, in clinical research contexts where rapid molecular profiling is essential (e.g., intraoperative tumor assessment, acute response studies), the 3-hour workflow makes histone PTM analysis feasible within operationally relevant timeframes.

RIPUP’s dual-protease strategy provides built-in orthogonal validation. PTMs detected by both Arg-C Ultra and r-Chymotrypsin (when sequence coverage overlaps) provide increased confidence in site assignment (Supplementary Figures S2-S4), while protease-specific detections expand the accessible PTM landscape. This approach aligns with emerging best practices in PTM proteomics emphasizing the value of complementary digestion strategies for improving coverage and localization confidence.

### Limitations

There are a few methodological considerations that should be noted when interpreting these results. Systematic enzyme and labeling comparisons were performed using a single cell line (HEK293T), and performance metrics may vary with different sample types, histone abundance, or extraction protocols, though we have demonstrated broader applicability through hippocampal tissue analysis. While the succinylation and glutarylation enhancement with TMT was dramatic in HEK293T cells, validation of this phenomenon across additional cell types or primary tissues would strengthen these findings. Second, despite employing complementary digestion strategies, sequence coverage gaps persist, particularly within histone globular domains; the reported PTM landscape reflects only those regions accessible to the enzymes under study. Alternative digestion strategies may be implemented to improve sequence coverage on a user-dependent basis by modifying the RIPUP protocol, as complementary digestion is an established method of improving sequence coverage to identify target regions of interest or to improve PTM identification and site localization ^50–52^.

PTM identification is somewhat constrained by the variable modification search space and mass offsets defined in the HiP-Frag workflow. Novel or unanticipated modifications outside this predefined list would not be detected, and the expanded search space inherent to histone PTM analysis increases the risk of false positives despite FDR correction. Additionally, isobaric mass or small delta mass ambiguities, such as trimethylation (+42.047 Da) versus acetylation (+42.011 Da), require high mass accuracy for confident discrimination, and some site assignments may remain probabilistic for peptides with multiple closely spaced modifiable residues, depending on the instrument and LC configuration. However, the RIPUP protocol is compatible with other bioinformatic pipelines such as open-search for PTM discovery, which would facilitate the identification of novel PTMs. Thus, these constraints do not exist within the protocol itself but are encountered in downstream analysis.

Following the approach of Vai et al. ^15^, we relied on stringent computational filtering rather than synthetic peptide validation for the majority of identified PTMs. While synthetic validation remains the gold standard for PTM confirmation, the computational rigor of the HiP-Frag workflow, including FDR control, CV and artifact filtering at the peptidoform level, and minimum detection thresholds, provides high confidence in PTM assignments. Nonetheless, future studies could prioritize synthetic validation of the most abundant or biologically relevant acidic acylation sites identified here.

Regarding labeling strategies, propionylation efficiency was not validated against synthetic peptide standards, and the reported efficiencies represent relative estimates derived from database searching. For TMT-labeled samples, it is important to note that TMT was employed exclusively as a derivatization agent to enhance peptide hydrophobicity and ionization efficiency. The multiplexing and quantitative capabilities of TMT were not utilized in this study, and the enhanced detection of succinylation and glutarylation likely reflects improved ionization rather than absolute abundance differences. Finally, endogenous propionylation and butyrylation cannot be distinguished from chemical derivatization artifacts in propionylated samples, but the RIPUP workflow circumvents this limitation by omitting chemical derivatization.

## Conclusions

We present RIPUP, a rapid multi-protease workflow for comprehensive histone PTM analysis that reduces sample preparation from days to hours while expanding PTM coverage through strategic use of complementary proteases. Our systematic evaluation of Arg-C Ultra and r-Chymotrypsin across multiple experimental conditions (40 samples spanning 10 distinct conditions) provides a detailed roadmap for method optimization based on research objectives. We confirm recent reports that Arg-C Ultra achieves superior digestion specificity compared to conventional Arg-C and extend these findings by demonstrating that TMT labeling offers unique advantages beyond those of TMA derivatization. Specifically, we discovered that TMT’s tertiary amine provides charge compensation that rescues ionization of acidic acylations, revealing 50 succinylation and 27 glutarylation sites that represent a previously hidden ‘dark epigenome.’ This finding suggests that the biological prevalence of these negatively charged PTMs has been systematically underestimated using conventional propionylation-based workflows.

The RIPUP workflow’s dual-protease strategy combining Arg-C Ultra for efficient coverage of N-terminal tails with r-Chymotrypsin for access to H2A variants, linker histones, and aromatic-rich regions provides comprehensive sequence coverage that cannot be achieved with single-protease approaches. Application to rat hippocampal tissue confirmed detection of 231 unique PTM sites including biologically critical marks within a three-hour sample preparation window, demonstrating practical utility for time-sensitive biological discovery.

Our systematic comparison of proteases, labeling strategies, and experimental conditions provides a framework for method selection based on analytical goals. RIPUP with TMT labeling is optimal when acidic acylations are of interest, when multiplexing capabilities are desired, or when rapid turnaround is essential, TMA-based approaches may offer advantages for certain positional isomer separations, and complementary r-Chymotrypsin digestion should be included when comprehensive coverage of H2A variants and linker histones is required. Collectively, these methods expand the analytical toolkit for histone PTM research and should facilitate higher-throughput epigenetic studies where rapid, comprehensive PTM profiling is essential for accelerating therapeutic discovery and mechanistic understanding.

## Supplementary Material

Supplement 1

Supplement 2

Supplement 3

Supporting information

Supplementary information is available as follows; File 1, Figure S1: Trypsin and TMT-labeled HEK293T comparisons (CVs, peptide length distributions, missed cleavages, histone protein sequence coverage, artifact rates), and cleavage specificity (R vs KR) for trypsin groups. Figure S2: H1.4 sequence coverage and succinylation sites in r-Chymotrypsin + TMT and Arg-C Ultra + TMT samples. Figure S3: H4 sequence coverage and succinylation sites in r-Chymotrypsin + TMT samples and fragment spectra for RDNIQGITkPAIRRL and ARRGGVkRISGL peptides. Figure S4: H4 sequence coverage and succinylation sites in Arg-C Ultra + TMT samples and fragment spectra for DNIQGITkPAIR and DAVTYTEHAkR peptides. Figure S5: Unique peptidoforms and CVs of histones extracted from rat hippocampal sections that underwent the RIPUP protocol. File 2: SDS-PAGE image files. File 3: Peptide lists of modified peptides identified in Rat hippocampal sections using the RIPUP workflow (Arg-C Ultra and r-Chymotrypsin).

## Figures and Tables

**Table 1:** Protease digestion conditions for histones extracted from HEK293T cells.

Sample type	Protease	Denaturation	Derivatization (propionylation)	Digestion conditions: time (temperature)
HEK293	Trypsin (control) 1:10	+/−	+ (before and after digestion)	6 h (37 °C)
	Arg-C ultra 1:100	+/−	+/− (after digestion)	2 h (37 °C)
	r-Chymotrypsin 1:40	+/−	+/− (after digestion)	2 h (RT)

**Table 2:** Denaturing conditions for histones extracted from HEK293T cells.

Enzyme	Cleavage Specificity	Denaturant	Denaturant concentration
Arg-C Ultra	R; C-term	Urea	6 M (with 10 mM TCEP)
r-Chymotrypsin	Y, F, L, M; C-term	Urea	6 M (with 5 mM TCEP)
Trypsin	R, K (non-propionylated); C-term	Urea	2 M (with 5 mM TCEP)
Arg-C Ultra		None	(with 10 mM TCEP)
r-Chymotrypsin			(with 5 mM TCEP)
Trypsin			(with 5 mM TCEP)

## Data Availability

The MS raw data files, annotations, Sample and Data Relationship Format (SDRF-Proteomics)^53^, and FragPipe search have been deposited to the ProteomeXchange Consortium (http://proteomecentral.proteomexchange.org) ^54,55^ via the PRIDE partner repository ^56^ with the dataset identifier PXD073683. The custom R scripts used for data analysis are available at: https://github.com/NataliePTurner/Histone-RIPUP
