# Supplementary material for: Rapid Histone Post-Translational Modification Analysis Using Alternative Proteases and Tandem Mass Tags
Source: bioRxiv. 2026 May 30:2026.02.13.705817. Originally published 2026 Feb 15. Preprint. [Version 4] doi: 10.64898/2026.02.13.705817 (PMC12918826; doi:10.64898/2026.02.13.705817)
Supplement: Supplement 1 [file media-1.pdf]

Supporting Information for Publication

Rapid Histone Post-Translational Modification Analysis Using Alternative Proteases and Tandem Mass Tags

Natalie P. Turner [1], Sabyasachi Baboo [1], Patrick Garrett [1], Jolene K. Diedrich [1,2], Michal Bajo [3], Marisa Roberto [3], and John R. Yates III [1]*.

[1] The Scripps Research Institute, Department of Integrative and Structural Computational Biology, 10550 North Torrey Pines Rd, La Jolla, CA 92037, United States of America

[2] The Scripps Research Institute Multi-Omics Core Facility, 10550 North Torrey Pines Rd, La Jolla, CA 92037, United States of America

[3] The Scripps Research Institute, Department of Translational Medicine, 10550 North Torrey Pines Rd, La Jolla, CA 92037, United States of America

Table of Contents

SI Methods......S2

Histone extraction......S2

SDS-PAGE......S2

FragPipe Settings......S3

References......S3

Figure S1. Heatmap showing variant-specific proteotypic sequence coverage in each condition. S4

Figure S2. Comprehensive quality metrics comparing TMT derivatization and propionylation workflows for histone peptide analysis......S5

Figure S3. H1.4 sequence coverage and succinylation sites in r-Chymotrypsin + TMT and Arg-C Ultra + TMT samples......S7

Figure S4. Representative extracted ion chromatogram (XIC) and matching mass spectrum for the H1.4 peptide KSAGAAKR (+3 ion) in Arg-C + TMT labeled samples. S7

Figure S5A. H4 sequence coverage and succinylation sites in r-Chymotrypsin + TMT samples. S8

Figure S5B. H4 sequence coverage and succinylation sites in Arg-C Ultra + TMT samples......S8

Figure S6. Arg-C Ultra missed cleavage motif analysis from NAM-treated and untreated HEK293T histones......S9

Figure S7. r-Chymotrypsin missed cleavage motif analysis from NAM-treated and untreated HEK293T histones......S10

Figure S8. Peptidoforms, missed cleavages, and CV distributions of rat hippocampal histone peptides obtained with RIPUP. S11

Figure S9. SDS-PAGE protein profiles of histones extracted from HEK293T cells and rat hippocampal sections......S11

Table S1: FragPipe Detailed Mass Offsets S13

Table S2: Protease digestion conditions for histones extracted from HEK293T cells......S14

Table S3: Denaturing conditions for histones extracted from HEK293T cells......S14

Table S4: Peptide lists of modified peptides identified in rat hippocampal sections using the RIPUP workflow (Arg-C Ultra and r-Chymotrypsin) S15

Table S5: Per-Sample Reagent and Researcher Time Cost Comparison (5 µg Histone Peptide Input)......S27

SI Methods

Histone extraction

Histones were extracted from HEK293T cells using an established protocol as described by Sidoli *et al* (Sidoli et al., 2016) Briefly, frozen-thawed aliquots of HEK293T cells were pooled by transferring to a 50 mL falcon tube, and the tube topped with DPBS warmed to 37 °C. Cells were washed twice by centrifugation and the cell pellet was resuspended in chilled nuclear isolation buffer (NIB) with DTT and protease inhibitors (Pierce EDTA-free protease inhibitor cocktail, ThermoScientific). Cells were washed once in NIB, lysed with NIB containing 0.2% NP-40 alternative, and homogenized by gentle pipetting. The mixture was incubated on ice for 10 min and centrifuged to pellet nuclei. The nuclei pellet was washed three times with NIB by centrifugation to remove traces of detergent, and the supernatant was discarded after the final wash. For rat hippocampal sections, frozen samples were thawed on ice, washed once with chilled NIB, transferred to a 2 mL dounce homogenizer, and homogenized/lysed with 10 – 15 strokes of the fine pestle. All other nuclei isolation steps were the same as described for nuclei extraction from HEK293T cells.

Acid extraction of histones from the nuclei pellets was performed by resuspending nuclei in 0.2 M H_2SO_4 and incubating at 4 °C for 2-3 h with gentle rotation. Insoluble material was removed by dual rounds of centrifugation, and trichloroacetic acid (TCA; 100%) was added to the supernatant to a final concentration of 33%, vortexed to mix, and incubated on ice overnight to precipitate histones. Pelleting and washing of precipitated histones with acetone/HCl and 100% acetone were performed as previously described (Sidoli et al., 2016). The final histone pellets were resuspended in ddH_2O and centrifuged briefly to pellet insoluble material. The supernatant was transferred to a new tube and assessed for quality by SDS-PAGE and protein concentration by BCA assay (Pierce™ BCA Protein Assay, cat number 23227, Thermo Scientific™). For HEK293T samples, the sample was divided into aliquots of ~5 µg protein according to Tables S1 and S2, with four technical replicates in each condition, resulting in a total of 40 samples. For rat hippocampal sections, 2.5 µg of purified histones were used per protease (5 µg total per sample).

SDS-PAGE

Aliquots of 5 µL extracted histones were mixed with 4x LDS sample buffer (NuPAGE™, Catalog number NP0007, Invitrogen™) and 10x sample reducing agent (NuPAGE™, Catalog number NP0009, Invitrogen™), to achieve a 1x final concentration in the sample. Samples were reduced for 10 min at 70 °C and 450 rpm in a Thermomixer (ThermoScientific). A volume equivalent to 2 µg total protein of purified histone H2A standard (Sigma-Aldrich, Cat number H9250, Sigma-Aldrich) was prepared in the same way. A 5 µL aliquot of protein ladder (BLUEstain™ 2 Protein ladder, 5-245 kDa, Cat Number: P008-500, Goldbio), samples, and H2A standard were separated by gel electrophoresis on a 4-12% Bis-Tris mini protein gel, 1.0–1.5 mm (NuPAGE™, Cat number: NP0321BOX, Invitrogen™) for 50 min at 150 V. The protein gel was placed into a clean gel tray and incubated with Acquastain gel stain (Bulldog Bio) to visualize protein bands. Gels were imaged on a gel imager with 5 s exposure (Azure biosystems c600).

FragPipe Settings

All default variable modifications and mass offsets were enabled as per default pipeline settings, except methylation (+14.01565 Da) was added as a variable modification to non-propionylated samples (default setting +70.0419 Da, the combined mass of a propionyl group and methyl group), and the +70.0419 Da mass was retained as endogenous butyrylation. Propionylation on serine, threonine, and tyrosine was removed from the detailed mass offset list for non-propionylated samples. For TMT-labeled samples, the monoisotopic mass of the intact label (+229.162932 Da) was set as a static modification on peptide N-termini and as a variable modification on K, and all other PTM declarations were consistent with unlabeled samples. Finally, there were specific mass shifts considered for Gly-Gly (GG; +114.0429 Da) or Arg-Gly-Gly (RGG; +270.1441 Da) remnants corresponding to cleavage of ubiquitinylated K (Arg-C Ultra and r-Chymotrypsin cleaved ubiquitin, respectively. These fragments can also be labeled with propionyl or TMT at the free amine at the N-terminus, so +170.0691 Da (GG + Prop) and +343.2058 Da (GG + TMT) for Trypsin or Arg-C Ultra-digested samples, and +326.1703 Da (RGG + Prop) and +499.307 Da (RGG + TMT) for r-Chymotrypsin-digested samples were included. Label-free quantification (LFQ) and match-between-runs (MBR) were enabled for quantification. The propionyl and TMT masses were removed from labeled peptidoforms during data analysis processing to enable direct qualitative comparison of identified peptidoforms between labeled and unlabeled conditions.

References

Sidoli, S., Bhanu, N. V., Karch, K. R., Wang, X., & Garcia, B. A. (2016). Complete workflow for analysis of histone post-translational modifications using bottom-up mass spectrometry: From histone extraction to data analysis. *Journal of Visualized Experiments*, *2016*(111). https://doi.org/10.3791/54112

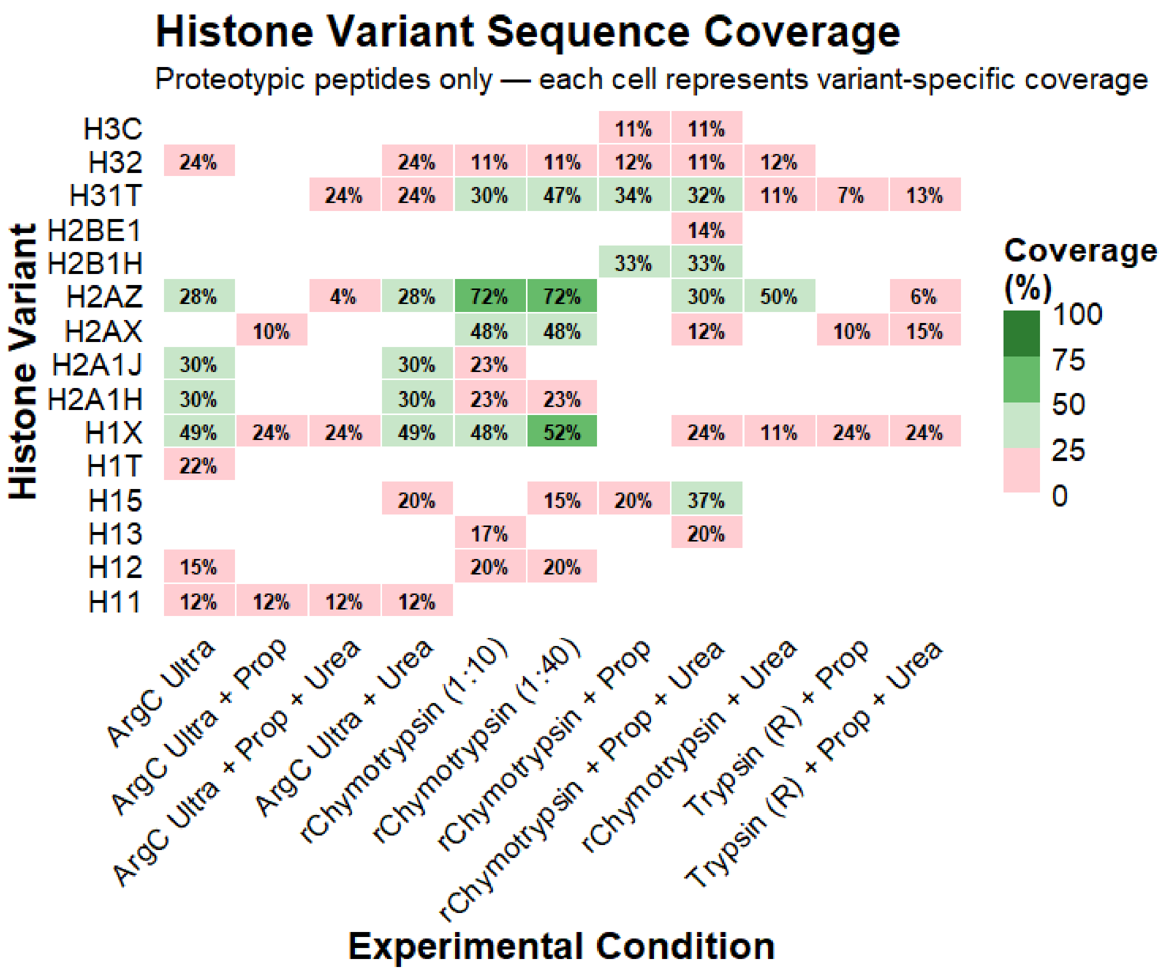

Figure S1. Heatmap showing variant-specific proteotypic sequence coverage in each condition.

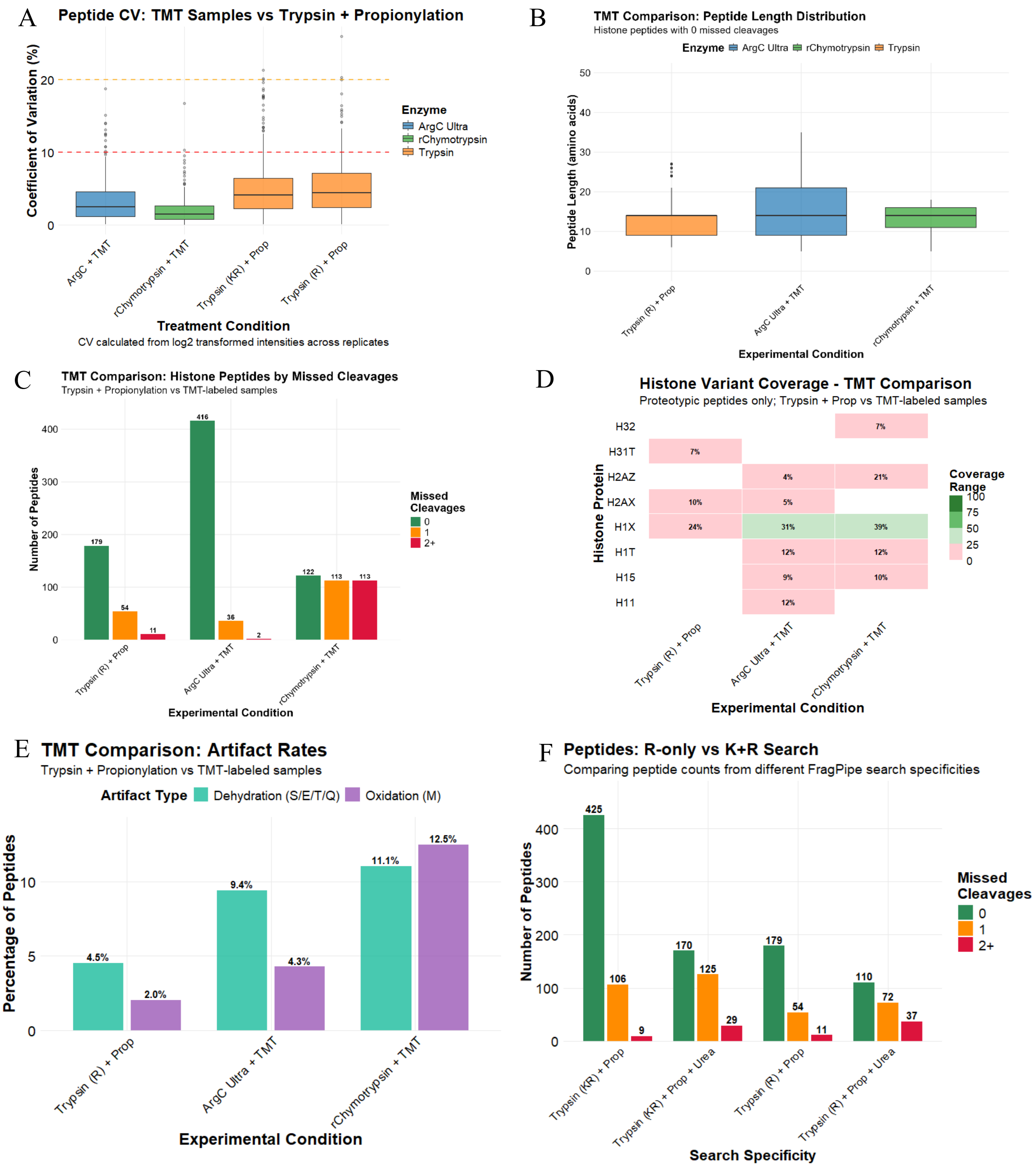

Figure S2. Comprehensive quality metrics comparing TMT derivatization and propionylation workflows for histone peptide analysis. **(A)** Coefficient of variation (CV) of peptide intensities across replicates. CVs were calculated from log2-transformed intensities. TMT-labeled samples (Arg-C Ultra, r-Chymotrypsin, Trypsin) demonstrate comparable or lower variability than Trypsin + Propionylation. Dashed lines indicate 10% and 20% CV thresholds. **(B)** Peptide length distribution for fully cleaved histone peptides (0 missed cleavages). Peptide length distributions are similar across Trypsin + prop and TMT-labeled samples (median ~15 amino acids). **(C)** Distribution of missed cleavages in identified histone peptides. **(D)** Histone protein sequence coverage heatmap using proteotypic peptides for histone variants. **(E)** Chemical artifact rates comparing derivatization methods.

Dehydration artifacts (S/E/T/Q) and methionine oxidation are shown as percentage of total peptides. **(F)** Effect of search enzyme specificity on peptide identifications. Comparison of R-only (fully labeled) versus K+R (partially labeled/unlabeled) search parameters across different sample preparation methods, stratified by missed cleavages.

H1.4 Succinylation sites: sequence coverage

Protease-specific site coverage

r-Chymotrypsin + TMT

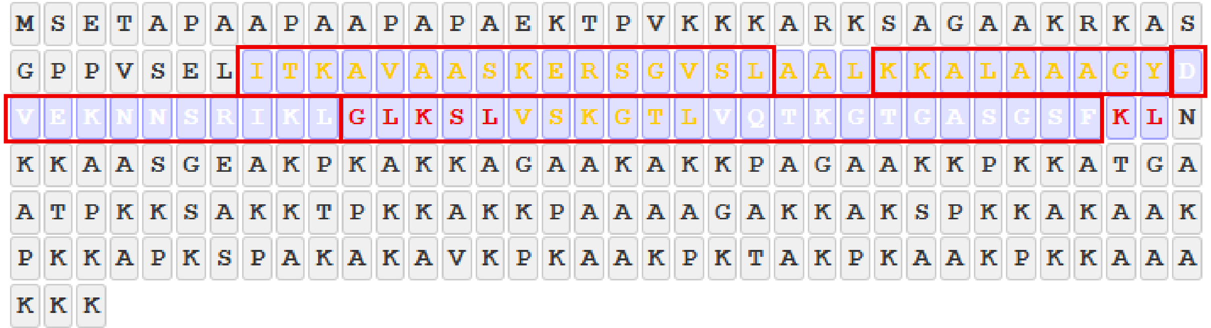

ITkAVAASKERSGVSL
ITKAVAASkERSGVSL
kKALAAAGY
KkALAAAGY
DVEkNNSRIKL
DVEKNNSRIkL
GLkSLVSKGTL
GLKSLVSkGTL; VSkGTL; VSkGTLVQTKGTGASGSF
VQTkGTGASGSF; VSKGTLVQTkGTGASGSF

Arg-C Ultra + TMT

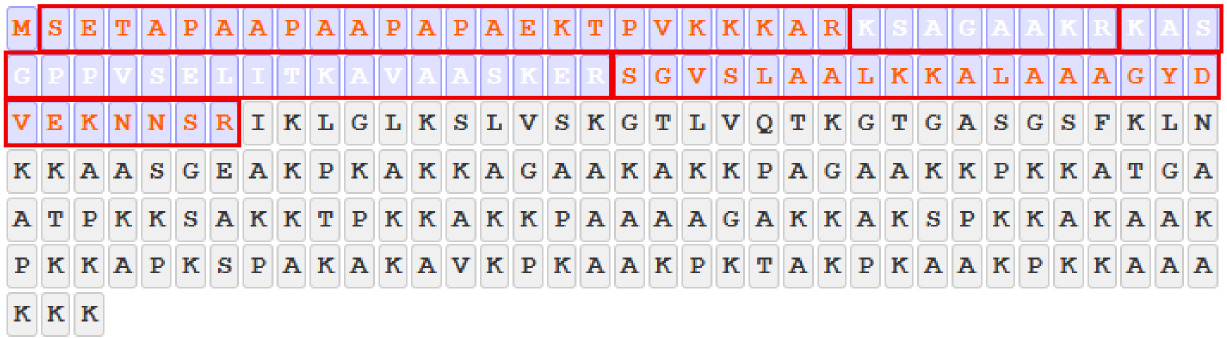

kSAGAAKR
KSAGAAkR
kASGPPVSELITKAVAASKER

KASGPPVSELITkAVAASKER
KASGPPVSELITKAVAASkER
SGVSLAALKkALAAAGYDVEKNNSR

Figure S3. H1.4 sequence coverage and succinylation sites in r-Chymotrypsin + TMT and Arg-C Ultra + TMT samples. Theoretical sequence coverage is indicated by purple shading. Red boxes indicate observed peptides/sequence coverage of succinyl-K peptides. Peptides highlighted in yellow indicate protease-specific succinyl-K site coverage.

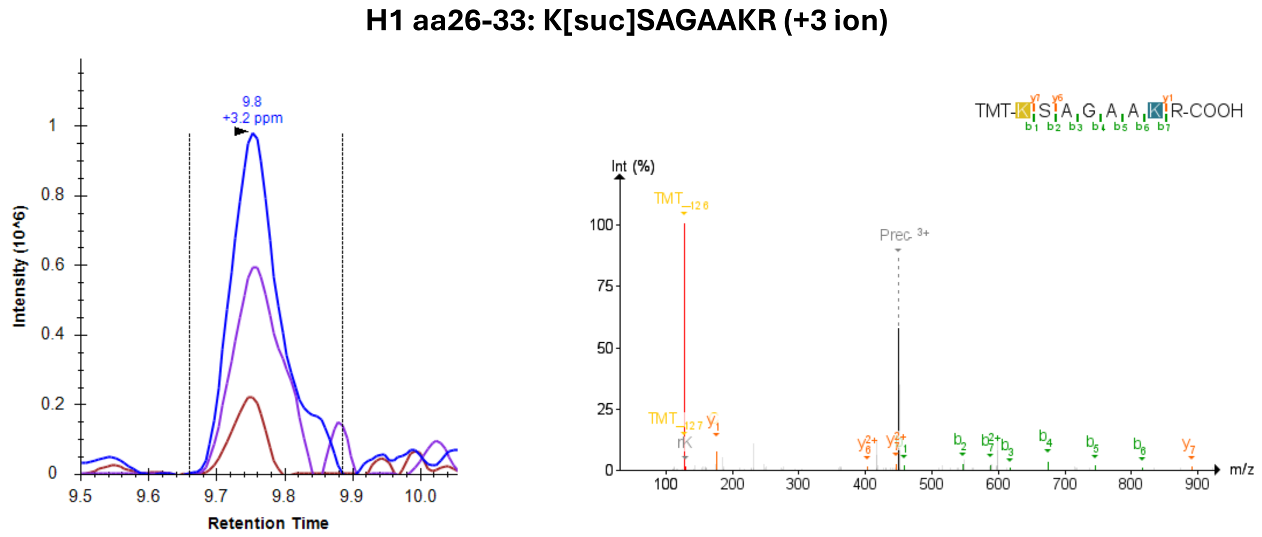

Figure S4. Representative extracted ion chromatogram (XIC) and matching mass spectrum for the H1.4 peptide KSAGAAKR (+3 ion) in Arg-C + TMT labeled samples. Peptide sequence with colors above the MS2 spectrum indicate continuous *b*-ion coverage with first position succinyl-K (yellow shading), and flanking *y*- and *b*-ions at the TMT labeling site (teal shading). Retention time of the selected peaks was matched to that reported in the FragPipe search output.

Histone H4 Succinylation sites: sequence coverage

Protease-specific site coverage

Succinylation sites identified by both Arg-C Ultra and r-Chymotrypsin

r-Chymotrypsin + TMT

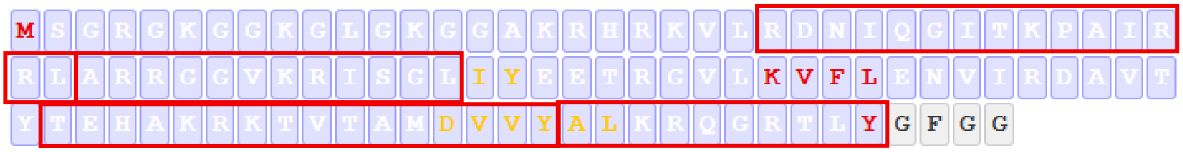

RDNIQGITkPAIRRL
ARRGGVkRISGL

TEHAKRkTVTAM;TEHAKRkTVTAMDVVY

ALkRQGRTLY; kRQGRTLY

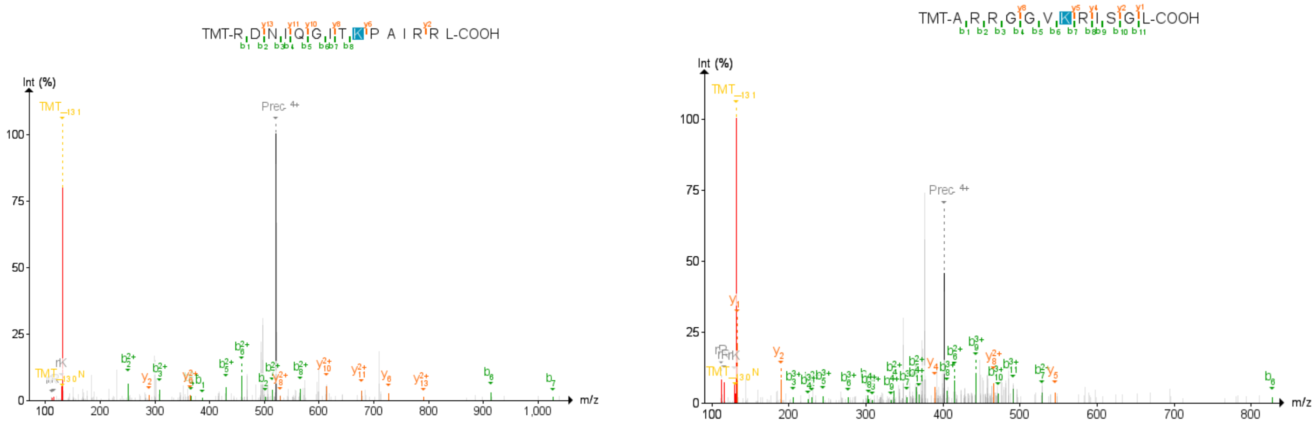

Figure S5A. H4 sequence coverage and succinylation sites in r-Chymotrypsin + TMT samples. Top panels: Theoretical sequence coverage is indicated by purple shading. Red boxes indicate observed peptides/sequence coverage of succinyl-K peptides. Peptides highlighted in yellow indicate protease-specific K-succinyl site coverage. Green highlighted K residues indicate succinyl-K sites identified by both r-Chymotrypsin + TMT and Arg-C Ultra + TMT. Bottom panels: Example fragment spectra of RDNIQGITkPAIRRL (+4 ion) and ARRGGVkRISGL (+4 ion) peptides showing increased *b*-ion series and flanking fragment ions of succinyl-K sites. Succinyl-K sites are highlighted in color.

Arg-C Ultra + TMT

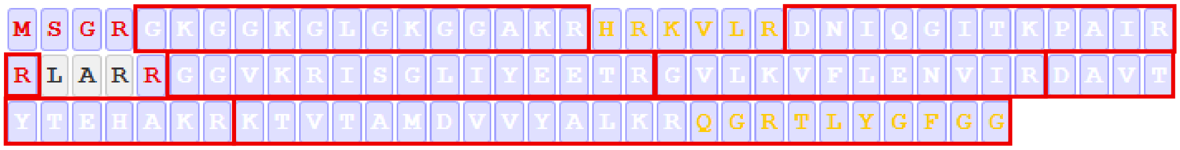

DNIQGITkPAIR; DNIQGITkPAIRR; KVLRDNIQGITkPAIR

GkGGKGLGKGGAKR

GKGGkGLGKGGAKR

DAVTYTEHAkR

kTVTAMDVVYALKR; kTVTAMDVVYALKRQGR; kTVTAMDVVYALKRQGRTLYGFGG

KTVTAMDVVYALkR

Figure S5B. H4 sequence coverage and succinylation sites in Arg-C Ultra + TMT samples. Top panels: Theoretical sequence coverage is indicated by purple shading. Red boxes indicate observed peptides/sequence coverage of succinyl-K peptides. Peptides highlighted in yellow indicate protease-specific K-succinyl site coverage. Green highlighted K residues indicate succinyl-K sites identified by both r-Chymotrypsin + TMT and Arg-C Ultra + TMT.

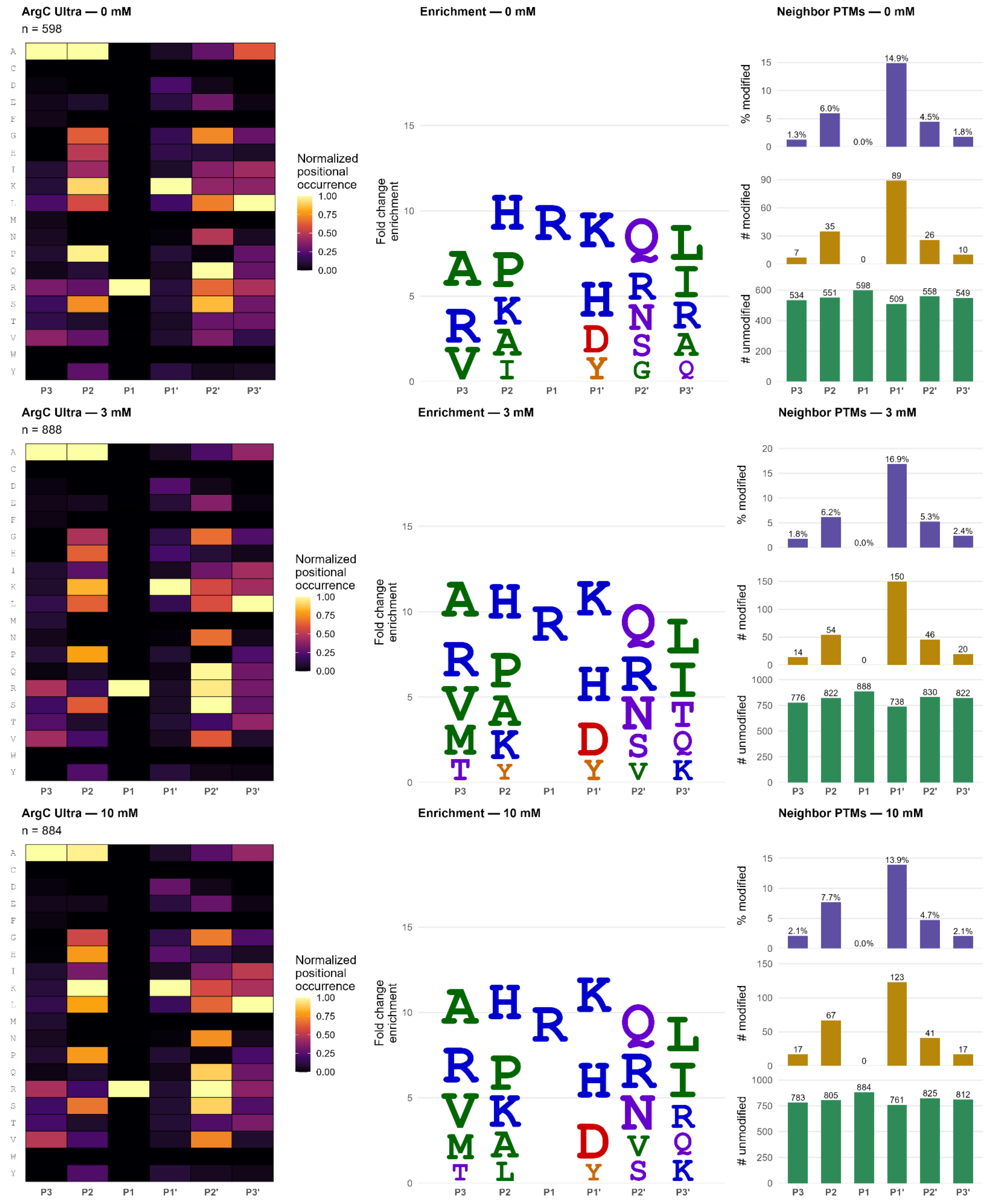

Figure S6. Arg-C Ultra missed cleavage motif analysis from NAM-treated and untreated HEK293T histones.

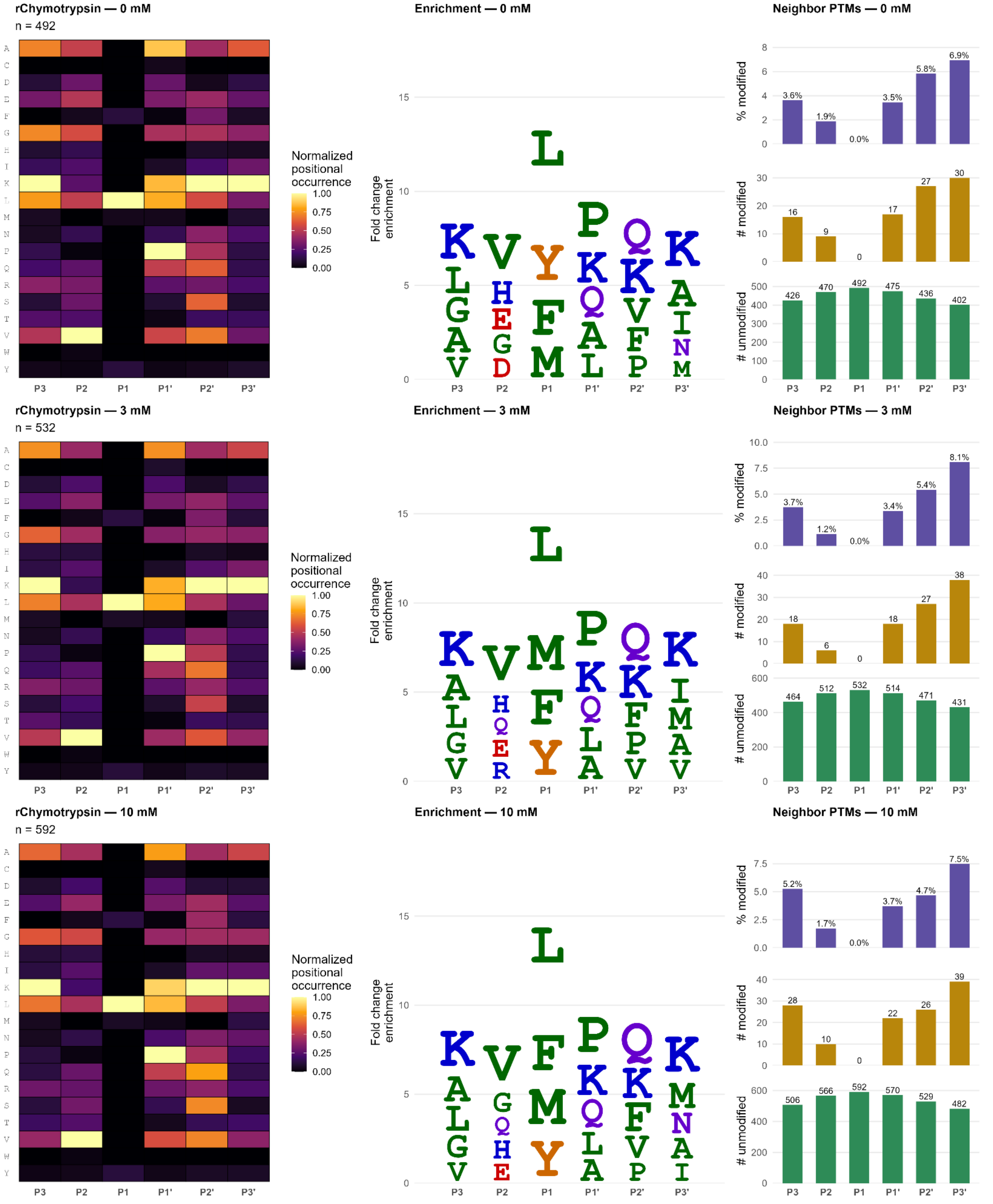

Figure S7. r-Chymotrypsin missed cleavage motif analysis from NAM-treated and untreated HEK293T histones.

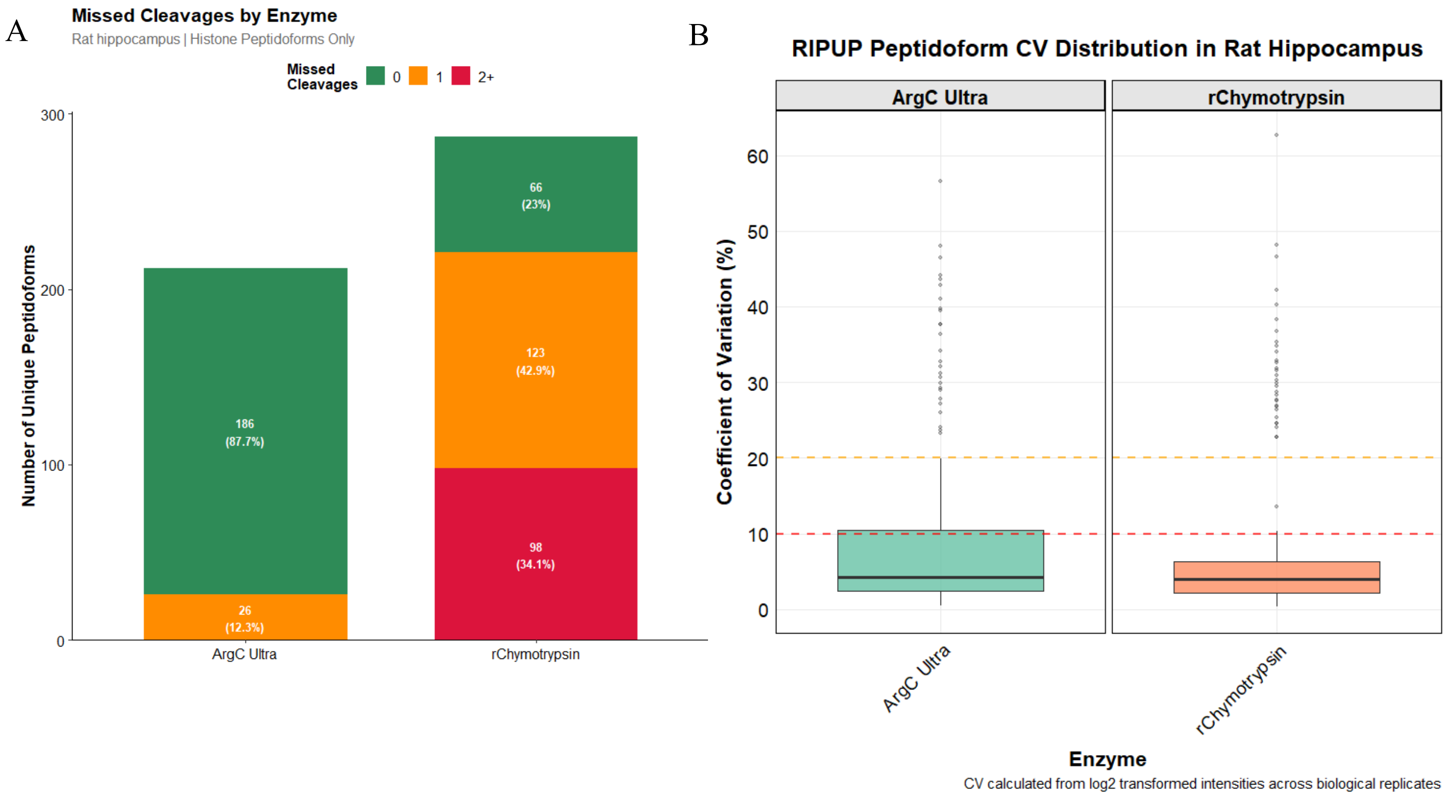

Figure S8. Peptidoforms, missed cleavages, and CV distributions of rat hippocampal histone peptides obtained with RIPUP. (A) Unique peptidoforms and missed cleavages in histone extracts from rat hippocampus digested with Arg-C Ultra (1:10; left) and r-Chymotrypsin (1:10; right). (B) CV distributions of peptidoforms identified with the RIPUP protocol in rat hippocampal sections.

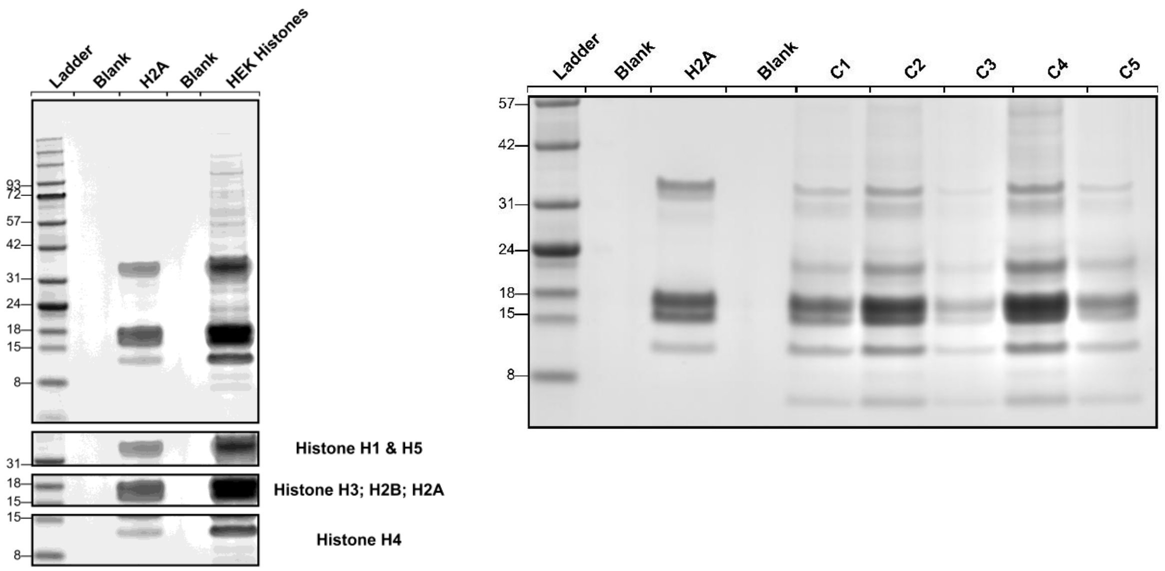

Figure S9. SDS-PAGE protein profiles of histones extracted from HEK293T cells and rat hippocampal sections. Five µL of extracted histones or 2 µg of histone standard were resolved on a 4-12% Bis-Tris mini-protein gel and subsequently stained to visualize histone protein bands and confirm sample integrity. Blank = no sample; C1-5 = histones

extracted from rat hippocampal sections, biological replicates 1 – 5; H2A = Histone type IIA from calf thymus (catalog number H9250, Sigma-Aldrich); HEK Histones = histones extracted from HEK293T nuclei.

Table S1: FragPipe Detailed Mass Offsets*

**Arg-C Ultra (unlabeled)**		**r-Chymotrypsin (unlabeled)**	
0		# Mass	Allowed sites
0.984	R	0	
27.9949	KST	27.9949	KST
68.0262	K	68.0262	K
72.0211	K	72.0211	K
104.0262	K	104.0262	K
86.0004	K	86.0004	K
86.0368	K	86.0368	K
100.016	K	100.016	K
114.0317	K	114.0317	K
56.0262	K	56.0262	K
70.0419	K	70.0419	K
114.0429	K	270.1441	K
159.0684	Q	159.0684	Q
79.9663	STY	79.9663	STY
162.0528	K	162.0528	K
108.0211	K	108.0211	K
88.016	K	88.016	K
39.9949	R	39.9949	R
54.0106	R	54.0106	R
80.0262	R	80.0262	R
144.0423	R	144.0423	R
132.0575	K	132.0575	K

* Detailed mass offsets were adjusted according to labeling strategy as described above in 'FragPipe Settings'. Highlighted row refers to the GG (Arg-C Ultra) or RGG (r-Chymotrypsin) ubiquitin remnant from the different protease cleavage specificity, resulting in distinct delta mass shifts on K.

Table S2: Protease digestion conditions for histones extracted from HEK293T cells.

<table>
<tr><th>Sample type</th><th>Protease</th><th>Denaturation</th><th>Derivatization (propionylation)</th><th>Digestion conditions: time (temperature)</th></tr>
<tr><td rowspan="3">HEK293</td><td>Trypsin (control)
1:10</td><td>+/-</td><td>+
(before and after digestion)</td><td>6 h
(37 °C)</td></tr>
<tr><td>Arg-C ultra
1:100</td><td>+/-</td><td>+/-
(after digestion)</td><td>2 h
(37 °C)</td></tr>
<tr><td>r-Chymotrypsin
1:40</td><td>+/-</td><td>+/-
(after digestion)</td><td>2 h
(RT)</td></tr>
</table>

Table S3: Denaturing conditions for histones extracted from HEK293T cells.*

<table>
<tr><th>Enzyme</th><th>Cleavage Specificity</th><th>Denaturant</th><th>Denaturant concentration</th></tr>
<tr><td>Arg-C Ultra</td><td>R; C-term (Not before P)</td><td>Urea</td><td>6 M (with 10 mM TCEP)</td></tr>
<tr><td>r-Chymotrypsin</td><td>Y, F, L, M; C-term (Not before P)</td><td>Urea</td><td>6 M (with 5 mM TCEP)</td></tr>
<tr><td>Trypsin</td><td>R, K (non-propionylated); C-term</td><td>Urea</td><td>2 M (with 5 mM TCEP)</td></tr>
<tr><td colspan="2">Arg-C Ultra</td><td rowspan="3">None</td><td>(with 10 mM TCEP)</td></tr>
<tr><td colspan="2">r-Chymotrypsin</td><td>(with 5 mM TCEP)</td></tr>
<tr><td colspan="2">Trypsin</td><td>(with 5 mM TCEP)</td></tr>
</table>

* Trypsin is reported to retain >80% activity at 2 M urea but loses activity sharply at concentrations above 4 M (Klammer & MacCoss, 2006); accordingly, 2 M urea was used for trypsin digestions under denaturing conditions. The manufacturer's protocol for Arg-C Ultra permits digestion in buffers containing up to 6 M urea, consistent with published reports of Arg-C retaining substantial activity at 4-6 M urea (Promega Technical Manual # VA1831 and VA1832). For r-Chymotrypsin, 6 M urea was found to retain cleavage specificity and efficiency at this concentration (Adoni et al., 2026). Precise protease activity measurements as a function of urea concentration were not performed in this study. Similarly, TCEP concentrations were chosen according to the manufacturer recommendations for protease tolerances; Arg-C Ultra requires a minimum of 10 mM TCEP to keep the enzyme activated, whereas r-Chymotrypsin is a serine protease with no active-site cysteine and is therefore insensitive to the redox state of cysteines. Trypsin is similarly a serine protease for which 5 mM TCEP is sufficient for substrate disulfide reduction without adverse enzyme effects.

Table S4: Peptide lists of modified peptides identified in rat hippocampal sections using the RIPUP workflow (Arg-C Ultra and r-Chymotrypsin)

Dataset_Source	Protein	Modified Sequence	Start	End	n_replicates
ArgC Ultra	H10_RAT	AKAAKKSTDHPKYSDMIVAAIQAEKNR	15	41	4
ArgC Ultra	H10_RAT	LVTTGVLKQTKGVGASGSFR	74	93	4
ArgC Ultra	H10_RAT	QSIQKYIKSHYKVGENADSQIKLSIKR	47	73	4
ArgC Ultra	H10_RAT	TENSTSTPAAKPKR	1	14	4
ArgC Ultra	H10_RAT	n[42.0106]TENSTSTPAAKPKR	1	14	4
ArgC Ultra	H11_RAT	KKPAGPSVSELIVQAVSSSKER	34	55	4
ArgC Ultra	H11_RAT	SGVSLAALKKSLAAAGYDVEKNNSR	56	80	4
ArgC Ultra	H11_RAT	SGVSLAALK[28.0313]K[28.0313]SLAAAGYDVEK[132.0575]NNSR[10.0083]	56	80	3
ArgC Ultra	H11_RAT	n[42.0106]SETAPVPQPASVAPEKPAATKKTR	1	24	4
ArgC Ultra	H14_RAT	AVKPKAAKPKTSKPKAAKPKKTAAKKK	192	218	4
ArgC Ultra	H14_RAT	KASGPPVSELITKAVAASKER	33	53	4
ArgC Ultra	H14_RAT	KASGPPVSELITK[42.0470]AVAASK[42.0470]ER[144.0423]	33	53	3
ArgC Ultra	H14_RAT	KAS[79.9663]GPPVSELITKAVAASKER	33	53	4
ArgC Ultra	H14_RAT	K[42.0106]AS[27.9949]GPPVSELITKAVAASKER	33	53	4
ArgC Ultra	H14_RAT	SETAPAAPAAPAPAEKTPIKKKAR	1	24	4
ArgC Ultra	H14_RAT	SGVSLAALKKALAAAGYDVEKNNSR	54	78	4
ArgC Ultra	H14_RAT	n[42.0106]SETAPAAPAAPAPAEKTPIKKKAR	1	24	4
ArgC Ultra	H14_RAT	n[42.0106]SETAPAAPAAPAPAEKTPIK[14.0156]KKAR	1	24	4
ArgC Ultra	H14_RAT	n[42.0106]SETAPAAPAAPAPAEKT[79.9663]PIKKKAR	1	24	4
ArgC Ultra	H14_RAT	n[42.0106]SETAPAAPAAPAPAEK[14.0156]TPIKKKAR	1	24	3
ArgC Ultra	H14_RAT	n[42.0106]SETAPAAPAAPAPAEK[42.0106]TPIKKKAR	1	24	4
ArgC Ultra	H14_RAT	n[42.0106]SET[79.9663]APAAPAAPAPAEKTPIKKKAR	1	24	4
ArgC Ultra	H15_RAT	GGVSLPALKKALAAGGYDVEKNNSR	53	77	4
ArgC Ultra	H15_RAT	KATGPPVSELITKAVSASKER	32	52	3
ArgC Ultra	H15_RAT	PKAVKSKASKPKVTKPKAAKPKAAKVKKAVSKKK	188	221	4
ArgC Ultra	H15_RAT	SETAPAETTAPAPVEKSPAKKKTKKAGAAKR	1	31	4
ArgC Ultra	H2A1C_RAT	AGLQFPVGR	21	29	4
ArgC Ultra	H2A1C_RAT	HLQLAIR	82	88	4
ArgC Ultra	H2A1C_RAT	NDEELNKLLGR	89	99	4

ArgC Ultra	H2A1C_RAT	NDEELNK[27.9949]LLGR	89	99	3
ArgC Ultra	H2A1C_RAT	VGAGAPVYLAAVLEYLTAEILELAGNAAR	43	71	3
ArgC Ultra	H2A1C_RAT	VTIAQGGVLPNIQAVLLPKKTESHHKAKGK	100	129	4
ArgC Ultra	H2A1C_RAT	VTIAQGGVLPNIQAVLLPKKTESHHKAKGK[42.0106]	100	129	4
ArgC Ultra	H2A1C_RAT	VTIAQGGVLPNIQAVLLPKKTESHHKAK[42.0106]GK	100	129	4
ArgC Ultra	H2A1C_RAT	VTIAQGGVLPNIQAVLLPKKTESHHK[42.0106]AKGK	100	129	3
ArgC Ultra	H2A1C_RAT	VTIAQGGVLPNIQAVLLPKK[114.0429]TESHHKAKGK	100	129	4
ArgC Ultra	H2A1C_RAT	VTIAQGGVLPNIQAVLLPK[114.0429]KTESHHKAKGK	100	129	3
ArgC Ultra	H2A1C_RAT	VTIAQGGVLPNIQAVLLPK[14.0156]K[72.0211]TESHHK[28.0313]AKGK	100	129	4
ArgC Ultra	H2A1C_RAT	VTIAQGGVLPNIQAVLLPK[42.0106]KTESHHKAKGK	100	129	3
ArgC Ultra	H2A1C_RAT	VTIAQGGVLPNIQAVLLPK[42.0470]K[72.0211]TESHHKAKGK	100	129	4
ArgC Ultra	H2A1C_RAT	VTIAQGGVLPNIQ[159.0684]AVLLPK[28.0313]K[28.0313]TESHHKAKGK	100	129	4
ArgC Ultra	H2A1C_RAT	VTIAQGGVLPNIQ[159.0684]AVLLPK[42.0470]K[14.0156]TESHHKAKGK	100	129	3
ArgC Ultra	H2A1F_RAT	VTIAQGGVLPNIQAVLLPKKTESHHKPKGK	100	129	4
ArgC Ultra	H2A1F_RAT	VTIAQGGVLPNIQAVLLPKKTESHHKPK[42.0106]GK	100	129	4
ArgC Ultra	H2A1F_RAT	VTIAQGGVLPNIQAVLLPKKTESHHK[114.0429]PKGK	100	129	4
ArgC Ultra	H2A1F_RAT	VTIAQGGVLPNIQAVLLPK[42.0470]K[104.0262]TESHHK[42.0106]PKGK	100	129	3
ArgC Ultra	H2A1F_RAT	VTIAQGGVLPNIQAVLLPK[88.0160]KTESHHKPKGK	100	129	2
ArgC Ultra	H2A2A_RAT	NDEELNKLLGKVTIAQGGVLPNIQAVLLPKKTESHHKAKGK	89	129	4
ArgC Ultra	H2A2A_RAT	NDEELNKLLGKVTIAQGGVLPNIQAVLLPKKTESHHKAKGK[42.0106]	89	129	4
ArgC Ultra	H2A2A_RAT	NDEELNKLLGKVTIAQGGVLPNIQAVLLPKKTESHHKAK[42.0106]GK	89	129	4

ArgC Ultra	H2A2A_RAT	NDEELNKLLGKVTIAQGGVLPNIQAVLLPK[42.0470]K[72.0211]TESHHKAKGK	89	129	4
ArgC Ultra	H2A2A_RAT	NDEELNKLLGKVTIAQGGVLPNIQAVLLPK[72.0211]KTESHHK[42.0106]AKGK	89	129	2
ArgC Ultra	H2A2A_RAT	NDEELNKLLGKVTIAQGGVLPNIQAVLLPK[72.0211]K[14.0156]TESHHK[14.0156]AK[14.0156]GK	89	129	3
ArgC Ultra	H2A2A_RAT	VGAGAPVYMAAVLEYLTAEILELAGNAAR	43	71	3
ArgC Ultra	H2A4_RAT	VTIAQGGVLPNIQAVLLPKKTESHHKSQTK	100	129	4
ArgC Ultra	H2A4_RAT	VTIAQGGVLPNIQAVLLPK[28.0313]K[68.0262]TESHHKSQTK	100	129	3
ArgC Ultra	H2AJ_RAT	VTIAQGGVLPNIQAVLLPKKTESQKVKSK	100	128	4
ArgC Ultra	H2AJ_RAT	VTIAQGGVLPNIQAVLLPKKTESQK[42.0470]VK[88.0160]SK	100	128	3
ArgC Ultra	H2AJ_RAT	VTIAQGGVLPNIQAVLLPK[114.0429]KTESQKVKSK	100	128	3
ArgC Ultra	H2AJ_RAT	VTIAQGGVLPNIQAVLLPK[14.0156]K[42.0106]TESQK[68.0262]VK[42.0106]SK[28.0313]	100	128	3
ArgC Ultra	H2AJ_RAT	VTIAQGGVLPNIQAVLLPK[42.0106]KTESQKVKSK	100	128	4
ArgC Ultra	H2AJ_RAT	VTIAQGGVLPNIQAVLLPK[42.0106]K[42.0106]TESQK[14.0156]VK[104.0262]SK	100	128	4
ArgC Ultra	H2AJ_RAT	VTIAQGGVLPNIQAVLLPK[42.0470]K[72.0211]TESQKVKSK	100	128	4
ArgC Ultra	H2AJ_RAT	VTIAQGGVLPNIQAVLLPK[88.0160]KTESQKVKSK	100	128	4
ArgC Ultra	H2AY_RAT	KLKSIAFPSIGSGR	300	313	4
ArgC Ultra	H2AY_RAT	SAKAGVIFPVGR	15	26	4
ArgC Ultra	H2AZ_RAT	AGGKAGK[42.0106]DSGKAKTKAVSR	1	19	3
ArgC Ultra	H2AZ_RAT	AGGKAGK[42.0106]DSGK[42.0106]AKTKAVSR	1	19	4
ArgC Ultra	H2AZ_RAT	AGGK[42.0106]AGK[42.0106]DSGKAKTKAVSR	1	19	4
ArgC Ultra	H2AZ_RAT	AGGK[42.0106]AGK[42.0106]DSGK[42.0106]AKTKAVSR	1	19	4
ArgC Ultra	H2AZ_RAT	GDEELDSLIKATIAGGGVIPHIHKSLIGKKGQQKTV	92	127	4
ArgC Ultra	H2AZ_RAT	GDEELDSLIKATIAGGGVIPHIHKSLIGK[42.0470]K[72.0211]GQQKTV	92	127	4
ArgC Ultra	H2AZ_RAT	GDEELDSLIKATIAGGGVIPHIHK[42.0470]SLIGK[72.0211]KGQQKTV	92	127	3
ArgC Ultra	H2AZ_RAT	GDEELDSLIKATIAGGGVIPHIHK[72.0211]SLIGK[42.0106]KGQQKTV	92	127	2
ArgC Ultra	H2B1_RAT	EIQTAVR	92	98	4
ArgC Ultra	H2B1_RAT	KESYSVYVYKVLKQVHPDTGISSKAMGIMNSFVNDIFER	34	72	3
ArgC Ultra	H2B1_RAT	K[14.0156]ESYSVYVYKVLKQVHPDTGISSKAMGIMNSFVNDIFER	34	72	3
ArgC Ultra	H2B1_RAT	LLLPGELAKHAVSEGTKAVTKYTSSK	99	124	4
ArgC Ultra	H2B1_RAT	LLLPGELAKHAVSEGTKAVTKYTSSK[114.0429]	99	124	4
ArgC Ultra	H2B1_RAT	LLLPGELAKHAVSEGTKAVTK[114.0429]YTSSK	99	124	4

ArgC Ultra	H2B1_RAT	LLLPGELAKHAVSEGTKAVTK[42.0106]YTSSK[72.0211]	99	124	3
ArgC Ultra	H2B1_RAT	LLLPGELAK[27.9949]HAVSEGTKAVTKYTSSK	99	124	4
ArgC Ultra	H2B1_RAT	PEPAKSR[39.9949]PAPKKGSKKAVTKAQKKDGKER	1	29	3
ArgC Ultra	H31_RAT	K[28.0313]SAPATGGVKKPHR	27	40	4
ArgC Ultra	H31_RAT	K[28.0313]SAPATGGVK[14.0156]KPHR	27	40	4
ArgC Ultra	H31_RAT	K[28.0313]SAPATGGVK[28.0313]KPHR	27	40	3
ArgC Ultra	H31_RAT	K[42.0470]SAPATGGVKKPHR	27	40	4
ArgC Ultra	H31_RAT	K[42.0470]SAPATGGVK[28.0313]KPHR	27	40	4
ArgC Ultra	H33_RAT	EIAQDFKTDLR	73	83	4
ArgC Ultra	H33_RAT	EIAQDFK[14.0156]TDLR	73	83	4
ArgC Ultra	H33_RAT	EIAQDFK[28.0313]TDLR	73	83	4
ArgC Ultra	H33_RAT	KLPFQR	64	69	4
ArgC Ultra	H33_RAT	KQLATK[42.0106]AAR	18	26	4
ArgC Ultra	H33_RAT	KSAPSTGGVKKPHR	27	40	4
ArgC Ultra	H33_RAT	KSAPSTGGVK[28.0313]KPHR	27	40	4
ArgC Ultra	H33_RAT	KSAPSTGGVK[42.0470]KPHR	27	40	4
ArgC Ultra	H33_RAT	K[14.0156]S[27.9949]APSTGGVK[14.0156]KPHR	27	40	4
ArgC Ultra	H33_RAT	K[14.0156]S[27.9949]APSTGGVK[28.0313]KPHR	27	40	4
ArgC Ultra	H33_RAT	K[14.0156]S[27.9949]APSTGGVK[42.0470]KPHR	27	40	3
ArgC Ultra	H33_RAT	K[28.0313]SAPSTGGVK[14.0156]KPHR	27	40	4
ArgC Ultra	H33_RAT	K[28.0313]SAPSTGGVK[28.0313]KPHR	27	40	4
ArgC Ultra	H33_RAT	K[42.0106]QLATKAAR	18	26	4
ArgC Ultra	H33_RAT	K[42.0106]QLATK[42.0106]AAR	18	26	4
ArgC Ultra	H33_RAT	K[42.0106]SAPSTGGVK[28.0313]KPHR	27	40	3
ArgC Ultra	H33_RAT	K[42.0106]STGGK[42.0106]APR	9	17	4
ArgC Ultra	H33_RAT	K[42.0470]SAPSTGGVK[14.0156]KPHR	27	40	4
ArgC Ultra	H33_RAT	K[42.0470]SAPSTGGVK[28.0313]KPHR	27	40	4
ArgC Ultra	H33_RAT	VTIMPKDIQLAR	117	128	4
ArgC Ultra	H33_RAT	YQKSTELLIR	54	63	4
ArgC Ultra	H4_RAT	DAVTYTEHAKR	68	78	4
ArgC Ultra	H4_RAT	DNIQGITKPAIR	24	35	4
ArgC Ultra	H4_RAT	GKGGKGLGK[42.0106]GGAK[42.0106]R	4	17	4

ArgC Ultra	H4_RAT	GKGGK[42.0106]GLGKGGAK[42.0106]R	4	17	4
ArgC Ultra	H4_RAT	GKGGK[42.0106]GLGK[42.0106]GGAKR	4	17	4
ArgC Ultra	H4_RAT	GKGGK[42.0106]GLGK[42.0106]GGAK[42.0106]R	4	17	4
ArgC Ultra	H4_RAT	GK[42.0106]GGKGLGKGGAK[42.0106]R	4	17	4
ArgC Ultra	H4_RAT	GK[42.0106]GGKGLGK[42.0106]GGAK[42.0106]R	4	17	4
ArgC Ultra	H4_RAT	GK[42.0106]GGK[42.0106]GLGKGGAK[42.0106]R	4	17	4
ArgC Ultra	H4_RAT	GK[42.0106]GGK[42.0106]GLGK[42.0106]GGAK[27.9949]R[14.0156]	4	17	4
ArgC Ultra	H4_RAT	GK[42.0106]GGK[42.0106]GLGK[42.0106]GGAK[42.0106]R	4	17	4
ArgC Ultra	H4_RAT	GVLKVFLENVIR	56	67	4
ArgC Ultra	H4_RAT	GVLK[162.0528]VFLENVIR	56	67	4
ArgC Ultra	H4_RAT	ISGLIYEETR	46	55	4
ArgC Ultra	H4_RAT	IS[27.9949]GLIYEETR	46	55	4
ArgC Ultra	H4_RAT	IS[79.9663]GLIYEETR	46	55	4
ArgC Ultra	H4_RAT	KTVTAMDVVYALKR	79	92	4
ArgC Ultra	H4_RAT	KTVTAMDVVYALK[162.0528]R	79	92	4
ArgC Ultra	H4_RAT	KT[27.9949]VTAMDVVYALKR	79	92	4
ArgC Ultra	H4_RAT	K[162.0528]TVTAMDVVYALKR	79	92	4
ArgC Ultra	H4_RAT	K[42.0106]T[27.9949]VTAMDVVYALKR	79	92	4
ArgC Ultra	H4_RAT	SGR[14.0156]GK[42.0106]GGKGLGKGGAK[42.0106]R	1	17	4
ArgC Ultra	H4_RAT	n[42.0106]SGR[14.0156]GKGGKGLGKGGAK[42.0106]R	1	17	4
ArgC Ultra	H4_RAT	n[42.0106]SGR[14.0156]GKGGK[42.0106]GLGKGGAK[42.0106]R	1	17	4
ArgC Ultra	H4_RAT	n[42.0106]SGR[14.0156]GK[14.0156]GGKGLGKGGAK[42.0106]R	1	17	4
ArgC Ultra	H4_RAT	n[42.0106]SGR[28.0313]GKGGKGLGKGGAK[42.0106]R	1	17	4
rChymotrypsin	H10_RAT	AKGDEPKRSVAF	95	106	4
rChymotrypsin	H10_RAT	KQTKGVGASGSF	81	92	4
rChymotrypsin	H10_RAT	KQTKGVGASGS[-18.0106]F	81	92	4
rChymotrypsin	H10_RAT	KVGENADSQIKL	58	69	4
rChymotrypsin	H10_RAT	KVGENADSQIKLSIKRLVTTGVL	58	80	4
rChymotrypsin	H10_RAT	KVGENADS[-18.0106]QIKL	58	69	4
rChymotrypsin	H10_RAT	RLAKGDEPKRSVAF	93	106	4
rChymotrypsin	H10_RAT	SDMIVAAIQAEKNRAGSSRQSIQKY	28	52	4
rChymotrypsin	H10_RAT	SDM[15.9949]IVAAIQAEKNRAGSSRQSIQKY	28	52	4

rChymotrypsin	H10_RAT	SDM[15.9949]IVAAIQAEK[14.0156]NR[39.9949]AGS[-18.0106]S[-18.0106]RQS[-18.0106]IQKY	28	52	2
rChymotrypsin	H10_RAT	SDM[15.9949]IVAAIQAEK[42.0106]NR[10.0083]AGS[-18.0106]S[-18.0106]RQSIQKY	28	52	3
rChymotrypsin	H10_RAT	SIKRLVTTGVL	70	80	4
rChymotrypsin	H10_RAT	SIKRLVTTGVLKQTKGVGASGSF	70	92	4
rChymotrypsin	H10_RAT	TENSTSTPAAKPKRAKAAKKSTDHPKY	1	27	4
rChymotrypsin	H10_RAT	VTTGVLKQTKGVGASGSF	75	92	4
rChymotrypsin	H11_RAT	KK[42.0106]SLAAAGY	64	72	3
rChymotrypsin	H11_RAT	KSLVNKGTLVQTKGTGAAGSF	86	106	4
rChymotrypsin	H14_RAT	AAAGYDVEKNNSRIKL	66	81	4
rChymotrypsin	H14_RAT	AAAGYDVEKNNSRIKLGL	66	83	4
rChymotrypsin	H14_RAT	AAAGYDVEK[114.0317]NNSR[28.0313]IK[28.0313]L	66	81	3
rChymotrypsin	H14_RAT	AALKKALAAAGY	59	70	4
rChymotrypsin	H14_RAT	DVEKNNSRIKL	71	81	4
rChymotrypsin	H14_RAT	DVEKNNSRIKLGL	71	83	4
rChymotrypsin	H14_RAT	DVEKNNSRIKLGLKSL	71	86	4
rChymotrypsin	H14_RAT	GLKSLVSKGTL	82	92	4
rChymotrypsin	H14_RAT	ITKAVAASKERSGVSL	43	58	4
rChymotrypsin	H14_RAT	ITKAVAASKERSGVSLAAL	43	61	4
rChymotrypsin	H14_RAT	ITKAVAASKERSGVSLAALKKAL	43	65	4
rChymotrypsin	H14_RAT	KKALAAAGY	62	70	4
rChymotrypsin	H14_RAT	KKALAAAGYDVEKNNSRIKL	62	81	4
rChymotrypsin	H14_RAT	KKALAAAGYDVEK[114.0317]NNSR[14.0156]IK[42.0470]L	62	81	2
rChymotrypsin	H14_RAT	KKALAAAGYDVEK[132.0575]NNSR[10.0083]IK[28.0313]L	62	81	4
rChymotrypsin	H14_RAT	KKALAAAGYDVEK[70.0419]NNSR[28.0313]IK[72.0211]L	62	81	4
rChymotrypsin	H14_RAT	KSLVSKGTL	84	92	4
rChymotrypsin	H14_RAT	KSLVSKGTLVQTKGTGASGSF	84	104	4
rChymotrypsin	H14_RAT	KSLVSKGTLVQTKGTGASGS[-18.0106]F	84	104	3
rChymotrypsin	H14_RAT	K[14.0156]SLVSK[114.0317]GTLVQTK[42.0470]GTGASGSF	84	104	2
rChymotrypsin	H14_RAT	K[14.0156]SLVSK[70.0419]GTLVQTK[86.0368]GTGASGSF	84	104	3
rChymotrypsin	H14_RAT	K[42.0106]SLVSK[42.0470]GTLVQTK[86.0368]GTGASGSF	84	104	4
rChymotrypsin	H14_RAT	K[70.0419]S[27.9949]LVSK[42.0470]GTLVQTK[42.0470]GTGASGSF	84	104	3
rChymotrypsin	H14_RAT	VQTKGTGASGSF	93	104	4

rChymotrypsin	H14_RAT	VSKGTLVQTKGTGASGSF	87	104	4
rChymotrypsin	H15_RAT	AAGGYDVEKNNSRIKL	65	80	4
rChymotrypsin	H15_RAT	AAGGYDVEKNNSRIKLGL	65	82	4
rChymotrypsin	H15_RAT	ITKAVSASKERGGVSLPAL	42	60	4
rChymotrypsin	H15_RAT	ITKAVSASKERGGVSLPALKKAL	42	64	3
rChymotrypsin	H15_RAT	KKALAAGGYDVEKNNSRIKL	61	80	4
rChymotrypsin	H15_RAT	KKALAAGGYDVEK[114.0317]NNSR[14.0156]IK[42.0470]L	61	80	2
rChymotrypsin	H15_RAT	KKALAAGGYDVEK[70.0419]NNSR[28.0313]IK[72.0211]L	61	80	4
rChymotrypsin	H2A1F_RAT	LRKGNYSERVGAGAPVY	34	50	4
rChymotrypsin	H2A1F_RAT	RKGNYSERVGAGAPVY	35	50	4
rChymotrypsin	H2A1F_RAT	RKGNYSERVGAGAPVYL	35	51	4
rChymotrypsin	H2A1F_RAT	SERVGAGAPVY	40	50	4
rChymotrypsin	H2A1F_RAT	SERVGAGAPVYL	40	51	4
rChymotrypsin	H2A1F_RAT	SGRGKQGGK[27.9949]ARAKAKTRSSRAGLQFPVGRVHRL	1	33	4
rChymotrypsin	H2A1F_RAT	TAEILELAANAAR[144.0423]DNK[42.0470]K[14.0156]TR[14.0156]IIPR[14.0156]HL	59	83	4
rChymotrypsin	H2A2A_RAT	AERVGAGAPVY	40	50	4
rChymotrypsin	H2A2A_RAT	AERVGAGAPVYM	40	51	4
rChymotrypsin	H2A2A_RAT	AERVGAGAPVYM[15.9949]	40	51	4
rChymotrypsin	H2A2A_RAT	AGNAARDNKKTRIIPRHL	66	83	4
rChymotrypsin	H2A2A_RAT	AGNAARDNKKTRIIPRHLQL	66	85	4
rChymotrypsin	H2A2A_RAT	AIRNDEEL	86	93	4
rChymotrypsin	H2A2A_RAT	AIRNDEELNKL	86	96	4
rChymotrypsin	H2A2A_RAT	AIRNDEELNKLL	86	97	4
rChymotrypsin	H2A2A_RAT	ELAGNAARDNKKTRIIPRHL	64	83	4

rChymotrypsin	H2A2A_RAT	ELAGNAARDNKKTRIIPRHLQL	64	85	4
rChymotrypsin	H2A2A_RAT	E[-18.0106]LAGNAARDNKKTRIIPRHL	64	83	4
rChymotrypsin	H2A2A_RAT	E[-18.0106]LAGNAARDNKKTRIIPRHLQL	64	85	4
rChymotrypsin	H2A2A_RAT	LRKGNYAERVGAGAPVY	34	50	4
rChymotrypsin	H2A2A_RAT	PNIQAVLLPKKTESHHKAKGK	109	129	4
rChymotrypsin	H2A2A_RAT	PVGRVHRL	26	33	4
rChymotrypsin	H2A2A_RAT	PVGRVHRLL	26	34	4
rChymotrypsin	H2A2A_RAT	PVGRVHRLLRKGNY	26	39	4
rChymotrypsin	H2A2A_RAT	QFPVGRVHRL	24	33	4
rChymotrypsin	H2A2A_RAT	QFPVGRVHRLL	24	34	4
rChymotrypsin	H2A2A_RAT	QLAIRNDEEL	84	93	3
rChymotrypsin	H2A2A_RAT	QLAIRNDEELNKL	84	96	4
rChymotrypsin	H2A2A_RAT	RKGNYAERVGAGAPVY	35	50	4
rChymotrypsin	H2A2A_RAT	RKGNYAERVGAGAPVYM	35	51	4
rChymotrypsin	H2A2A_RAT	RKGNYAERVGAGAPVYM[15.9949]	35	51	4
rChymotrypsin	H2A2A_RAT	TAEILELAGNAARDNKKTRIIPRHL	59	83	4
rChymotrypsin	H2A2A_RAT	TAEILELAGNAAR[10.0083]DNK[70.0419]K[56.0262]TR[10.0083]IIPR[10.0083]HL	59	83	4
rChymotrypsin	H2AJ_RAT	AERVGAGAPVYL	40	51	4
rChymotrypsin	H2AJ_RAT	RKGNYAERVGAGAPVYL	35	51	4
rChymotrypsin	H2AY_RAT	AVANDEELNQL	83	93	3
rChymotrypsin	H2AY_RAT	AVANDEELNQLL	83	94	4
rChymotrypsin	H2AY_RAT	ELAGNAARDNKKGRVTPRHILL	61	82	4
rChymotrypsin	H2AY_RAT	ELAGNAAR[10.0083]DNK[28.0313]KGR[10.0083]VTPR[10.0083]HILL	61	82	4
rChymotrypsin	H2AY_RAT	ELAGNAAR[10.0083]DNK[56.0262]KGR[10.0083]VTPR[10.0083]HILL	61	82	4

rChymotrypsin	H2AY_RAT	ILKAISSY	325	332	3
rChymotrypsin	H2AY_RAT	KGVTIASGGVLPNIHPELL	95	113	4
rChymotrypsin	H2AY_RAT	M[15.9949]AAVLEY	48	54	2
rChymotrypsin	H2AY_RAT	RIGVGAPVY	39	47	4
rChymotrypsin	H2AY_RAT	RKKNGPLEVAGAAVSAGHGLPAKF	244	267	4
rChymotrypsin	H2AY_RAT	R[144.0423]K[28.0313]K[70.0419]NGPLEVAGAAVSAGHGLPAKF	244	267	4
rChymotrypsin	H2AY_RAT	SSSIKTVY	338	345	3
rChymotrypsin	H2AY_RAT	SSSIKTVYF	338	346	4
rChymotrypsin	H2AY_RAT	STKSLFL	185	191	3
rChymotrypsin	H2AY_RAT	TAEILELAGNAARDNKKGRVTPRHIL	56	81	3
rChymotrypsin	H2AY_RAT	TAEILELAGNAARDNK[28.0313]K[14.0156]GR[14.0156]VTPR[14.0156]HIL	56	81	4
rChymotrypsin	H2AY_RAT	TAEILELAGNAARDNK[70.0419]K[86.0368]GR[14.0156]VTPR[28.0313]HIL	56	81	4
rChymotrypsin	H2AY_RAT	TAEILELAGNAAR[10.0083]DNK[28.0313]K[70.0419]GR[10.0083]VTPR[80.0262]HIL	56	81	4
rChymotrypsin	H2AY_RAT	TAEILELAGNAAR[14.0156]DNK[42.0470]K[14.0156]GRVTPRHIL	56	81	4
rChymotrypsin	H2AY_RAT	TAEILELAGNAAR[28.0313]DNK[70.0419]K[42.0470]GRVTPR[39.9949]HIL	56	81	4
rChymotrypsin	H2AY_RAT	TVLSTKSLF	182	190	4
rChymotrypsin	H2AY_RAT	VQEMAKLDAN	358	367	4
rChymotrypsin	H2AY_RAT	VQEM[15.9949]AKLDAN	358	367	4
rChymotrypsin	H2AY_RAT	VSTM[15.9949]SSSIKTVYF	334	346	4
rChymotrypsin	H2AZ_RAT	AGGKAGKDSGKAKTKAVSRSQRAGL	1	25	4
rChymotrypsin	H2AZ_RAT	AGGKAGKDSGKAKTKAVSRSQRAGLQFPVGRIHRHL	1	36	4
rChymotrypsin	H2AZ_RAT	AGNASKDLKVKRITPRHLQL	69	88	4
rChymotrypsin	H2AZ_RAT	AGNASKDLK[28.0313]VK[70.0419]R[144.0423]ITPRHLQL	69	88	4
rChymotrypsin	H2AZ_RAT	AIRGDEELDSL	89	99	4
rChymotrypsin	H2AZ_RAT	AIRGDEELDSLIKATIAGGGVIPHIHKSL	89	117	2
rChymotrypsin	H2AZ_RAT	ELAGNASKDL	67	76	4
rChymotrypsin	H2AZ_RAT	ELAGNASKDLKVKRITPRHL	67	86	4
rChymotrypsin	H2AZ_RAT	IKATIAGGGVIPHIHKSL	100	117	4
rChymotrypsin	H2AZ_RAT	IKATIAGGGVIPHIHKSLIGKKGQQKTV	100	127	4
rChymotrypsin	H2AZ_RAT	KSRTTSHGRVGATAAVY	37	53	4
rChymotrypsin	H2AZ_RAT	KVKRITPRHLQL	77	88	4
rChymotrypsin	H2AZ_RAT	QFPVGRIHRHL	26	36	4

rChymotrypsin	H2AZ_RAT	QLAIRGDEELDSL	87	99	4
rChymotrypsin	H2B1A_RAT	ERIAGEASRL	72	81	4
rChymotrypsin	H2B1A_RAT	ERIAGEASRLAHY	72	84	4
rChymotrypsin	H2B1A_RAT	E[-18.0106]RIAGEASRL	72	81	4
rChymotrypsin	H2B1A_RAT	E[-18.0106]RIAGEASRLAHY	72	84	4
rChymotrypsin	H2B1A_RAT	KQVHPDTGISSK[56.0262]AM[15.9949]S[27.9949]IM	47	63	4
rChymotrypsin	H2B1A_RAT	KQVHPDTGISS[-18.0106]K[70.0419]AM[15.9949]SIM[15.9949]	47	63	3
rChymotrypsin	H2B1A_RAT	KQVHPDTGIS[-18.0106]S[-18.0106]K[56.0262]AMS[-18.0106]IMNSF	47	66	3
rChymotrypsin	H2B1A_RAT	KVLKQVHPDTGISSK[270.1441]AMSIM[15.9949]	44	63	2
rChymotrypsin	H2B1A_RAT	KVLKQVHPDTGISSK[270.1441]AM[15.9949]SIM[15.9949]	44	63	3
rChymotrypsin	H2B1A_RAT	KVLKQVHPDTGISSK[56.0262]AM[15.9949]S[27.9949]IM[15.9949]	44	63	4
rChymotrypsin	H2B1A_RAT	KVLKQVHPDTGISS[27.9949]K[56.0262]AMSIM	44	63	2
rChymotrypsin	H2B1A_RAT	KVLKQVHPDTGISS[27.9949]K[56.0262]AM[15.9949]SIM	44	63	3
rChymotrypsin	H2B1A_RAT	KVLKQVHPDTGIS[-18.0106]S[-18.0106]K[42.0470]AM[15.9949]S[27.9949]IM[15.9949]	44	63	3
rChymotrypsin	H2B1A_RAT	PEVSAKGTTISKKGF	1	15	4
rChymotrypsin	H2B1_RAT	AHYNKRSTITSREIQTAVRL	80	99	4
rChymotrypsin	H2B1_RAT	AKHAVSEGTKAVTKY	106	120	4
rChymotrypsin	H2B1_RAT	GIMNSFVNDIF	60	70	4
rChymotrypsin	H2B1_RAT	GIM[15.9949]NSFVNDIF	60	70	4
rChymotrypsin	H2B1_RAT	KQVHPDTGISSKAM	46	59	4
rChymotrypsin	H2B1_RAT	KQVHPDTGISSKAMGIM	46	62	4
rChymotrypsin	H2B1_RAT	KQVHPDTGISSKAMGIMNSF	46	65	4
rChymotrypsin	H2B1_RAT	KQVHPDTGISSKAMGIMNS[-18.0106]F	46	65	4
rChymotrypsin	H2B1_RAT	KQVHPDTGISSKAMGIM[15.9949]	46	62	4
rChymotrypsin	H2B1_RAT	KQVHPDTGISSKAMGIM[15.9949]NSF	46	65	4

rChymotrypsin	H2B1_RAT	KQVHPDTGISSKAM[15.9949]	46	59	4
rChymotrypsin	H2B1_RAT	KQVHPDTGISSKAM[15.9949]GIM	46	62	4
rChymotrypsin	H2B1_RAT	KQVHPDTGISSKAM[15.9949]GIMNSF	46	65	4
rChymotrypsin	H2B1_RAT	KQVHPDTGISSKAM[15.9949]GIMNS[-18.0106]F	46	65	4
rChymotrypsin	H2B1_RAT	KQVHPDTGISSKAM[15.9949]GIM[15.9949]	46	62	4
rChymotrypsin	H2B1_RAT	KQVHPDTGISSKAM[15.9949]GIM[15.9949]NSF	46	65	4
rChymotrypsin	H2B1_RAT	KVLKQVHPDTGISSKAM	43	59	4
rChymotrypsin	H2B1_RAT	KVLKQVHPDTGISSKAMGIM	43	62	4
rChymotrypsin	H2B1_RAT	KVLKQVHPDTGISSKAMGIM[15.9949]	43	62	4
rChymotrypsin	H2B1_RAT	KVLKQVHPDTGISSKAM[15.9949]	43	59	4
rChymotrypsin	H2B1_RAT	KVLKQVHPDTGISSKAM[15.9949]GIM	43	62	4
rChymotrypsin	H2B1_RAT	KVLKQVHPDTGISSKAM[15.9949]GIM[15.9949]	43	62	4
rChymotrypsin	H2B1_RAT	KVLKQVHPDTGIS[-18.0106]SKAM	43	59	4
rChymotrypsin	H2B1_RAT	KVLKQVHPDTGIS[-18.0106]SKAMGIM	43	62	4
rChymotrypsin	H2B1_RAT	KVLKQVHPDTGIS[-18.0106]SKAM[15.9949]	43	59	4
rChymotrypsin	H2B1_RAT	KVLKQVHPDTGIS[-18.0106]SKAM[15.9949]GIM	43	62	3
rChymotrypsin	H2B1_RAT	KVLKQVHPDTGIS[-18.0106]S[-18.0106]KAM	43	59	4
rChymotrypsin	H2B1_RAT	K[114.0317]VLKQVHPDTGISSKAM[15.9949]GIM	43	62	4
rChymotrypsin	H2B1_RAT	K[27.9949]VLKQVHPDTGISSKAM	43	59	3
rChymotrypsin	H2B1_RAT	K[27.9949]VLKQVHPDTGISSKAM[15.9949]	43	59	2
rChymotrypsin	H2B1_RAT	LPGELAKHAVSEGTKAVTKY	101	120	4
rChymotrypsin	H2B1_RAT	LPGELAK[42.0470]HAVSEGTK[70.0419]AVTKY	101	120	4
rChymotrypsin	H2B1_RAT	NKRSTITSREIQTAVRL	83	99	4
rChymotrypsin	H2B1_RAT	NKRSTITSREIQTAVRLL	83	100	4
rChymotrypsin	H2B1_RAT	NKRS[-18.0106]TITSREIQTAVRL	83	99	4
rChymotrypsin	H2B1_RAT	NK[27.9949]RSTITSREIQTAVRL	83	99	3
rChymotrypsin	H2B1_RAT	PGELAKHAVSEGTKAVTKY	102	120	4
rChymotrypsin	H2B1_RAT	VYKVLKQVHPDTGISSKAM[15.9949]	41	59	3
rChymotrypsin	H31_RAT	ALQEACEAY	91	99	4
rChymotrypsin	H31_RAT	QSSAVM[15.9949]AL	85	92	4
rChymotrypsin	H31_RAT	QSSAVM[15.9949]ALQEACEAY	85	99	4
rChymotrypsin	H31_RAT	RFQSSAVM	83	90	4

rChymotrypsin	H33_RAT	EDTNLCAIHAKRVTIMPKDIQL	105	126	4
rChymotrypsin	H33_RAT	EDTNLCAIHAKRVTIM[15.9949]PKDIQL	105	126	4
rChymotrypsin	H33_RAT	E[-18.0106]DTNLCAIHAKRVTIMPKDIQL	105	126	4
rChymotrypsin	H33_RAT	E[-18.0106]DTNLCAIHAK[56.0262]R[10.0083]VTIMPKDIQL	105	126	3
rChymotrypsin	H33_RAT	IRKLPF	62	67	4
rChymotrypsin	H33_RAT	IRKLPFQRL	62	70	4
rChymotrypsin	H33_RAT	KTDLRF	79	84	4
rChymotrypsin	H33_RAT	K[14.0156]TDLRF	79	84	4
rChymotrypsin	H33_RAT	K[28.0313]TDLRF	79	84	4
rChymotrypsin	H33_RAT	LIRKLPF	61	67	4
rChymotrypsin	H33_RAT	QKSTELL	55	61	4
rChymotrypsin	H33_RAT	QKS[-18.0106]TELL	55	61	4
rChymotrypsin	H33_RAT	QRLVREIAQDF	68	78	4
rChymotrypsin	H33_RAT	QRLVREIAQDFKTDL	68	82	4
rChymotrypsin	H33_RAT	QRLVREIAQDFK[28.0313]TDL	68	82	4
rChymotrypsin	H33_RAT	QSAAIGALQEASEAY	85	99	4
rChymotrypsin	H33_RAT	QSAAIGALQEAS[-18.0106]EAY	85	99	4
rChymotrypsin	H33_RAT	QS[-18.0106]AAIGALQEASEAY	85	99	4
rChymotrypsin	H33_RAT	RFQSAAIGALQEASEAY	83	99	4
rChymotrypsin	H33_RAT	RPGTVALREIRRY	42	54	4
rChymotrypsin	H33_RAT	VREIAQDF	71	78	4
rChymotrypsin	H33_RAT	VREIAQDFKTDL	71	82	4
rChymotrypsin	H33_RAT	VREIAQDFKTDLRF	71	84	4
rChymotrypsin	H33_RAT	VREIAQDFK[14.0156]TDL	71	82	4
rChymotrypsin	H33_RAT	VREIAQDFK[14.0156]TDLRF	71	84	4
rChymotrypsin	H33_RAT	VREIAQDFK[28.0313]TDL	71	82	3
rChymotrypsin	H33_RAT	VREIAQDFK[28.0313]TDLRF	71	84	4
rChymotrypsin	H4_RAT	ALKRQGRTLY	89	98	4
rChymotrypsin	H4_RAT	ARRGGVKRISGL	38	49	4
rChymotrypsin	H4_RAT	ARRGGVKRISGLIY	38	51	4
rChymotrypsin	H4_RAT	EETRGVL	52	58	4
rChymotrypsin	H4_RAT	EETRGVLKVF	52	61	4

rChymotrypsin	H4_RAT	ENVIRDAVTY	63	72	4
rChymotrypsin	H4_RAT	E[-18.0106]ETRGVL	52	58	3
rChymotrypsin	H4_RAT	E[-18.0106]NVIRDAVTY	63	72	4
rChymotrypsin	H4_RAT	IYEETRGVL	50	58	4
rChymotrypsin	H4_RAT	IYEETRGVLKVF	50	61	4
rChymotrypsin	H4_RAT	KRQGRTLY	91	98	4
rChymotrypsin	H4_RAT	KVFLENVIRDAVTY	59	72	4
rChymotrypsin	H4_RAT	LENVIRDAVTY	62	72	4
rChymotrypsin	H4_RAT	RDNIQGITKPAIRRLARRGGVKRISGL	23	49	4
rChymotrypsin	H4_RAT	TEHAKRKTVTAMDVVY	73	88	4
rChymotrypsin	H4_RAT	TEHAKRKTVTAM[15.9949]DVVY	73	88	4

Table S5: Per-Sample Reagent and Researcher Time Cost Comparison (5 µg Histone Peptide Input)*

Workflow	Reagent/Sample	Prep Time/Batch	Researcher/Sample	MS Runs/Sample	Total/Sample	Total/100 Samples
Trypsin + propionylation	$0.70	6–12 h	$24.00–$48.00	1	**$24.70–$48.70**	**$2,470–$4,870**
Arg-C Ultra + propionylation	$1.34	~6 h	$24.00	1	**$25.34**	**$2,534**
Arg-C Ultra (unlabeled)	$1.33	~2 h	$8.00	1	**$9.33**	**$933**
Arg-C Ultra + TMTzero	$4.58	~3 h	$12.00	1	**$16.58**	**$1,658**
Arg-C Ultra + TMT10plex	$131.50	~3 h	$12.00	0.1	**$143.50**	**$14,350**
RIPUP + propionylation	$1.34+	~6 h	$24.00	2	**$25.34+**	**$2,534+**

RIPUP (unlabeled)	$1.33+	~2 h	$8.00	2	**$9.33+**	**$933+**
RIPUP + TMTzero	$4.58+	~3 h	$12.00	2	**$16.58+**	**$1,658+**
RIPUP + TMT10plex	$131.50+	~3 h	$12.00	0.2	**$143.50+**	**$14,350+**

* All costs are reported in USD and exclude instrument acquisition time; the MS Runs/Sample column is provided so that readers may calculate instrument costs using local rates. Reagent costs were calculated from manufacturer list prices assuming 5 µg histone peptide input per sample. Enzyme prices: Trypsin Gold MS-grade (Promega, $138/100 µg) at a 1:10 enzyme-to-substrate (E:S) ratio ($0.69/sample); Arg-C Ultra (Promega, $133/5 µg) at a 1:100 E:S ratio ($1.33/sample). r-Chymotrypsin (Promega, Early Access program) pricing has not been established; RIPUP workflow totals are reported as additive to the Arg-C Ultra cost. TMTzero (Thermo Scientific, Cat. No. 90067, $325/5 x 0.8 mg) is costed for single-channel derivatization, with per-sample cost assuming proportional scaling of the manufacturer-recommended protocol (0.8 mg per 100 µg peptide) to 5 µg input (~20 samples per vial, ~100 samples per kit, $3.25/sample). TMT10plex (Thermo Scientific, Cat. No. 90110, $1,302/10 × 0.8 mg) is costed for standard 10-plex isobaric multiplexing ($130.17/sample); ten samples are combined per MS run. Propionic anhydride (Sigma-Aldrich, Cat. No. 240311, ≥99%, ~$30–50/100 mL) per-sample reagent cost is negligible (<$0.01). Researcher time was estimated at $40/h for batches of 10 samples processed in parallel, with per-sample cost calculated as (batch prep time ÷ 10) x $40. Propionylation workflows include two pre-digestion and two post-digestion derivatization rounds and enzymatic digestion; the trypsin-based workflow spans two calendar days due to overnight digestion (6–12 h range reflects variability in digestion time and batch complexity). Arg-C Ultra and RIPUP workflows without chemical derivatization (~2 h) include digestion; RIPUP runs Arg-C Ultra and r-Chymotrypsin digestions in parallel on separate aliquots. TMT labeling adds ~1 h (reagent reconstitution, incubation, hydroxylamine quench). Non-multiplexed workflows require one MS run per sample per protease (1 for single protease, 2 for RIPUP dual protease). TMT10plex workflows combine 10 samples per run (0.1 for single protease, 0.2 for RIPUP). All reagent prices reflect manufacturer list prices as of mid-2026 and may vary with institutional contracts or volume discounts. Costs do not include instrument acquisition time, consumables (e.g., desalting tips, LC columns), or sample extraction.
