# Supplementary material for: Rapid Histone Post-Translational Modification Analysis Using Alternative Proteases and Tandem Mass Tags
Source: bioRxiv. 2026 Feb 22:2026.02.13.705817. Originally published 2026 Feb 15. Preprint. [Version 2] doi: 10.64898/2026.02.13.705817 (PMC12918826; doi:10.64898/2026.02.13.705817)
Supplement: Supplementary file 1 [file media-1.pdf]

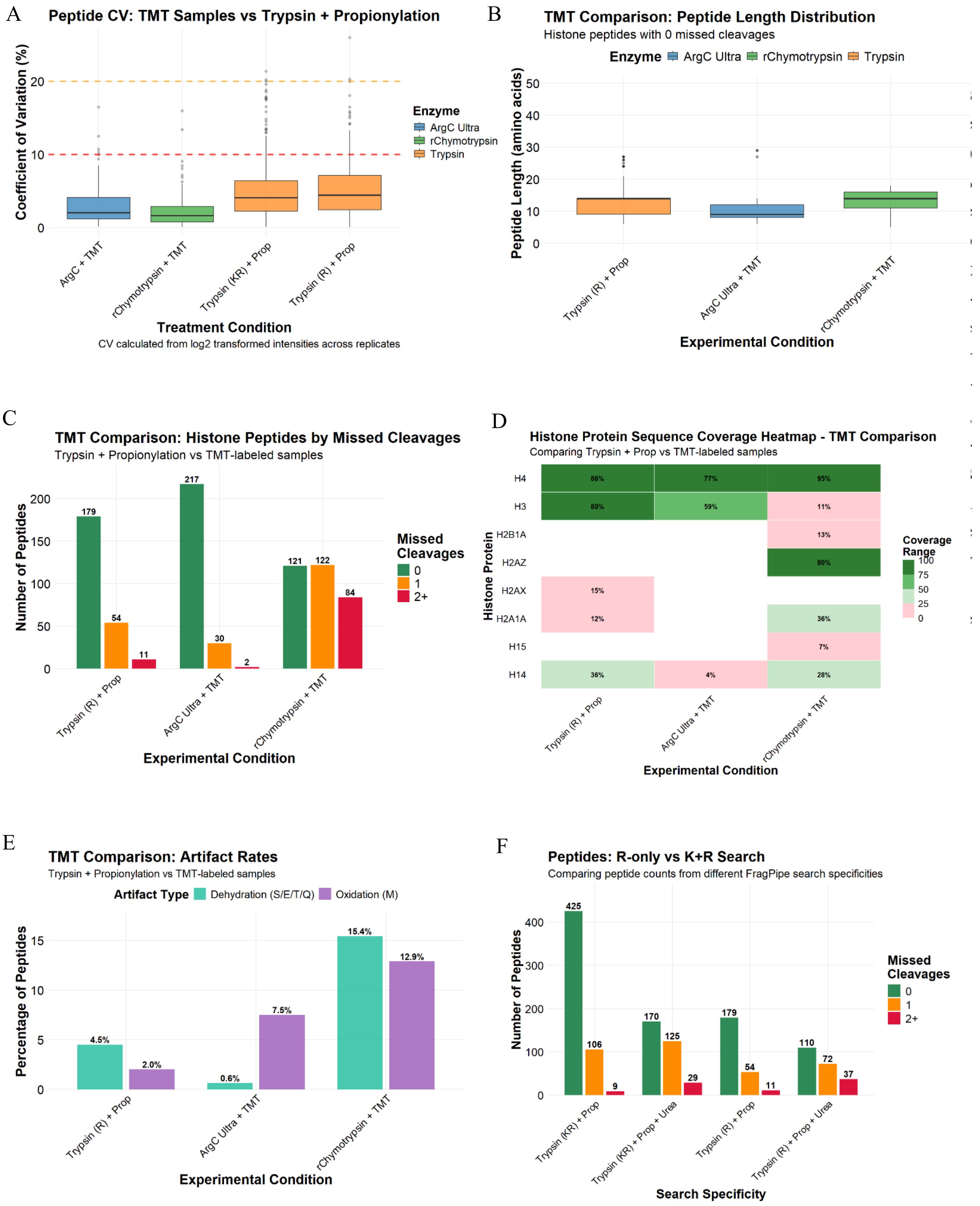
A
Peptide CV: TMT Samples vs Trypsin + Propionylation
Coefficient of Variation (%)
Enzyme
ArgC Ultra
rChymotrypsin
Trypsin
ArgC + TMT
rChymotrypsin + TMT
Trypsin (KR) + Prop
Trypsin (R) + Prop
Treatment Condition
CV calculated from log2 transformed intensities across replicates
B
TMT Comparison: Peptide Length Distribution
Histone peptides with 0 missed cleavages
Enzyme ArgC Ultra rChymotrypsin Trypsin
Peptide Length (amino acids)
Trypsin (R) + Prop
ArgC Ultra + TMT
rChymotrypsin + TMT
Experimental Condition
C
TMT Comparison: Histone Peptides by Missed Cleavages
Trypsin + Propionylation vs TMT-labeled samples
Number of Peptides
Missed Cleavages
0
1
2+
179
54
11
217
30
2
121
122
84
Trypsin (R) + Prop
ArgC Ultra + TMT
rChymotrypsin + TMT
Experimental Condition
D
Histone Protein Sequence Coverage Heatmap - TMT Comparison
Comparing Trypsin + Prop vs TMT-labeled samples
Histone Protein
H4 86% 77% 95%
H3 80% 59% 11%
H2B1A 13%
H2AZ 80%
H2AX 15%
H2A1A 12% 36%
H15 7%
H14 36% 4% 28%
Coverage Range
100
75
50
25
0
Trypsin (R) + Prop
ArgC Ultra + TMT
rChymotrypsin + TMT
Experimental Condition
E
TMT Comparison: Artifact Rates
Trypsin + Propionylation vs TMT-labeled samples
Artifact Type Dehydration (S/E/T/Q) Oxidation (M)
Percentage of Peptides
4.5%
2.0%
0.6%
7.5%
15.4%
12.9%
Trypsin (R) + Prop
ArgC Ultra + TMT
rChymotrypsin + TMT
Experimental Condition
F
Peptides: R-only vs K+R Search
Comparing peptide counts from different FragPipe search specificities
Number of Peptides
Missed Cleavages
0
1
2+
425
106
9
170
125
29
179
54
11
110
72
37
Trypsin (KR) + Prop
Trypsin (KR) + Prop + Urea
Trypsin (R) + Prop
Trypsin (R) + Prop + Urea
Search Specificity

H1.4 Succinylation sites: sequence coverage

Protease-specific site coverage

r-Chymotrypsin + TMT

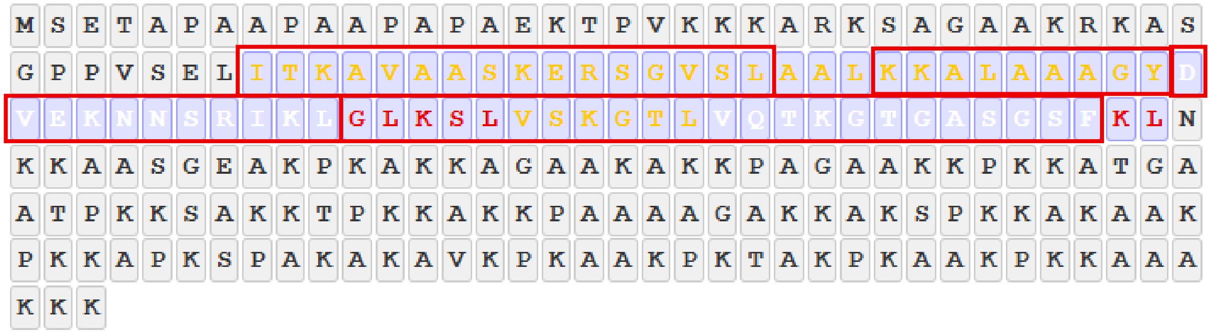

ITkAVAASKERSGVSL
ITKAVAASkERSGVSL
kKALAAAGY
KkALAAAGY
DVEkNNSRIKL
DVEKNNSRIkL
GLkSLVSKGTL
GLKSLVSkGTL; VSkGTL; VSkGTLVQTKGTGASGSF
VQTkGTGASGSF; VSKGTLVQTkGTGASGSF

Arg-C Ultra + TMT

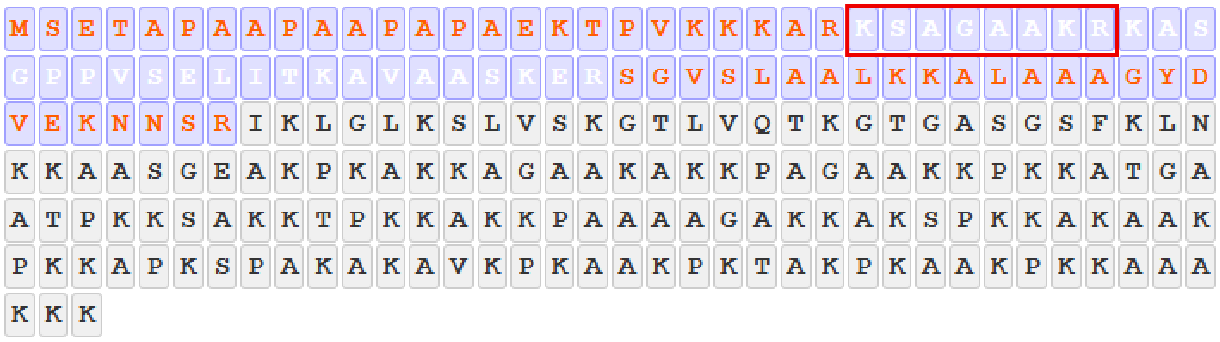

kSAGAAKR
KSAGAAkR

Figure S2: H1.4 sequence coverage and succinylation sites in r-Chymotrypsin + TMT (top) and Arg-C Ultra + TMT (bottom) samples. Theoretical sequence coverage is indicated by purple shading. Red boxes indicate observed peptides/sequence coverage of succinyl-K peptides. Peptides highlighted in yellow indicate protease-specific succinyl-K site coverage.

Histone H4 Succinylation sites: sequence coverage

Protease-specific site coverage

Succinylation sites identified by both Arg-C Ultra and r-Chymotrypsin

r-Chymotrypsin + TMT

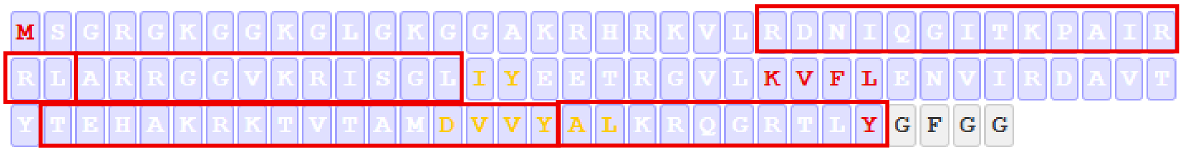

RDNIQGITkPAIRRL
ARRGGVkRISGL
TEHAKRkTVTAM;TEHAKRkTVTAMDVVY
ALkRQGRTLY

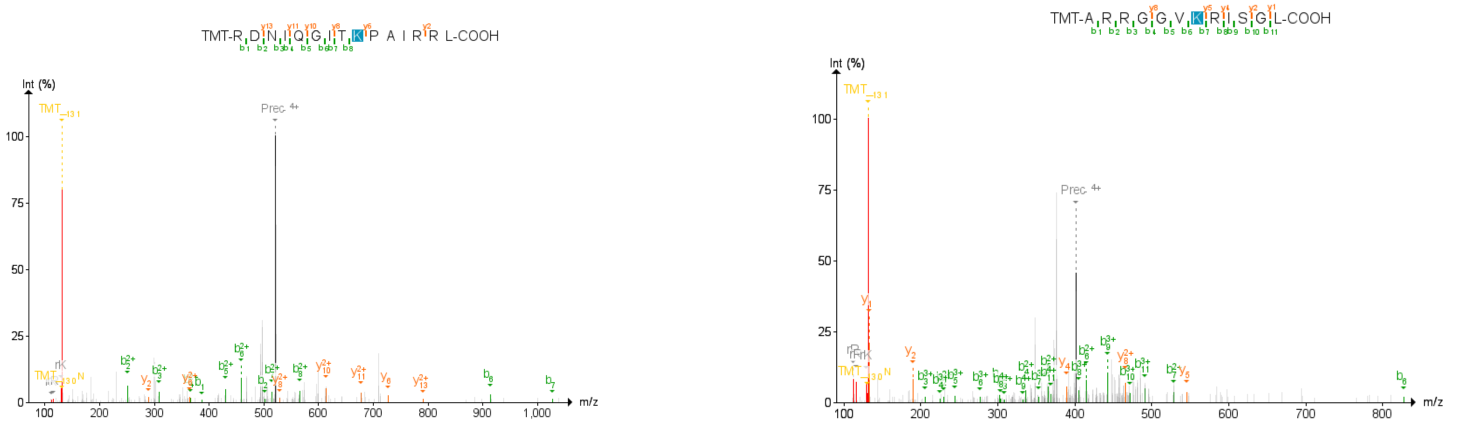

Figure S3: H4 sequence coverage and succinylation sites in r-Chymotrypsin + TMT samples. Top panels: Theoretical sequence coverage is indicated by purple shading. Red boxes indicate observed peptides/sequence coverage of succinyl-K peptides. Peptides highlighted in yellow indicate protease-specific K-succinyl site coverage. Green highlighted K residues indicate succinyl-K sites identified by both r-Chymotrypsin + TMT and Arg-C Ultra + TMT. Bottom panels: Example fragment spectra of RDNIQGITkPAIRRL (left) and ARRGGVkRISGL peptides showing increased *b*-ion series and flanking fragment ions of succinyl-K sites. Succinyl-K sites are highlighted in color.

Arg-C Ultra + TMT

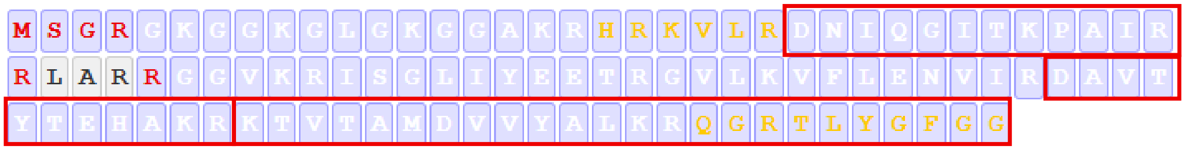

DNIQGITkPAIR; DNIQGITkPAIRR; KVLRDNIQGITkPAIR

DAVTYTEHAkR

kTVTAMDVVYALKR; kTVTAMDVVYALKRQGR; kTVTAMDVVYALKRQGRTLYGFGG

KTVTAMDVVYALkR

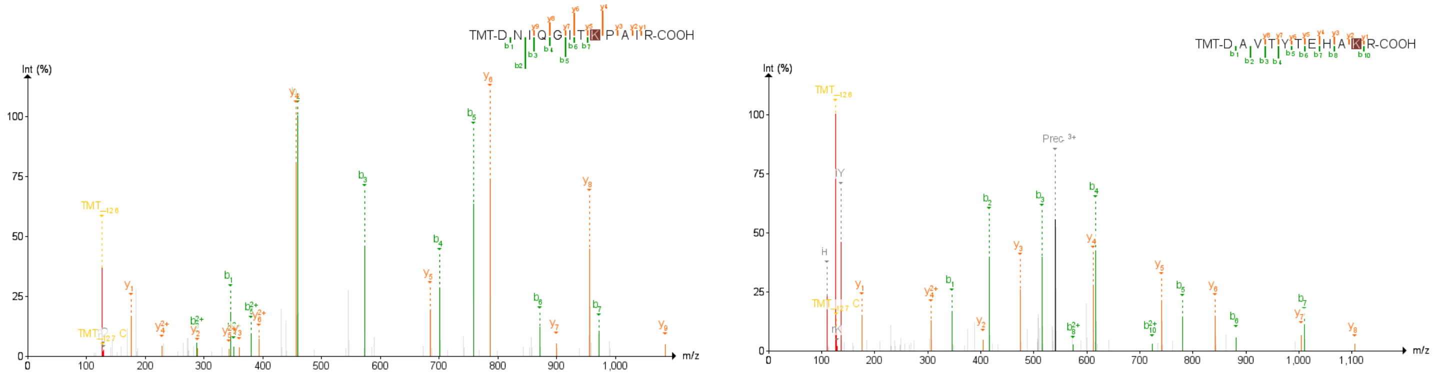

Figure S4: H4 sequence coverage and succinylation sites in Arg-C Ultra + TMT samples. Top panels: Theoretical sequence coverage is indicated by purple shading. Red boxes indicate observed peptides/sequence coverage of succinyl-K peptides. Peptides highlighted in yellow indicate protease-specific K-succinyl site coverage. Green highlighted K residues indicate succinyl-K sites identified by both r-Chymotrypsin + TMT and Arg-C Ultra + TMT. Bottom panels: Annotated HCD MS/MS spectra of DNIQGITkPAIR and DAVTYTEHAkR confirming succinyl-K localization. The modification site is unambiguously assigned based on continuous *b*-ion and/or *y*-ion series flanking the modified lysine residue (highlighted). Precursor mass tolerance was within 10 ppm and fragment mass tolerance was within 20 ppm.

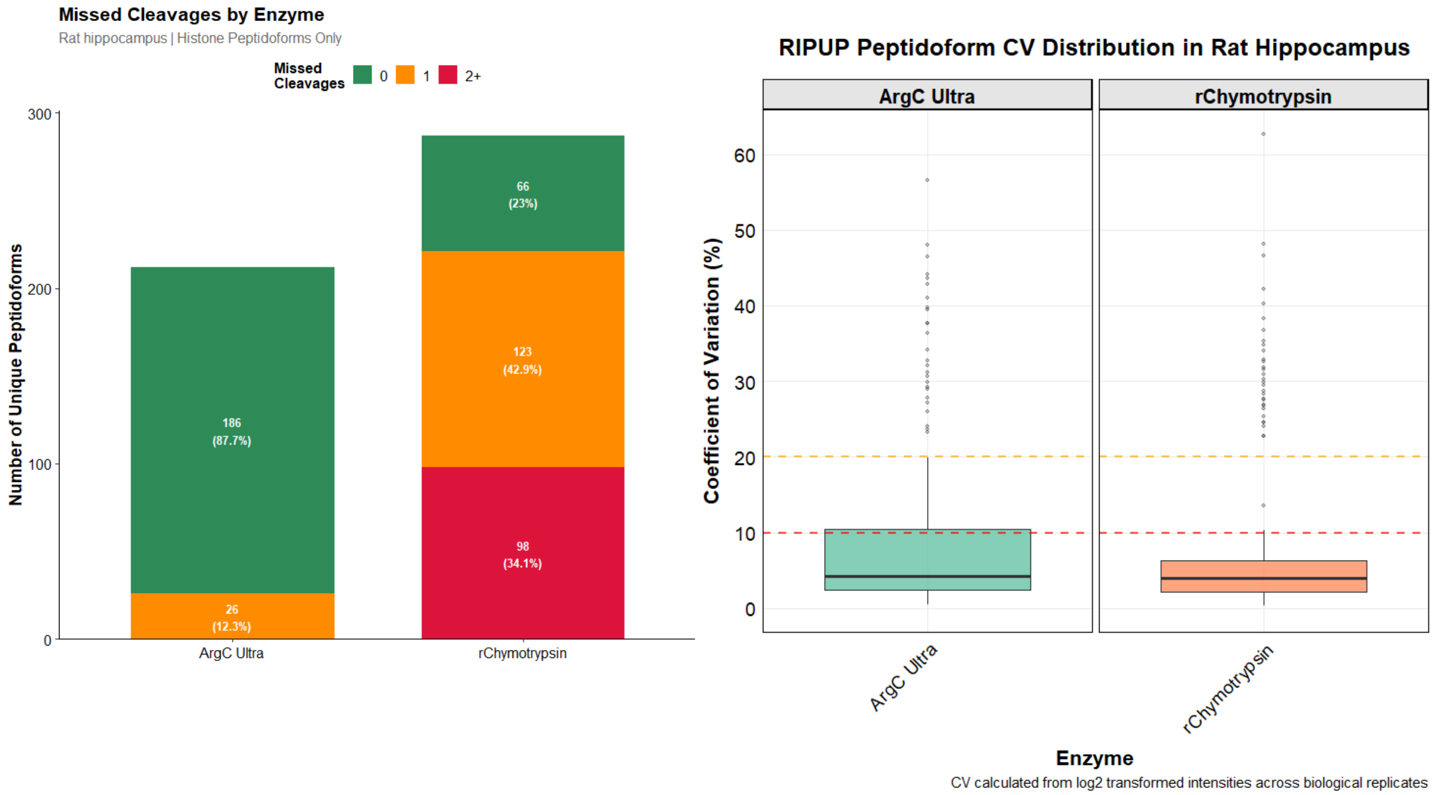

Figure S5: (Left) Unique peptidoforms and missed cleavages in histone extracts from rat hippocampus digested with Arg-C Ultra (1:10; left) and r-Chymotrypsin (1:10; right). (Right) CV distributions of peptidoforms identified with the RIPUP protocol in rat hippocampal sections.

Table S1: Histone peptides containing succinylation on lysine identified with Arg-C Ultra + TMT.*

Gene	Peptide
H2AC4	GKQGGkAR
H2AC4	AkAKTR
H2AC4	AKAkTR
H2AC4	kGNYSER
H2AC4	GkQGGKAR
H2AC4	DNkKTR
H2AC4	DNKkTR
H2AC4	NDEELNkLLGR
H1-4	kSAGAAKR
H1-4	KSAGAAkR
H2BC3	LAHYNkR
H4C1	DNIQGITkPAIR;DNIQGITkPAIRR;KVLRDNIQGITkPAIR
H4C1	DAVTYTEHAkR
H4C1	kTVTAMDVVYALKR;kTVTAMDVVYALKRQGR;kTVTAMDVVYALKRQGRTLYGFGG
H4C1	KTVTAMDVVYALkR
H3-4	EIAQDFkTDLR
H2AC20	kGNYAER
H3-2	kSTGGKAPR
H3-2	KSTGGkAPR
H3-2	kQLATKAAR
H3-2	KQLATkAAR
H3-2	kSAPATGGVKKPHR
H3-2	KSAPATGGVkKPHR
H3-2	KSAPATGGVKkPHR
H3-2	TkQTAR
H3-2	YQkSTELLIR
H3-2	kLPFQR
H3-5	VTIMPkDIQLAR

*Lowercase ‘k’ indicates succinylation site.
