# Supplementary material for: Rapid Histone Post-Translational Modification Analysis Using Alternative Proteases and Tandem Mass Tags
Source: bioRxiv. 2026 Feb 22:2026.02.13.705817. Originally published 2026 Feb 15. Preprint. [Version 2] doi: 10.64898/2026.02.13.705817 (PMC12918826; doi:10.64898/2026.02.13.705817)
Supplement: Supplementary file 2 [file media-2.pdf]

Supplementary File 2

SDS-PAGE images of histones extracted from HEK293T and Rat hippocampal nuclei.

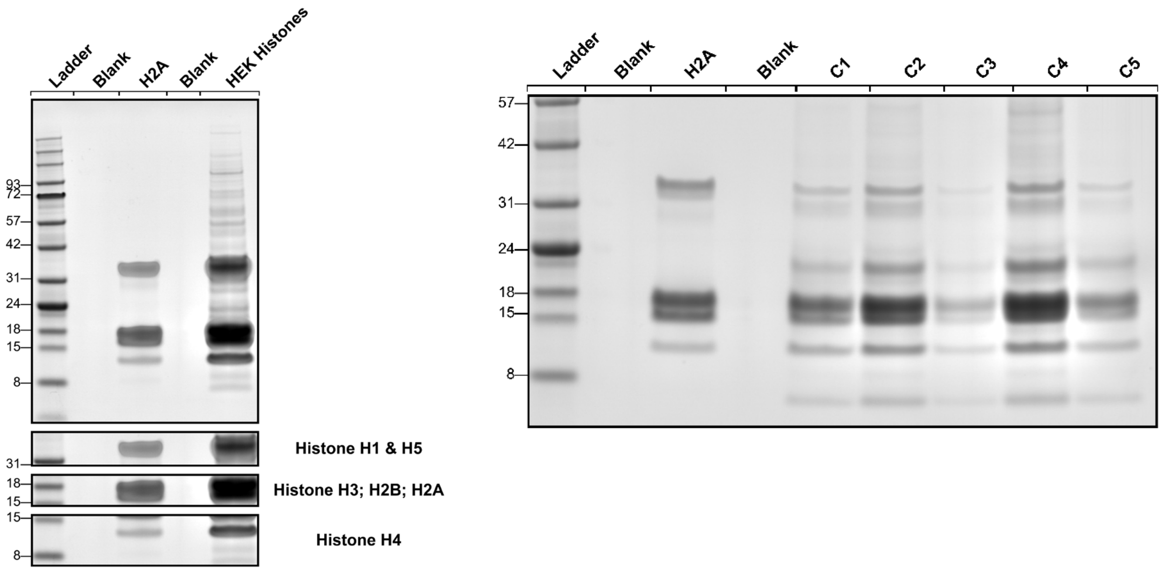

Figure S1: SDS-PAGE protein profiles of histones extracted from HEK293T cells (left) and rat hippocampal sections (right). Five µL of extracted histones or 2 µg of histone standard were resolved on a 4-12% Bis-Tris mini-protein gel and subsequently stained to visualize histone protein bands and confirm sample integrity. Blank = no sample; C1-5 = histones extracted from rat hippocampal sections, biological replicates 1 – 5; H2A = Histone type IIA from calf thymus (catalog number H9250, Sigma-Aldrich); HEK Histones = histones extracted from HEK293T nuclei.
