# Supplementary material for: Rapid Histone Post-Translational Modification Analysis Using Alternative Proteases and Tandem Mass Tags
Source: bioRxiv. 2026 Feb 22:2026.02.13.705817. Originally published 2026 Feb 15. Preprint. [Version 2] doi: 10.64898/2026.02.13.705817 (PMC12918826; doi:10.64898/2026.02.13.705817)
Supplement: Supplementary file 3 [file media-3.pdf]

Dataset_So	Protein	Modified Se	Start	End	Peptidoforr	n_replicate	mean_inter	log2_mean_	sd_log2_int	cv_percent
ArgC Ultra	H10_RAT	AKAAKKSTD	15	41	H10_RAT_A	4	0.003807	-8.13816	0.629097	7.730211
ArgC Ultra	H10_RAT	LVTTGVLKQ	74	93	H10_RAT_L	4	0.006723	-7.22467	0.174786	2.419298
ArgC Ultra	H10_RAT	QSIQKYIKS	47	73	H10_RAT_Q	4	0.001384	-9.64395	0.778972	8.077311
ArgC Ultra	H10_RAT	TENSTSTPA	1	14	H10_RAT_T	4	5.68E-05	-14.1399	0.363816	2.572983
ArgC Ultra	H10_RAT	n[42.0106]T	1	14	H10_RAT_n	4	0.001249	-9.72976	0.561287	5.768761
ArgC Ultra	H11_RAT	KKPAGPSVS	34	55	H11_RAT_K	4	9.88E-05	-13.39	0.598052	4.466413
ArgC Ultra	H11_RAT	SGVSLAALK	56	80	H11_RAT_S	4	1.08E-04	-13.2589	0.603604	4.552424
ArgC Ultra	H11_RAT	SGVSLAALK	56	80	H11_RAT_S	3	3.34E-05	-14.9175	0.441259	2.957999
ArgC Ultra	H11_RAT	n[42.0106]S	1	24	H11_RAT_n	4	7.11E-05	-13.8296	0.425442	3.0763
ArgC Ultra	H14_RAT	AVKPKAAKK	192	218	H14_RAT_A	4	2.88E-04	-11.8475	0.586411	4.949673
ArgC Ultra	H14_RAT	KASGPPVSE	33	53	H14_RAT_K	4	0.020073	-5.68145	0.413759	7.282626
ArgC Ultra	H14_RAT	KASGPPVSE	33	53	H14_RAT_K	3	1.05E-05	-17.2659	2.171615	12.57747
ArgC Ultra	H14_RAT	KAS[79.966	33	53	H14_RAT_K	4	2.15E-05	-15.5059	0.096996	0.625546
ArgC Ultra	H14_RAT	K[42.0106]A	33	53	H14_RAT_K	4	1.15E-05	-16.4299	0.31691	1.928865
ArgC Ultra	H14_RAT	SETAPAAPA	1	24	H14_RAT_S	4	5.18E-05	-14.2474	0.190258	1.335383
ArgC Ultra	H14_RAT	SGVSLAALK	54	78	H14_RAT_S	4	0.013847	-6.22422	0.440385	7.075351
ArgC Ultra	H14_RAT	n[42.0106]S	1	24	H14_RAT_n	4	0.007415	-7.08252	0.166148	2.345886
ArgC Ultra	H14_RAT	n[42.0106]S	1	24	H14_RAT_n	4	1.74E-05	-15.8226	0.210906	1.33294
ArgC Ultra	H14_RAT	n[42.0106]S	1	24	H14_RAT_n	4	3.23E-05	-14.9662	0.431334	2.882064
ArgC Ultra	H14_RAT	n[42.0106]S	1	24	H14_RAT_n	3	2.01E-05	-15.6179	0.236503	1.514311
ArgC Ultra	H14_RAT	n[42.0106]S	1	24	H14_RAT_n	4	4.99E-05	-14.2945	0.12627	0.88335
ArgC Ultra	H14_RAT	n[42.0106]S	1	24	H14_RAT_n	4	4.36E-04	-11.1712	0.179802	1.609509
ArgC Ultra	H15_RAT	GGVSLPALK	53	77	H15_RAT_G	4	7.69E-04	-10.4124	0.532116	5.110417
ArgC Ultra	H15_RAT	KATGPPVSE	32	52	H15_RAT_K	3	9.93E-04	-9.97979	0.139199	1.394807
ArgC Ultra	H15_RAT	PKAVKSKAS	188	221	H15_RAT_P	4	3.29E-05	-15.0256	0.682223	4.540398
ArgC Ultra	H15_RAT	SETAPAETT	1	31	H15_RAT_S	4	1.37E-05	-16.1869	0.315447	1.948778
ArgC Ultra	H2A1C_RA	AGLQFPVG	21	29	H2A1C_RA	4	0.026013	-5.2735	0.184817	3.504632
ArgC Ultra	H2A1C_RA	HLQLAIR	82	88	H2A1C_RA	4	0.019952	-5.68246	0.367794	6.472444
ArgC Ultra	H2A1C_RA	NDEELNKLL	89	99	H2A1C_RA	4	0.008973	-6.80816	0.178026	2.614885
ArgC Ultra	H2A1C_RA	NDEELNK[2	89	99	H2A1C_RA	3	1.80E-05	-15.8089	0.467032	2.954236
ArgC Ultra	H2A1C_RA	VGAGAPVY	43	71	H2A1C_RA	3	2.42E-05	-15.3332	0.077435	0.505018
ArgC Ultra	H2A1C_RA	VTIAQGGVL	100	129	H2A1C_RA	4	0.010013	-6.70393	0.504986	7.53269
ArgC Ultra	H2A1C_RA	VTIAQGGVL	100	129	H2A1C_RA	4	3.51E-04	-11.4846	0.19814	1.725272
ArgC Ultra	H2A1C_RA	VTIAQGGVL	100	129	H2A1C_RA	4	2.53E-04	-11.9661	0.260526	2.177196
ArgC Ultra	H2A1C_RA	VTIAQGGVL	100	129	H2A1C_RA	3	2.20E-04	-12.1537	0.166154	1.367101
ArgC Ultra	H2A1C_RA	VTIAQGGVL	100	129	H2A1C_RA	4	3.26E-04	-11.8751	1.214599	10.22815
ArgC Ultra	H2A1C_RA	VTIAQGGVL	100	129	H2A1C_RA	3	4.57E-05	-14.4382	0.294863	2.042246
ArgC Ultra	H2A1C_RA	VTIAQGGVL	100	129	H2A1C_RA	4	2.60E-04	-12.2895	1.456279	11.84977
ArgC Ultra	H2A1C_RA	VTIAQGGVL	100	129	H2A1C_RA	3	2.90E-05	-15.1097	0.381341	2.523806
ArgC Ultra	H2A1C_RA	VTIAQGGVL	100	129	H2A1C_RA	4	4.23E-04	-11.2146	0.184733	1.647257
ArgC Ultra	H2A1C_RA	VTIAQGGVL	100	129	H2A1C_RA	4	2.44E-04	-12.0193	0.268311	2.23233
ArgC Ultra	H2A1C_RA	VTIAQGGVL	100	129	H2A1C_RA	3	9.94E-05	-13.3631	0.526157	3.937386
ArgC Ultra	H2A1F_RA	VTIAQGGVL	100	129	H2A1F_RA	4	5.23E-04	-10.9485	0.432976	3.954654
ArgC Ultra	H2A1F_RA	VTIAQGGVL	100	129	H2A1F_RA	4	1.25E-05	-16.3217	0.388201	2.378438
ArgC Ultra	H2A1F_RA	VTIAQGGVL	100	129	H2A1F_RA	4	9.79E-06	-16.7074	0.533613	3.193864
ArgC Ultra	H2A1F_RA	VTIAQGGVL	100	129	H2A1F_RA	3	1.75E-04	-12.7136	1.064565	8.373454
ArgC Ultra	H2A1F_RA	VTIAQGGVL	100	129	H2A1F_RA	2	1.48E-04	-13.5976	2.481533	18.24977
ArgC Ultra	H2A2A_RA	NDEELNKLL	89	129	H2A2A_RA	4	0.007672	-7.11246	0.554396	7.794717
ArgC Ultra	H2A2A_RA	NDEELNKLL	89	129	H2A2A_RA	4	3.04E-04	-11.7484	0.509629	4.337858

ArgC Ultra	H2A2A_RAT	NDEELNKLI	89	129	H2A2A_RAT	4	1.96E-04	-12.4277	0.627893	5.052379
ArgC Ultra	H2A2A_RAT	NDEELNKLI	89	129	H2A2A_RAT	4	2.95E-04	-11.9189	0.884403	7.420187
ArgC Ultra	H2A2A_RAT	NDEELNKLI	89	129	H2A2A_RAT	2	8.22E-05	-14.0923	1.843157	13.07914
ArgC Ultra	H2A2A_RAT	NDEELNKLI	89	129	H2A2A_RAT	3	1.40E-04	-13.0298	1.023586	7.855707
ArgC Ultra	H2A2A_RAT	VGAGAPVY	43	71	H2A2A_RAT	3	1.18E-04	-13.0526	0.181127	1.387669
ArgC Ultra	H2A4_RAT	VTIAQGGVI	100	129	H2A4_RAT_	4	9.09E-06	-16.905	0.847628	5.014066
ArgC Ultra	H2A4_RAT	VTIAQGGVI	100	129	H2A4_RAT_	3	1.00E-05	-16.6119	0.1808	1.088381
ArgC Ultra	H2AJ_RAT	VTIAQGGVI	100	128	H2AJ_RAT_	4	0.00131	-9.60268	0.320403	3.336602
ArgC Ultra	H2AJ_RAT	VTIAQGGVI	100	128	H2AJ_RAT_	3	2.37E-04	-12.046	0.122199	1.014436
ArgC Ultra	H2AJ_RAT	VTIAQGGVI	100	128	H2AJ_RAT_	3	4.17E-04	-11.4097	0.867101	7.599664
ArgC Ultra	H2AJ_RAT	VTIAQGGVI	100	128	H2AJ_RAT_	3	1.59E-05	-16.2278	1.219001	7.511824
ArgC Ultra	H2AJ_RAT	VTIAQGGVI	100	128	H2AJ_RAT_	4	1.26E-05	-16.3879	0.656685	4.007128
ArgC Ultra	H2AJ_RAT	VTIAQGGVI	100	128	H2AJ_RAT_	4	3.10E-05	-15.154	0.919589	6.068282
ArgC Ultra	H2AJ_RAT	VTIAQGGVI	100	128	H2AJ_RAT_	4	1.97E-05	-15.7683	0.722914	4.584615
ArgC Ultra	H2AJ_RAT	VTIAQGGVI	100	128	H2AJ_RAT_	4	0.00581	-7.96969	1.475702	18.51642
ArgC Ultra	H2AY_RAT	KLKSIAFPSI	300	313	H2AY_RAT_	4	2.33E-04	-12.2276	0.791616	6.474002
ArgC Ultra	H2AY_RAT	SAKAGVIFP	15	26	H2AY_RAT_	4	2.22E-04	-12.3112	0.811271	6.589711
ArgC Ultra	H2AZ_RAT	AGGKAGK[4	1	19	H2AZ_RAT_	3	1.67E-05	-17.4367	3.714297	21.30159
ArgC Ultra	H2AZ_RAT	AGGKAGK[4	1	19	H2AZ_RAT_	4	1.10E-05	-18.3054	3.379745	18.46315
ArgC Ultra	H2AZ_RAT	AGGK[42.0	1	19	H2AZ_RAT_	4	2.96E-05	-15.0798	0.359859	2.386367
ArgC Ultra	H2AZ_RAT	AGGK[42.0	1	19	H2AZ_RAT_	4	1.91E-05	-15.7423	0.516029	3.277971
ArgC Ultra	H2AZ_RAT	GDEELDSLI	92	127	H2AZ_RAT_	4	4.88E-04	-11.0317	0.352882	3.198802
ArgC Ultra	H2AZ_RAT	GDEELDSLI	92	127	H2AZ_RAT_	4	3.27E-05	-14.9669	0.537305	3.589949
ArgC Ultra	H2AZ_RAT	GDEELDSLI	92	127	H2AZ_RAT_	3	3.60E-05	-14.7803	0.280598	1.898457
ArgC Ultra	H2AZ_RAT	GDEELDSLI	92	127	H2AZ_RAT_	2	4.24E-05	-14.5516	0.390291	2.682111
ArgC Ultra	H2B1_RAT	EIQTAVR	92	98	H2B1_RAT_	4	0.016777	-5.91142	0.237593	4.019225
ArgC Ultra	H2B1_RAT	KESYSVYVY	34	72	H2B1_RAT_	3	0.004761	-8.47467	2.133192	25.17139
ArgC Ultra	H2B1_RAT	K[14.0156]	34	72	H2B1_RAT_	3	3.16E-04	-11.6429	0.257757	2.213851
ArgC Ultra	H2B1_RAT	LLLPGELAK	99	124	H2B1_RAT_	4	0.023923	-5.4792	0.6197	11.31005
ArgC Ultra	H2B1_RAT	LLLPGELAK	99	124	H2B1_RAT_	4	2.51E-05	-15.3463	0.498999	3.251589
ArgC Ultra	H2B1_RAT	LLLPGELAK	99	124	H2B1_RAT_	4	1.59E-04	-12.6683	0.43158	3.406758
ArgC Ultra	H2B1_RAT	LLLPGELAK	99	124	H2B1_RAT_	3	2.01E-04	-12.3133	0.375908	3.052863
ArgC Ultra	H2B1_RAT	LLLPGELAK	99	124	H2B1_RAT_	4	2.30E-05	-15.6451	0.994859	6.358921
ArgC Ultra	H2B1_RAT	PEPAKSR[3	1	29	H2B1_RAT_	3	9.62E-05	-13.3477	0.140236	1.05064
ArgC Ultra	H31_RAT	K[28.0313]	27	40	H31_RAT_K	4	5.09E-05	-14.2826	0.279175	1.954651
ArgC Ultra	H31_RAT	K[28.0313]	27	40	H31_RAT_K	4	6.13E-05	-14.0007	0.157847	1.127419
ArgC Ultra	H31_RAT	K[28.0313]	27	40	H31_RAT_K	3	1.32E-04	-12.9218	0.393104	3.042162
ArgC Ultra	H31_RAT	K[42.0470]	27	40	H31_RAT_K	4	6.11E-05	-14.0076	0.180122	1.285886
ArgC Ultra	H31_RAT	K[42.0470]	27	40	H31_RAT_K	4	3.37E-05	-14.8785	0.282831	1.900942
ArgC Ultra	H33_RAT	EIAQDFKTD	73	83	H33_RAT_E	4	0.021298	-5.57736	0.30266	5.426576
ArgC Ultra	H33_RAT	EIAQDFK[14	73	83	H33_RAT_E	4	0.008479	-6.91408	0.349587	5.056163
ArgC Ultra	H33_RAT	EIAQDFK[28	73	83	H33_RAT_E	4	0.002103	-8.89489	0.078304	0.88032
ArgC Ultra	H33_RAT	KLPFQR	64	69	H33_RAT_K	4	0.009686	-6.7378	0.438565	6.509025
ArgC Ultra	H33_RAT	KQLATK[42	18	26	H33_RAT_K	4	0.001402	-9.47922	0.042292	0.446158
ArgC Ultra	H33_RAT	KSAPSTGGY	27	40	H33_RAT_K	4	3.59E-06	-18.1013	0.242955	1.342193
ArgC Ultra	H33_RAT	KSAPSTGGY	27	40	H33_RAT_K	4	1.82E-05	-15.7516	0.160451	1.018635
ArgC Ultra	H33_RAT	KSAPSTGGY	27	40	H33_RAT_K	4	7.81E-05	-13.7391	0.579587	4.218521
ArgC Ultra	H33_RAT	K[14.0156]	27	40	H33_RAT_K	4	1.85E-05	-15.7302	0.146447	0.930987
ArgC Ultra	H33_RAT	K[14.0156]	27	40	H33_RAT_K	4	6.47E-05	-13.9876	0.523221	3.740601
ArgC Ultra	H33_RAT	K[14.0156]	27	40	H33_RAT_K	3	2.56E-05	-15.2626	0.212991	1.395507

ArgC Ultra	H33_RAT	K[28.0313]	27	40	H33_RAT_K	4	7.83E-05	-13.7351	0.578877	4.214587
ArgC Ultra	H33_RAT	K[28.0313]	27	40	H33_RAT_K	4	0.001754	-9.23527	0.560328	6.067256
ArgC Ultra	H33_RAT	K[42.0106]	18	26	H33_RAT_K	4	3.11E-04	-11.6574	0.151552	1.30005
ArgC Ultra	H33_RAT	K[42.0106]	18	26	H33_RAT_K	4	6.41E-04	-10.6474	0.380832	3.576755
ArgC Ultra	H33_RAT	K[42.0106]	27	40	H33_RAT_K	3	8.69E-06	-16.8438	0.368111	2.185435
ArgC Ultra	H33_RAT	K[42.0106]	9	17	H33_RAT_K	4	3.05E-04	-11.6939	0.246861	2.111025
ArgC Ultra	H33_RAT	K[42.0470]	27	40	H33_RAT_K	4	0.001612	-9.30626	0.342302	3.678196
ArgC Ultra	H33_RAT	K[42.0470]	27	40	H33_RAT_K	4	5.85E-04	-10.7466	0.164533	1.531024
ArgC Ultra	H33_RAT	VTIMPKDIQ	117	128	H33_RAT_V	4	0.021834	-5.5254	0.179345	3.24583
ArgC Ultra	H33_RAT	YQKSTELLII	54	63	H33_RAT_Y	4	0.032751	-4.93789	0.144381	2.923943
ArgC Ultra	H4_RAT	DAVTYTEH/	68	78	H4_RAT_DA	4	0.045915	-4.48206	0.388633	8.670865
ArgC Ultra	H4_RAT	DNIQGITKP	24	35	H4_RAT_DN	4	0.035995	-4.80696	0.20279	4.218677
ArgC Ultra	H4_RAT	GKGGKGLG	4	17	H4_RAT_GK	4	5.10E-04	-10.9874	0.454791	4.139203
ArgC Ultra	H4_RAT	GKGGK[42.	4	17	H4_RAT_GK	4	3.80E-04	-11.4538	0.59841	5.224569
ArgC Ultra	H4_RAT	GKGGK[42.	4	17	H4_RAT_GK	4	3.15E-05	-16.1639	3.219504	19.91787
ArgC Ultra	H4_RAT	GKGGK[42.	4	17	H4_RAT_GK	4	1.63E-05	-15.9822	0.552994	3.460063
ArgC Ultra	H4_RAT	GK[42.0106	4	17	H4_RAT_GK	4	1.96E-04	-12.3416	0.319022	2.584935
ArgC Ultra	H4_RAT	GK[42.0106	4	17	H4_RAT_GK	4	3.61E-04	-11.4695	0.358306	3.123992
ArgC Ultra	H4_RAT	GK[42.0106	4	17	H4_RAT_GK	4	1.35E-04	-12.8737	0.251361	1.952507
ArgC Ultra	H4_RAT	GK[42.0106	4	17	H4_RAT_GK	4	3.64E-05	-14.7709	0.319095	2.160292
ArgC Ultra	H4_RAT	GK[42.0106	4	17	H4_RAT_GK	4	1.54E-04	-12.7188	0.467054	3.672151
ArgC Ultra	H4_RAT	GVLKVFLEN	56	67	H4_RAT_GV	4	0.007268	-7.37184	1.049832	14.24112
ArgC Ultra	H4_RAT	GVLK[162.0	56	67	H4_RAT_GV	4	1.76E-05	-15.9613	0.815578	5.109727
ArgC Ultra	H4_RAT	ISGLIYEETF	46	55	H4_RAT_ISG	4	0.029762	-5.08238	0.215591	4.241921
ArgC Ultra	H4_RAT	IS[27.9949]	46	55	H4_RAT_IS[	4	1.75E-05	-15.8327	0.334674	2.113817
ArgC Ultra	H4_RAT	IS[79.9663]	46	55	H4_RAT_IS[	4	1.26E-05	-16.3035	0.313487	1.922825
ArgC Ultra	H4_RAT	KTVTAMDV	79	92	H4_RAT_KT	4	0.02505	-5.38938	0.528448	9.805343
ArgC Ultra	H4_RAT	KTVTAMDV	79	92	H4_RAT_KT	4	3.48E-05	-14.8463	0.376894	2.538633
ArgC Ultra	H4_RAT	KT[27.9949	79	92	H4_RAT_KT	4	4.55E-06	-17.7482	0.095389	0.537455
ArgC Ultra	H4_RAT	K[162.0528	79	92	H4_RAT_K[1	4	2.26E-05	-15.4381	0.152709	0.989166
ArgC Ultra	H4_RAT	K[42.0106]T	79	92	H4_RAT_K[4	4	1.83E-05	-15.8193	0.600104	3.793504
ArgC Ultra	H4_RAT	SGR[14.015	1	17	H4_RAT_SG	4	2.39E-05	-16.1097	2.300506	14.28022
ArgC Ultra	H4_RAT	n[42.0106]	1	17	H4_RAT_n[4	4	1.74E-04	-12.617	0.685802	5.435541
ArgC Ultra	H4_RAT	n[42.0106]	1	17	H4_RAT_n[4	4	4.80E-05	-14.3871	0.39309	2.732249
ArgC Ultra	H4_RAT	n[42.0106]	1	17	H4_RAT_n[4	4	4.92E-05	-14.3348	0.299098	2.086518
ArgC Ultra	H4_RAT	n[42.0106]	1	17	H4_RAT_n[4	4	4.61E-05	-14.4122	0.177435	1.231145
rChymotryp	H10_RAT	AKGDEPKR	95	106	H10_RAT_A	4	8.35E-06	-16.8814	0.212336	1.257807
rChymotryp	H10_RAT	KQTKGVGA	81	92	H10_RAT_K	4	6.91E-04	-10.5195	0.279509	2.657062
rChymotryp	H10_RAT	KQTKGVGA	81	92	H10_RAT_K	4	2.83E-05	-15.1418	0.336109	2.219739
rChymotryp	H10_RAT	KVGENADS	58	69	H10_RAT_K	4	0.003213	-8.30339	0.280021	3.372374
rChymotryp	H10_RAT	KVGENADS	58	80	H10_RAT_K	4	7.14E-05	-13.9061	0.678266	4.877473
rChymotryp	H10_RAT	KVGENADS	58	69	H10_RAT_K	4	4.53E-04	-12.6206	3.832449	30.36667
rChymotryp	H10_RAT	RLAKGDEP	93	106	H10_RAT_R	4	0.002654	-8.56288	0.143426	1.67497
rChymotryp	H10_RAT	SDMIVAAIQ	28	52	H10_RAT_S	4	0.002486	-8.71614	0.502363	5.763597
rChymotryp	H10_RAT	SDM[15.99	28	52	H10_RAT_S	4	0.002503	-8.66836	0.319629	3.68731
rChymotryp	H10_RAT	SDM[15.99	28	52	H10_RAT_S	2	8.23E-04	-10.2769	0.42202	4.106488
rChymotryp	H10_RAT	SDM[15.99	28	52	H10_RAT_S	3	4.07E-05	-14.6369	0.478904	3.271901
rChymotryp	H10_RAT	SIKRLVTTG	70	80	H10_RAT_S	4	0.001213	-9.69227	0.144444	1.490299
rChymotryp	H10_RAT	SIKRLVTTG	70	92	H10_RAT_S	4	0.004739	-7.73541	0.239502	3.096179
rChymotryp	H10_RAT	TENSTSTPA	1	27	H10_RAT_T	4	8.05E-05	-13.6427	0.400402	2.934917

rChymotryp	H10_RAT	VTTGVLKQT	75	92	H10_RAT_V	4	1.46E-04	-12.9567	1.009428	7.790784
rChymotryp	H11_RAT	KK[42.0106	64	72	H11_RAT_K	3	1.71E-05	-15.9294	0.68382	4.292821
rChymotryp	H11_RAT	KSLVNKGTI	86	106	H11_RAT_K	4	1.21E-05	-16.4418	0.647533	3.938344
rChymotryp	H14_RAT	AAAGYDVE	66	81	H14_RAT_A	4	1.15E-04	-13.0954	0.213722	1.632038
rChymotryp	H14_RAT	AAAGYDVE	66	83	H14_RAT_A	4	7.59E-05	-13.7187	0.36943	2.69289
rChymotryp	H14_RAT	AAAGYDVE	66	81	H14_RAT_A	3	6.36E-05	-13.9588	0.283246	2.029153
rChymotryp	H14_RAT	AALKKALAA	59	70	H14_RAT_A	4	1.63E-04	-12.6018	0.273412	2.169624
rChymotryp	H14_RAT	DVEKNNSR	71	81	H14_RAT_D	4	5.76E-04	-10.7742	0.218616	2.029064
rChymotryp	H14_RAT	DVEKNNSR	71	83	H14_RAT_D	4	0.00127	-9.67449	0.459578	4.750409
rChymotryp	H14_RAT	DVEKNNSR	71	86	H14_RAT_D	4	3.81E-05	-14.7017	0.301313	2.049506
rChymotryp	H14_RAT	GLKSLVSK(	82	92	H14_RAT_G	4	2.62E-05	-15.2693	0.448885	2.939786
rChymotryp	H14_RAT	ITKAVAASK	43	58	H14_RAT_IT	4	2.11E-04	-12.2609	0.44245	3.608627
rChymotryp	H14_RAT	ITKAVAASK	43	61	H14_RAT_IT	4	3.50E-04	-11.4829	0.064566	0.562279
rChymotryp	H14_RAT	ITKAVAASK	43	65	H14_RAT_IT	4	3.08E-05	-15.0477	0.495347	3.291848
rChymotryp	H14_RAT	KKALAAAGY	62	70	H14_RAT_K	4	5.83E-04	-10.7476	0.110232	1.02564
rChymotryp	H14_RAT	KKALAAAGY	62	81	H14_RAT_K	4	7.04E-04	-10.4857	0.230204	2.195411
rChymotryp	H14_RAT	KKALAAAGY	62	81	H14_RAT_K	2	6.50E-04	-10.5877	0.059448	0.561485
rChymotryp	H14_RAT	KKALAAAGY	62	81	H14_RAT_K	4	5.99E-04	-10.7108	0.140683	1.313464
rChymotryp	H14_RAT	KKALAAAGY	62	81	H14_RAT_K	4	5.49E-04	-10.8364	0.130405	1.203399
rChymotryp	H14_RAT	KSLVSKGTL	84	92	H14_RAT_K	4	3.79E-05	-14.6886	0.054457	0.370743
rChymotryp	H14_RAT	KSLVSKGTL	84	104	H14_RAT_K	4	0.002168	-8.86949	0.271625	3.062467
rChymotryp	H14_RAT	KSLVSKGTL	84	104	H14_RAT_K	3	4.45E-05	-14.4616	0.145926	1.009056
rChymotryp	H14_RAT	K[14.0156]:	84	104	H14_RAT_K	2	0.001051	-11.5026	3.632053	31.57584
rChymotryp	H14_RAT	K[14.0156]:	84	104	H14_RAT_K	3	0.001846	-9.09946	0.280657	3.084324
rChymotryp	H14_RAT	K[42.0106]:	84	104	H14_RAT_K	4	0.002521	-8.83976	0.900879	10.19121
rChymotryp	H14_RAT	K[70.0419]:	84	104	H14_RAT_K	3	3.35E-04	-11.6333	0.6382	5.485976
rChymotryp	H14_RAT	VQTKGTGA	93	104	H14_RAT_V	4	5.55E-04	-10.8194	0.109593	1.012936
rChymotryp	H14_RAT	VSKGTLVQT	87	104	H14_RAT_V	4	1.93E-04	-12.3407	0.10238	0.829613
rChymotryp	H15_RAT	AAGGYDVE	65	80	H15_RAT_A	4	1.85E-05	-15.723	0.13102	0.833299
rChymotryp	H15_RAT	AAGGYDVE	65	82	H15_RAT_A	4	3.17E-05	-15.1038	0.763519	5.055156
rChymotryp	H15_RAT	ITKAVSASK	42	60	H15_RAT_IT	4	3.38E-05	-14.981	0.719685	4.803986
rChymotryp	H15_RAT	ITKAVSASK	42	64	H15_RAT_IT	3	1.09E-05	-16.745	1.029034	6.14533
rChymotryp	H15_RAT	KKALAAGGT	61	80	H15_RAT_K	4	3.61E-05	-14.7682	0.213797	1.447682
rChymotryp	H15_RAT	KKALAAGGT	61	80	H15_RAT_K	2	8.01E-05	-13.677	0.640059	4.679821
rChymotryp	H15_RAT	KKALAAGGT	61	80	H15_RAT_K	4	7.55E-05	-13.8374	0.757802	5.476464
rChymotryp	H2A1F_RAT	LRKGNYSEI	34	50	H2A1F_RAT	4	3.30E-04	-11.6149	0.429408	3.697037
rChymotryp	H2A1F_RAT	RKGNYSER	35	50	H2A1F_RAT	4	0.001287	-9.64729	0.416964	4.322083
rChymotryp	H2A1F_RAT	RKGNYSER	35	51	H2A1F_RAT	4	1.92E-04	-12.4115	0.488414	3.935167
rChymotryp	H2A1F_RAT	SERVGAGA	40	50	H2A1F_RAT	4	0.01044	-6.58919	0.172305	2.614964
rChymotryp	H2A1F_RAT	SERVGAGA	40	51	H2A1F_RAT	4	0.00378	-8.04932	0.087411	1.085945
rChymotryp	H2A1F_RAT	SGRGKQGG	1	33	H2A1F_RAT	4	3.72E-04	-11.5235	0.683344	5.929987
rChymotryp	H2A1F_RAT	TAEILELAAI	59	83	H2A1F_RAT	4	3.07E-05	-15.0657	0.520133	3.452438
rChymotryp	H2A2A_RAT	AERVGAGA	40	50	H2A2A_RAT	4	0.030152	-5.06689	0.24543	4.843791
rChymotryp	H2A2A_RAT	AERVGAGA	40	51	H2A2A_RAT	4	8.16E-04	-10.3371	0.530411	5.131116
rChymotryp	H2A2A_RAT	AERVGAGA	40	51	H2A2A_RAT	4	5.82E-04	-10.7553	0.175292	1.629821
rChymotryp	H2A2A_RAT	AGNAARDN	66	83	H2A2A_RAT	4	7.41E-05	-13.9515	1.068266	7.656974
rChymotryp	H2A2A_RAT	AGNAARDN	66	85	H2A2A_RAT	4	0.001476	-9.41533	0.205678	2.184502
rChymotryp	H2A2A_RAT	AIRNDEEL	86	93	H2A2A_RAT	4	6.71E-05	-13.8895	0.317527	2.286095
rChymotryp	H2A2A_RAT	AIRNDEELN	86	96	H2A2A_RAT	4	5.38E-04	-10.8646	0.124865	1.149278
rChymotryp	H2A2A_RAT	AIRNDEELN	86	97	H2A2A_RAT	4	0.001913	-9.04166	0.206124	2.279713

rChymotryp	H2A2A_RAT	ELAGNAAR	64	83	H2A2A_RAT	4	0.001531	-9.49079	0.733408	7.72758
rChymotryp	H2A2A_RAT	ELAGNAAR	64	85	H2A2A_RAT	4	0.013756	-6.27854	0.573667	9.136946
rChymotryp	H2A2A_RAT	E[-18.0106	64	83	H2A2A_RAT	4	8.73E-05	-13.5676	0.565565	4.168505
rChymotryp	H2A2A_RAT	E[-18.0106	64	85	H2A2A_RAT	4	6.33E-04	-10.6836	0.478926	4.48281
rChymotryp	H2A2A_RAT	LRKGNYAEI	34	50	H2A2A_RAT	4	0.001885	-9.05331	0.094668	1.045669
rChymotryp	H2A2A_RAT	PNIQAVLLP	109	129	H2A2A_RAT	4	1.80E-04	-12.5405	0.60215	4.801655
rChymotryp	H2A2A_RAT	PVGRVHRL	26	33	H2A2A_RAT	4	5.72E-05	-14.1191	0.304028	2.153316
rChymotryp	H2A2A_RAT	PVGRVHRL	26	34	H2A2A_RAT	4	1.83E-04	-12.4546	0.391589	3.144129
rChymotryp	H2A2A_RAT	PVGRVHRL	26	39	H2A2A_RAT	4	2.22E-05	-15.865	1.512073	9.530888
rChymotryp	H2A2A_RAT	QFPVGRVH	24	33	H2A2A_RAT	4	0.001287	-9.60984	0.179254	1.86532
rChymotryp	H2A2A_RAT	QFPVGRVH	24	34	H2A2A_RAT	4	0.006191	-7.37494	0.384782	5.217431
rChymotryp	H2A2A_RAT	QLAIRNDEI	84	93	H2A2A_RAT	3	1.29E-05	-16.2751	0.35183	2.161766
rChymotryp	H2A2A_RAT	QLAIRNDEI	84	96	H2A2A_RAT	4	6.31E-05	-13.9988	0.410702	2.933833
rChymotryp	H2A2A_RAT	RKGNYAER	35	50	H2A2A_RAT	4	0.006383	-7.31568	0.312402	4.270306
rChymotryp	H2A2A_RAT	RKGNYAER	35	51	H2A2A_RAT	4	6.38E-05	-13.9909	0.451233	3.225191
rChymotryp	H2A2A_RAT	RKGNYAER	35	51	H2A2A_RAT	4	2.28E-05	-15.4411	0.262257	1.698439
rChymotryp	H2A2A_RAT	TAEILELAGI	59	83	H2A2A_RAT	4	0.003549	-8.14667	0.176981	2.172433
rChymotryp	H2A2A_RAT	TAEILELAGI	59	83	H2A2A_RAT	4	7.05E-05	-13.8118	0.273171	1.977805
rChymotryp	H2AJ_RAT	AERVGAGA	40	51	H2AJ_RAT_	4	0.002538	-8.6262	0.124908	1.448005
rChymotryp	H2AJ_RAT	RKGNYAER	35	51	H2AJ_RAT_	4	1.30E-04	-12.9375	0.347	2.682132
rChymotryp	H2AY_RAT	AVANDEELI	83	93	H2AY_RAT_	3	5.23E-05	-15.6408	2.479399	15.85215
rChymotryp	H2AY_RAT	AVANDEELI	83	94	H2AY_RAT_	4	9.42E-05	-13.4307	0.447225	3.32987
rChymotryp	H2AY_RAT	ELAGNAAR	61	82	H2AY_RAT_	4	6.67E-05	-13.939	0.515283	3.696697
rChymotryp	H2AY_RAT	ELAGNAAR	61	82	H2AY_RAT_	4	0.00195	-9.02851	0.321425	3.560111
rChymotryp	H2AY_RAT	ELAGNAAR	61	82	H2AY_RAT_	4	2.22E-05	-15.6834	0.962974	6.140066
rChymotryp	H2AY_RAT	ILKAISSY	325	332	H2AY_RAT_	3	2.26E-05	-15.5402	0.713679	4.592466
rChymotryp	H2AY_RAT	KGVTIASGC	95	113	H2AY_RAT_	4	1.01E-04	-13.3385	0.499144	3.742139
rChymotryp	H2AY_RAT	M[15.9949]	48	54	H2AY_RAT_	2	9.59E-06	-16.6735	0.156137	0.936435
rChymotryp	H2AY_RAT	RIGVGAPVY	39	47	H2AY_RAT_	4	3.50E-04	-11.5267	0.429368	3.724989
rChymotryp	H2AY_RAT	RKKNGPLEY	244	267	H2AY_RAT_	4	3.24E-05	-14.9824	0.502453	3.353626
rChymotryp	H2AY_RAT	R[144.0423	244	267	H2AY_RAT_	4	3.73E-05	-14.7289	0.263794	1.790991
rChymotryp	H2AY_RAT	SSSIKTVY	338	345	H2AY_RAT_	3	2.41E-05	-15.4417	0.70308	4.553117
rChymotryp	H2AY_RAT	SSSIKTVYF	338	346	H2AY_RAT_	4	1.76E-04	-12.548	0.544451	4.338943
rChymotryp	H2AY_RAT	STKSLFL	185	191	H2AY_RAT_	3	4.03E-05	-14.7394	0.799323	5.423038
rChymotryp	H2AY_RAT	TAEILELAGI	56	81	H2AY_RAT_	3	1.59E-05	-16.0325	0.60512	3.774337
rChymotryp	H2AY_RAT	TAEILELAGI	56	81	H2AY_RAT_	4	1.61E-04	-13.4707	1.834461	13.61813
rChymotryp	H2AY_RAT	TAEILELAGI	56	81	H2AY_RAT_	4	0.005961	-7.46968	0.565155	7.565985
rChymotryp	H2AY_RAT	TAEILELAGI	56	81	H2AY_RAT_	4	0.005674	-7.57796	0.722412	9.53306
rChymotryp	H2AY_RAT	TAEILELAGI	56	81	H2AY_RAT_	4	5.06E-05	-14.5283	0.984628	6.777302
rChymotryp	H2AY_RAT	TAEILELAGI	56	81	H2AY_RAT_	4	1.14E-04	-13.4803	1.253172	9.296332
rChymotryp	H2AY_RAT	TVLSTKSLF	182	190	H2AY_RAT_	4	6.20E-05	-14.0497	0.543426	3.867881
rChymotryp	H2AY_RAT	VQEMAKLD	358	367	H2AY_RAT_	4	1.59E-04	-12.71	0.627189	4.934595
rChymotryp	H2AY_RAT	VQEM[15.9	358	367	H2AY_RAT_	4	6.14E-05	-14.0969	0.668151	4.739714
rChymotryp	H2AY_RAT	VSTM[15.99	334	346	H2AY_RAT_	4	9.58E-06	-16.826	0.859781	5.109851
rChymotryp	H2AZ_RAT	AGGKAGKD	1	25	H2AZ_RAT_	4	9.45E-04	-10.1265	0.526097	5.195238
rChymotryp	H2AZ_RAT	AGGKAGKD	1	36	H2AZ_RAT_	4	4.68E-05	-14.3882	0.129483	0.899922
rChymotryp	H2AZ_RAT	AGNASKDL	69	88	H2AZ_RAT_	4	6.07E-05	-14.0336	0.332453	2.368978
rChymotryp	H2AZ_RAT	AGNASKDL	69	88	H2AZ_RAT_	4	0.005185	-7.60043	0.185387	2.439166
rChymotryp	H2AZ_RAT	AIRGDEELD	89	99	H2AZ_RAT_	4	6.30E-04	-10.6358	0.123023	1.156689
rChymotryp	H2AZ_RAT	AIRGDEELD	89	117	H2AZ_RAT_	2	2.62E-05	-15.2344	0.307755	2.020139

rChymotryp	H2AZ_RAT	ELAGNASK	67	76	H2AZ_RAT_	4	2.69E-04	-11.8697	0.188117	1.584849
rChymotryp	H2AZ_RAT	ELAGNASK	67	86	H2AZ_RAT_	4	4.10E-04	-11.2831	0.350202	3.10377
rChymotryp	H2AZ_RAT	IKATIAGGG	100	117	H2AZ_RAT_	4	1.21E-05	-16.4047	0.52191	3.181472
rChymotryp	H2AZ_RAT	IKATIAGGG	100	127	H2AZ_RAT_	4	2.84E-04	-11.7836	0.119391	1.013194
rChymotryp	H2AZ_RAT	KSRTTSHGI	37	53	H2AZ_RAT_	4	2.22E-04	-12.1511	0.228633	1.881579
rChymotryp	H2AZ_RAT	KVKRITPRH	77	88	H2AZ_RAT_	4	8.77E-05	-13.6869	0.964955	7.050189
rChymotryp	H2AZ_RAT	QFPVGRIHI	26	36	H2AZ_RAT_	4	6.81E-05	-13.9911	0.762505	5.449939
rChymotryp	H2AZ_RAT	QLAIRGDEI	87	99	H2AZ_RAT_	4	2.70E-05	-15.2889	0.625637	4.092102
rChymotryp	H2B1A_RAT	ERIAGEASF	72	81	H2B1A_RAT	4	8.60E-04	-10.1967	0.231736	2.27265
rChymotryp	H2B1A_RAT	ERIAGEASF	72	84	H2B1A_RAT	4	0.022663	-5.50672	0.396978	7.208985
rChymotryp	H2B1A_RAT	E[-18.0106	72	81	H2B1A_RAT	4	6.38E-05	-14.1526	0.995638	7.035028
rChymotryp	H2B1A_RAT	E[-18.0106	72	84	H2B1A_RAT	4	2.19E-04	-12.3744	1.006588	8.134439
rChymotryp	H2B1A_RAT	KQVHPDTG	47	63	H2B1A_RAT	4	4.64E-05	-14.5633	0.807935	5.547733
rChymotryp	H2B1A_RAT	KQVHPDTG	47	63	H2B1A_RAT	3	1.89E-04	-12.4414	0.559203	4.494682
rChymotryp	H2B1A_RAT	KQVHPDTG	47	66	H2B1A_RAT	3	6.28E-05	-14.7873	2.254171	15.24393
rChymotryp	H2B1A_RAT	KVLKQVHP	44	63	H2B1A_RAT	2	6.14E-05	-14.0164	0.38077	2.716604
rChymotryp	H2B1A_RAT	KVLKQVHP	44	63	H2B1A_RAT	3	8.61E-05	-13.5224	0.291596	2.156393
rChymotryp	H2B1A_RAT	KVLKQVHP	44	63	H2B1A_RAT	4	8.29E-05	-13.561	0.115878	0.854497
rChymotryp	H2B1A_RAT	KVLKQVHP	44	63	H2B1A_RAT	2	0.000181	-12.4294	0.048613	0.391115
rChymotryp	H2B1A_RAT	KVLKQVHP	44	63	H2B1A_RAT	3	1.07E-04	-13.2066	0.251423	1.903768
rChymotryp	H2B1A_RAT	KVLKQVHP	44	63	H2B1A_RAT	3	3.46E-05	-14.8801	0.501774	3.372107
rChymotryp	H2B1A_RAT	PEVSAKGTT	1	15	H2B1A_RAT	4	7.05E-06	-17.1973	0.595766	3.464296
rChymotryp	H2B1_RAT	AHYNKRSTI	80	99	H2B1_RAT_	4	5.92E-05	-14.0682	0.304662	2.1656
rChymotryp	H2B1_RAT	AKHAVSEG	106	120	H2B1_RAT_	4	0.004863	-7.7083	0.302369	3.922636
rChymotryp	H2B1_RAT	GIMNSFVN	60	70	H2B1_RAT_	4	3.34E-04	-11.5664	0.268464	2.321061
rChymotryp	H2B1_RAT	GIM[15.994	60	70	H2B1_RAT_	4	1.90E-04	-12.4411	0.557871	4.484111
rChymotryp	H2B1_RAT	KQVHPDTG	46	59	H2B1_RAT_	4	0.006061	-7.36959	0.115698	1.569939
rChymotryp	H2B1_RAT	KQVHPDTG	46	62	H2B1_RAT_	4	4.81E-04	-11.0919	0.516618	4.657631
rChymotryp	H2B1_RAT	KQVHPDTG	46	65	H2B1_RAT_	4	0.00171	-9.19843	0.160677	1.746786
rChymotryp	H2B1_RAT	KQVHPDTG	46	65	H2B1_RAT_	4	1.30E-05	-16.2469	0.274665	1.690569
rChymotryp	H2B1_RAT	KQVHPDTG	46	62	H2B1_RAT_	4	9.33E-05	-13.3904	0.100475	0.750354
rChymotryp	H2B1_RAT	KQVHPDTG	46	65	H2B1_RAT_	4	3.40E-04	-11.5498	0.323737	2.802967
rChymotryp	H2B1_RAT	KQVHPDTG	46	59	H2B1_RAT_	4	0.001999	-8.97254	0.151621	1.689837
rChymotryp	H2B1_RAT	KQVHPDTG	46	62	H2B1_RAT_	4	2.56E-04	-11.9393	0.165869	1.389264
rChymotryp	H2B1_RAT	KQVHPDTG	46	65	H2B1_RAT_	4	0.002064	-8.93955	0.275382	3.080488
rChymotryp	H2B1_RAT	KQVHPDTG	46	65	H2B1_RAT_	4	2.06E-05	-15.6674	0.632614	4.03778
rChymotryp	H2B1_RAT	KQVHPDTG	46	62	H2B1_RAT_	4	5.45E-05	-14.1669	0.132746	0.937011
rChymotryp	H2B1_RAT	KQVHPDTG	46	65	H2B1_RAT_	4	6.43E-04	-10.6264	0.31263	2.942021
rChymotryp	H2B1_RAT	KVLKQVHP	43	59	H2B1_RAT_	4	0.00841	-6.9106	0.252964	3.660519
rChymotryp	H2B1_RAT	KVLKQVHP	43	62	H2B1_RAT_	4	0.001281	-9.69861	0.570734	5.884694
rChymotryp	H2B1_RAT	KVLKQVHP	43	62	H2B1_RAT_	4	4.94E-04	-11.0103	0.311617	2.830224
rChymotryp	H2B1_RAT	KVLKQVHP	43	59	H2B1_RAT_	4	0.00393	-8.00504	0.227601	2.843224
rChymotryp	H2B1_RAT	KVLKQVHP	43	62	H2B1_RAT_	4	9.15E-04	-10.1019	0.181291	1.794624
rChymotryp	H2B1_RAT	KVLKQVHP	43	62	H2B1_RAT_	4	2.40E-04	-12.0424	0.279351	2.319732
rChymotryp	H2B1_RAT	KVLKQVHP	43	59	H2B1_RAT_	4	2.70E-04	-11.9473	0.609971	5.10551
rChymotryp	H2B1_RAT	KVLKQVHP	43	62	H2B1_RAT_	4	3.50E-05	-15.0148	0.91983	6.126155
rChymotryp	H2B1_RAT	KVLKQVHP	43	59	H2B1_RAT_	4	3.99E-05	-14.6865	0.532142	3.623347
rChymotryp	H2B1_RAT	KVLKQVHP	43	62	H2B1_RAT_	3	1.90E-05	-15.6956	0.248811	1.585228
rChymotryp	H2B1_RAT	KVLKQVHP	43	59	H2B1_RAT_	4	2.47E-05	-15.4041	0.615767	3.997432
rChymotryp	H2B1_RAT	K[114.0317	43	62	H2B1_RAT_	4	9.68E-06	-16.8127	0.785543	4.67233

rChymotryp	H2B1_RAT	K[27.9949]T	43	59	H2B1_RAT_	3	8.34E-06	-17.2351	1.424631	8.265893
rChymotryp	H2B1_RAT	K[27.9949]T	43	59	H2B1_RAT_	2	6.51E-06	-17.2294	0.065643	0.380993
rChymotryp	H2B1_RAT	LPGELAKHA	101	120	H2B1_RAT_	4	1.08E-04	-13.2166	0.386707	2.925913
rChymotryp	H2B1_RAT	LPGELAK[4	101	120	H2B1_RAT_	4	0.009044	-6.85984	0.533903	7.783021
rChymotryp	H2B1_RAT	NKRSTITSR	83	99	H2B1_RAT_	4	0.032315	-4.96345	0.210219	4.235353
rChymotryp	H2B1_RAT	NKRSTITSR	83	100	H2B1_RAT_	4	2.14E-04	-12.2728	0.532312	4.337334
rChymotryp	H2B1_RAT	NKRS[-18.0	83	99	H2B1_RAT_	4	8.59E-05	-13.593	0.588177	4.32705
rChymotryp	H2B1_RAT	NK[27.9949	83	99	H2B1_RAT_	3	1.68E-05	-15.8686	0.165442	1.042577
rChymotryp	H2B1_RAT	PGELAKHAY	102	120	H2B1_RAT_	4	9.07E-04	-10.163	0.469898	4.623602
rChymotryp	H2B1_RAT	VYKVLKQVI	41	59	H2B1_RAT_	3	1.26E-05	-16.8755	1.911502	11.32707
rChymotryp	H31_RAT	ALQEACEAY	91	99	H31_RAT_A	4	2.55E-04	-11.9582	0.268736	2.247302
rChymotryp	H31_RAT	QSSAVM[15	85	92	H31_RAT_Q	4	1.03E-05	-16.5989	0.37802	2.277374
rChymotryp	H31_RAT	QSSAVM[15	85	99	H31_RAT_Q	4	7.37E-05	-13.8115	0.539647	3.907233
rChymotryp	H31_RAT	RFQSSAVM	83	90	H31_RAT_R	4	8.89E-05	-13.552	0.573581	4.232434
rChymotryp	H33_RAT	EDTNLCAIH	105	126	H33_RAT_E	4	0.003602	-8.23336	0.63153	7.670387
rChymotryp	H33_RAT	EDTNLCAIH	105	126	H33_RAT_E	4	0.004113	-7.99655	0.509934	6.376923
rChymotryp	H33_RAT	E[-18.0106	105	126	H33_RAT_E	4	7.03E-05	-13.8888	0.593519	4.273348
rChymotryp	H33_RAT	E[-18.0106	105	126	H33_RAT_E	3	2.93E-04	-11.8229	0.588464	4.977305
rChymotryp	H33_RAT	IRKLPF	62	67	H33_RAT_IF	4	0.028954	-5.13568	0.315108	6.135651
rChymotryp	H33_RAT	IRKLPFQRL	62	70	H33_RAT_IF	4	0.001711	-9.21036	0.267354	2.902748
rChymotryp	H33_RAT	KTDLRF	79	84	H33_RAT_K	4	0.00739	-7.08402	0.121068	1.70903
rChymotryp	H33_RAT	K[14.0156]T	79	84	H33_RAT_K	4	0.003699	-8.09484	0.248824	3.07386
rChymotryp	H33_RAT	K[28.0313]T	79	84	H33_RAT_K	4	0.001681	-9.22142	0.134733	1.461093
rChymotryp	H33_RAT	LIRKLPF	61	67	H33_RAT_L	4	4.16E-05	-14.5545	0.082513	0.566925
rChymotryp	H33_RAT	QKSTELL	55	61	H33_RAT_Q	4	0.015416	-6.0358	0.249608	4.135459
rChymotryp	H33_RAT	QKS[-18.01	55	61	H33_RAT_Q	4	1.93E-04	-12.3398	0.093447	0.757277
rChymotryp	H33_RAT	QRLVREIAQ	68	78	H33_RAT_Q	4	0.009118	-6.8164	0.397571	5.832566
rChymotryp	H33_RAT	QRLVREIAQ	68	82	H33_RAT_Q	4	3.22E-04	-11.6571	0.474501	4.070504
rChymotryp	H33_RAT	QRLVREIAQ	68	82	H33_RAT_Q	4	2.49E-06	-18.8413	1.014139	5.382538
rChymotryp	H33_RAT	QSAAIGALQ	85	99	H33_RAT_Q	4	0.013447	-6.25951	0.396589	6.33578
rChymotryp	H33_RAT	QSAAIGALQ	85	99	H33_RAT_Q	4	3.30E-05	-15.333	1.580176	10.30572
rChymotryp	H33_RAT	QS[-18.010	85	99	H33_RAT_Q	4	5.09E-06	-17.5929	0.193471	1.099711
rChymotryp	H33_RAT	RFQSAAIGA	83	99	H33_RAT_R	4	4.78E-04	-11.0426	0.219205	1.985083
rChymotryp	H33_RAT	RPGTVALRE	42	54	H33_RAT_R	4	0.01221	-6.37229	0.253195	3.973374
rChymotryp	H33_RAT	VREIAQDF	71	78	H33_RAT_V	4	0.003289	-8.28643	0.3801	4.587023
rChymotryp	H33_RAT	VREIAQDFK	71	82	H33_RAT_V	4	3.76E-04	-11.3807	0.092013	0.808504
rChymotryp	H33_RAT	VREIAQDFK	71	84	H33_RAT_V	4	0.001738	-9.30312	0.680539	7.315164
rChymotryp	H33_RAT	VREIAQDFK	71	82	H33_RAT_V	4	8.20E-05	-13.648	0.508104	3.722906
rChymotryp	H33_RAT	VREIAQDFK	71	84	H33_RAT_V	4	4.68E-04	-11.1032	0.38942	3.507289
rChymotryp	H33_RAT	VREIAQDFK	71	82	H33_RAT_V	3	1.94E-05	-15.6552	0.088955	0.568214
rChymotryp	H33_RAT	VREIAQDFK	71	84	H33_RAT_V	4	1.53E-04	-12.7713	0.581899	4.556297
rChymotryp	H4_RAT	ALKRQGRT	89	98	H4_RAT_AL	4	0.068189	-3.88689	0.218264	5.615397
rChymotryp	H4_RAT	ARRGGVKR	38	49	H4_RAT_AR	4	1.22E-04	-13.0274	0.320251	2.45829
rChymotryp	H4_RAT	ARRGGVKR	38	51	H4_RAT_AR	4	3.79E-05	-14.8569	0.804468	5.414793
rChymotryp	H4_RAT	EETRGVL	52	58	H4_RAT_EE	4	0.001397	-9.527	0.420582	4.41463
rChymotryp	H4_RAT	EETRGVLKV	52	61	H4_RAT_EE	4	3.98E-05	-14.6389	0.301035	2.056404
rChymotryp	H4_RAT	ENVIRDAVT	63	72	H4_RAT_EN	4	0.019793	-5.66369	0.137011	2.419104
rChymotryp	H4_RAT	E[-18.0106	52	58	H4_RAT_E[-	3	3.13E-05	-15.0053	0.42344	2.821931
rChymotryp	H4_RAT	E[-18.0106	63	72	H4_RAT_E[-	4	9.85E-05	-13.5654	0.934456	6.888536
rChymotryp	H4_RAT	IYEETRGVL	50	58	H4_RAT_IYE	4	0.044932	-4.50395	0.326035	7.238851

rChymotryp	H4_RAT	IYEETRGVL	50	61	H4_RAT_IYE	4	4.26E-04	-11.2659	0.500997	4.447035
rChymotryp	H4_RAT	KRQGRTLY	91	98	H4_RAT_KR	4	4.26E-04	-11.2078	0.199535	1.780314
rChymotryp	H4_RAT	KVFLENVIR	59	72	H4_RAT_KV	4	0.009068	-6.87225	0.570772	8.305461
rChymotryp	H4_RAT	LENVIRDAV	62	72	H4_RAT_LE	4	0.015015	-6.06034	0.105269	1.737019
rChymotryp	H4_RAT	RDNIQGITK	23	49	H4_RAT_RD	4	5.62E-05	-14.1359	0.25687	1.817153
rChymotryp	H4_RAT	TEHAKRKTV	73	88	H4_RAT_TE	4	0.035675	-4.81543	0.157062	3.261647
rChymotryp	H4_RAT	TEHAKRKTV	73	88	H4_RAT_TE	4	0.009676	-6.72494	0.353932	5.262981
